# Combinatorial RNA interference in *Caenorhabditis elegans *reveals that redundancy between gene duplicates can be maintained for more than 80 million years of evolution

**DOI:** 10.1186/gb-2006-7-8-r69

**Published:** 2006-08-02

**Authors:** Julia Tischler, Ben Lehner, Nansheng Chen, Andrew G Fraser

**Affiliations:** 1 The Wellcome Trust Sanger Institute, Hinxton, Cambridge, CB10 1SA, UK; 2 CRG-EMBL Systems Biology Program, Centre for Genomic Regulation, Barcelona, Spain; 3 Molecular Biology and Biochemistry, Simon Fraser University, University Drive, Burnaby, British Columbia, V5A 1S6, Canada

## Abstract

**Background:**

Systematic analyses of loss-of-function phenotypes have been carried out for most genes in *Saccharomyces cerevisiae*, *Caenorhabditis elegans*, and *Drosophila melanogaster*. Although such studies vastly expand our knowledge of single gene function, they do not address redundancy in genetic networks. Developing tools for the systematic mapping of genetic interactions is thus a key step in exploring the relationship between genotype and phenotype.

**Results:**

We established conditions for RNA interference (RNAi) in *C. elegans *to target multiple genes simultaneously in a high-throughput setting. Using this approach, we can detect the great majority of previously known synthetic genetic interactions. We used this assay to examine the redundancy of duplicated genes in the genome of *C. elegans *that correspond to single orthologs in *S. cerevisiae *or *D. melanogaster *and identified 16 pairs of duplicated genes that have redundant functions. Remarkably, 14 of these redundant gene pairs were duplicated before the divergence of *C. elegans *and *C. briggsae *80-110 million years ago, suggesting that there has been selective pressure to maintain the overlap in function between some gene duplicates.

**Conclusion:**

We established a high throughput method for examining genetic interactions using combinatorial RNAi in *C. elegans*. Using this technique, we demonstrated that many duplicated genes can retain redundant functions for more than 80 million years of evolution. This provides strong support for evolutionary models that predict that genetic redundancy between duplicated genes can be actively maintained by natural selection and is not just a transient side effect of recent gene duplication events.

## Abstract 2

High-throughput combinatorial RNAi demonstrates that many duplicated genes in *C. elegans *can retain redundant functions for more than 80 million years

## Background

One of the most direct approaches to elucidating the role of any particular gene is to characterize its loss-of-function phenotype. Loss-of-function phenotypes have now been analyzed for almost all of the predicted genes of *Saccharomyces cerevisiae *[1], *Caenorhabditis elegans *[2], and *Drosophila melanogaster *[3], and there are ongoing efforts to make comprehensive collections of mouse knockouts. In all, this gives us an unprecedented level of insight into eukaryotic gene function. However, the loss-of-function phenotype of any individual gene is highly dependent on the genetic context; specifically, variations in the activities of other genes will affect this phenotype (for review [4]). If changes in the activity of one gene affect the loss-of-function phenotype of a second gene, then these two genes are said to interact genetically. Genetic interactions can be used to identify novel components of molecular pathways and can reveal the redundancy that underlies the robustness of genetic networks. Thus, although analyzing the loss-of-function phenotypes of all genes in a wild-type animal is a major advance, an understanding of how each phenotype is modulated by the activities of other genes will prove to be just as critical.

Recently, genetic interactions in *S. cerevisiae *were investigated in a systematic manner using matings within a comprehensive collection of mutant strains. Pair-wise matings have identified over 4500 genetic interactions, demonstrating the extensive degree of redundancy in yeast [5,6]. However, this approach is not currently feasible in any animal. No complete collection of mutant strains exists, and even if such strains were all available, large-scale matings are far more laborious in animals than in yeast, and so alternative strategies are needed.

One underlying cause of genetic redundancy may be gene duplication. Duplicated genes that retain at least partially overlapping functions can confer robustness to mutation in the other copy [7,8]. However, there is still much debate about whether redundancy of duplicated genes can be evolutionary selected [9-11]. Theoretical models have been proposed to explain the evolutionary stability of redundancy [12,13], and indirect experimental evidence for the redundant functions of duplicated genes comes from the analysis of loss-of-function phenotypes of single genes; in both yeast and worms, inactivation of a duplicated gene is less likely to result in a nonviable phenotype than inactivation of a single copy gene [2,14,15]. However, there are strong biases in the types of genes that are duplicated in genomes, which complicates the interpretation of these results [16], and no attempt has yet been made to examine the extent of redundancy between duplicated genes *in vivo *directly and systematically.

RNA-mediated interference (RNAi) is a powerful tool for studying the loss-of-function phenotypes of genes. In particular, in *C. elegans*, RNAi by bacterial feeding has been used for genome-wide screens because it allows high-throughput (HTP) and low-cost analysis of the loss-of-function phenotypes of genes *in vivo *[2]. However, RNAi has only been used extensively to target single genes. To study genetic redundancy systematically and to identify genetic interactions using RNAi, it is critical to establish and validate robust methods for simultaneously targeting multiple genes by RNAi using bacterial feeding ('combinatorial RNAi'). In the present report we show that by using combinatorial RNAi by bacterial feeding we can identify the majority of a testset of previously described genetic interactions. We used this technique to provide the first large-scale analysis of the redundant functions of duplicated genes in any organism, and we found that many duplicate gene pairs can retain redundant functions for more than 80 million years of evolution.

## Results

### Effectiveness of combinatorial RNA-mediated interference

We sought to establish HTP methods for simultaneously targeting multiple genes in *C. elegans *using RNAi by bacterial feeding ('combinatorial RNAi') on a large scale. We recently developed HTP methods for using RNAi by feeding to target single genes (see Materials and methods, below); these assays allow us to identify the vast majority (>85%) of previously published nonviable RNAi phenotypes with high reproducibility (>90%) [17,18]. We wished to determine whether we could adapt these methods, which are efficient for analyzing the RNAi phenotypes of single genes, to targeting multiple genes by combinatorial RNAi.

**Figure 1:**
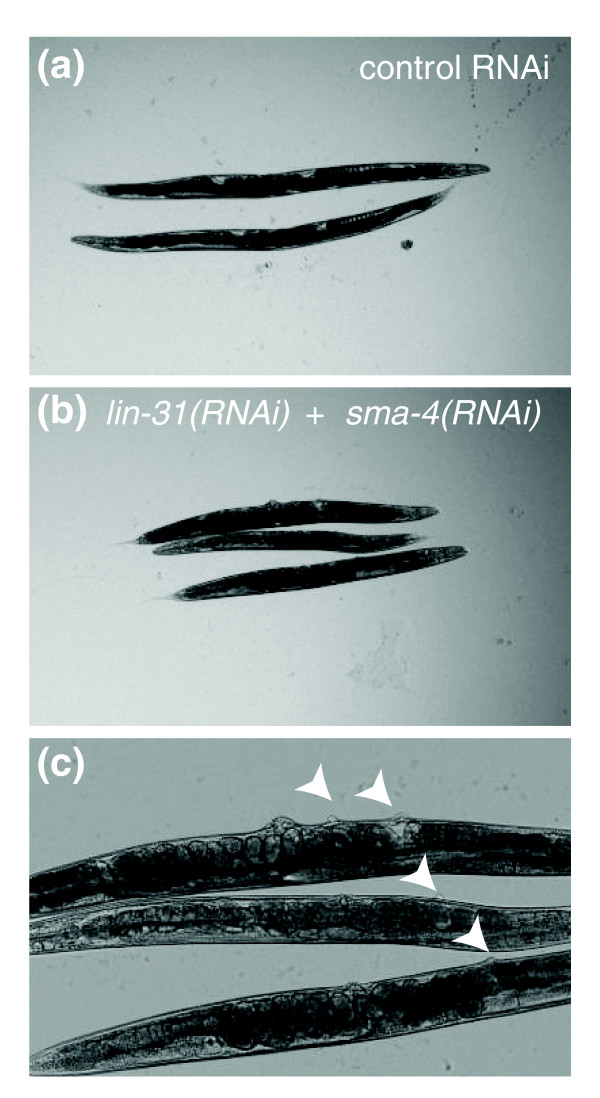
Combinatorial RNA interference (RNAi) can target two genes in the same animal. Exposing worms to a mixture of two double-stranded (ds)RNA-expressing bacterial clones, one targeting *lin-31 *and the other one targeting *sma-4*, resulted in small worms with multiple vulvae along their ventral side. Shown are RNAi-hypersensitive *rrf-3 *animals [19] fed on bacteria expressing **(a) **a nontargeting dsRNA (control) and **(b) **combined bacterial clones expressing dsRNA against *lin-31 *and *sma-4 *(magnified in **(c)**). Pseudovulvae are indicated by white arrowheads.

To investigate whether we could target effectively more than one gene in a single animal using bacterial-mediated RNAi, we used three tests. First, we assessed whether we could simultaneously target two independent genes, each with a known loss-of-function phenotype, and generate phenotypes for both genes in the same animal. For example, targeting *lin-31 *by RNAi generates multivulval worms, targeting *sma-4 *generates small worms, and targeting both would be expected to generate small worms with multiple vulvae if combinatorial RNAi is effective. We chose well characterized genes with non-overlapping phenotypes (Table 1) to ensure that we could investigate each phenotype independently. We examined all possible pair-wise combinations of our four test genes either in wild-type animals or in the RNAi-hypersensitive strain *rrf-3 *[19], and scored for the known RNAi phenotypes. We found that we could detect five of the five possible additive phenotypes in both wild-type and *rrf-3 *worms (Table 1; see Figure 1 for an example), demonstrating that it is feasible to target two genes in the same animal by bacterial-mediated RNAi. In addition to generating additive phenotypes, we found that the simultaneous targeting of *sma-4 *and *lon-2 *produced only small worms (the phenotype of *sma-4 *alone). Thus, we can use combinatorial RNAi to recapitulate a previously demonstrated epistatic relationship between SMADs and *lon-2 *[20]. Finally, although we could detect additive RNAi phenotypes in wild-type worms, we noted that the penetrance was often higher in the *rrf-3 *RNAi-hypersensitive strain, suggesting that this background might be more suitable for combinatorial RNAi; we examine this in more detail below.

**Figure 2:**
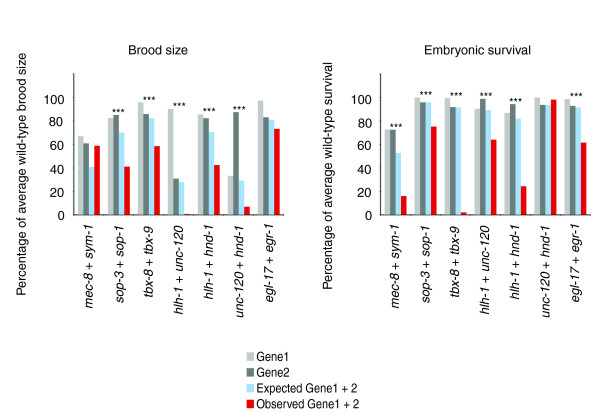
Combinatorial RNA interference (RNAi) can recapitulate known synthetic lethal interactions. To test whether combinatorial RNAi could recapitulate seven synthetic lethal interactions that were identified from literature (see Table 2 for references), brood size and embryonic survival measurements following co-targeting of both genes of a synthetic lethal pair (Observed Gene1 + 2) were compared with that following the targeting of each single gene alone (Gene1 or Gene2) and with the calculated product of the single gene brood sizes and embryonic survival measurements (Expected Gene1 + 2); this product represents the predicted outcome if the genetic interaction is purely additive. Values plotted represent the percentage of average wild-type brood size and embryonic survival rates, and are the arithmetic mean of two independent experiments performed in the RNAi-hypersensitive strain *rrf-3 *[19]. ***p < 1.0 × 10^-02^; *p < 5.0 × 10^-02^, by Student's t-test.

We next tested a set of known synthetic lethal interactions compiled from literature [21-25] (Table 2 and Figure 2). In *rrf-3 *animals, we were able to detect reproducibly all seven tested genetic interactions (Table 2 and Figure 2). However, in wild-type animals only five of these interactions could be recapitulated (Table 2). Not only did we fail to detect two out of seven interactions in wild-type worms, the five detected interactions were also weaker than in *rrf-3*, demonstrating that for effective combinatorial RNAi it is often essential to use RNAi-hypersensitive strains.

Finally, we investigated whether we could use combinatorial RNAi to recapitulate known genetic interactions that result in post-embryonic phenotypes. To do this we focused on the well characterized synthetic multivulval (synMuv) genes [26-28]. The synMuv genes are organized into two redundant genetic pathways that are required for normal development of the hermaphrodite vulva. Inactivation of either a synMuv A pathway gene or a synMuv B pathway gene alone results in no vulval defect, but inactivation of both a synMuv A and a synMuv B gene in combination results in the multivulva (Muv) phenotype. Using combinatorial RNAi, we co-targeted three synMuv A genes with the canonical class B gene *lin-15B*, and co-targeted 12 synMuv B genes with the canonical synMuv A gene *lin-15A *in either wild-type or *rrf-3 *animals. In each experiment, we scored progeny for the multivulva phenotype; we expected to see this phenotype only if combinatorial RNAi targets both genes effectively in the same animal. We observed Muv worms for 13 out of 15 test cases in the RNAi-hypersensitive *rrf-3 *background, and for 8 out of 15 possible viable combinations in wild-type animals (Table 3).

Taken together these results demonstrate that combinatorial RNAi by feeding using our HTP platform works efficiently in *rrf-3 *animals; we were able to generate additive phenotypes and to detect the great majority of previously described genetic interactions.

### Effect of dilution on phenotype strength

**Figure 3:**
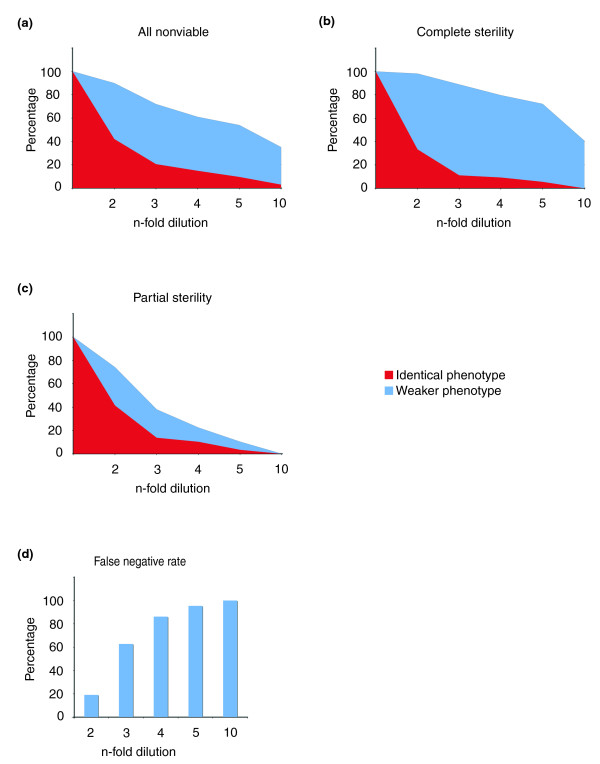
Effect of dilution on strength of RNA interference (RNAi) phenotype. The RNAi phenotype of each nonviable gene on chromosome III [2] was assessed following dilution with increasing amounts of bacteria expressing a nontargeting double-stranded (ds)RNA. The percentage of genes with phenotypes that are either identical to that observed when targeted alone (red) or weaker than when targeted alone (blue) is shown for each dilution. This was examined for three phenotypes: **(a) **all nonviable phenotypes, **(b) **complete sterility (no progeny), and **(c) **partial sterility (some progeny). **(d) **False negative rate (in percentage) of combinatorial RNAi at a given dilution. Data shown are representative of two independent experiments performed in the RNAi-hypersensitive *rrf-3 *background [19].

In analyzing the phenotypes produced through combinatorial RNAi, we and others [29,30] observed that some of the single gene phenotypes were qualitatively weaker when two genes were targeted together than when each gene was targeted alone. Because such dilution effects will affect both the false negative rate in large-scale screens and the possible number of genes that can be co-targeted effectively, we wished to investigate the extent to which combining double-stranded (ds)RNA-expressing bacteria leads to reduced strength of RNAi phenotypes. To do this, we selected 282 genes from chromosome III that have a nonviable (embryonic lethal or sterile) RNAi phenotype [2] (Additional data file 1) and examined whether their phenotypes change as the targeting bacteria are diluted with increasing amounts of unrelated dsRNA-expressing bacteria (Figure 3).

We found that the strength of RNAi phenotypes for many genes was indeed reduced with increasing dilution of control bacteria (Figure 3). For example, we were able to detect phenotypes for about 90% of genes with nonviable RNAi phenotypes (Figure 3a) when the targeting strains were diluted with equal amounts of a bacterial strain expressing a control nontargeting dsRNA. This detection rate dropped further to about 70% at threefold and to about 60% at fourfold dilution (Additional data file 1). We found essentially identical results when we diluted with a dsRNA-expressing bacterial strain targeting *lin-31 *(data not shown), showing that the observed dilution effect appears not to be specific to the diluting dsRNA-expressing strain.

We next considered whether the effect of dilution on the observed phenotype was related to phenotypic strength. To this end, we determined the dilution behavior for genes that have different strengths of brood size defects when targeted alone (Figure 3b,c). We found that genes with weak RNAi phenotypes were indeed more likely to appear wild-type following dilution - and thus to be missed in screens - than were genes with strong, highly penetrant phenotypes. For example, we could still detect phenotypes for about 80% of genes that normally have a completely sterile phenotype at a fourfold dilution; however, only about 20% of genes conferring partial sterility (a reduction in brood size) had a detectable phenotype at this dilution. Although this indicates that genes with weaker phenotypes are more likely to appear wild-type when targeted in combination with other genes, we conclude that on average about 90% of genes with a detectable RNAi phenotype still have sufficient knockdown when diluted with equal amounts of a second dsRNA-expressing bacterial strain.

Overall, these experiments allow us to estimate the false-negative rates induced by dilution effects in combinatorial RNAi (Figure 3d; see Materials and methods for calculation). Assuming that each gene behaves independently, we expect that about 80% of bigenic interactions yielding visible RNAi phenotypes will be detectable by combinatorial RNAi. Because RNAi in *rrf-3 *recapitulates null phenotypes for about 70% of known genetic nulls, we thus estimate that combinatorial RNAi can detect about 50% of all bigenic interactions yielding nonviable phenotypes.

### Investigating the redundancy of duplicated genes in *C. elegans*

Having validated combinatorial RNAi by using bacterial feeding as a method to inhibit simultaneously the expression of any pair-wise combination of genes, we wished to use this approach to investigate functional redundancy in the genome of *C. elegans*. One obvious possible cause of genetic redundancy is through gene duplication. Duplicated genes that have retained at least partially overlapping functions can confer robustness to mutation in the other copy [7,8], and genome-wide loss-of-function screens provide indirect evidence that duplicated genes may often share redundant functions [2,14,15]. However, this hypothesis has never been directly tested with systematic experimental approaches.

**Figure 4:**
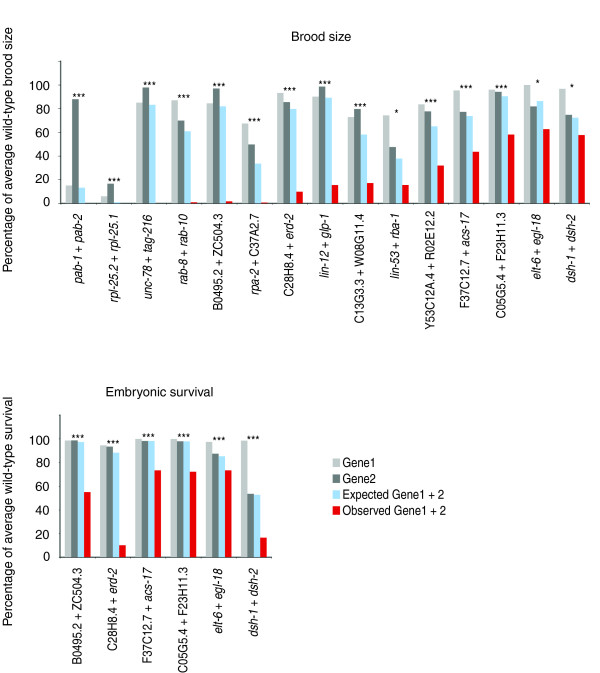
Quantitative analysis of synthetic phenotypes following the simultaneous targeting of both genes of a duplicate pair. For duplicate gene pairs that yielded reproducible synthetic effects, phenotypes produced by combinatorial RNA interference (RNAi) were quantitated. For each gene pair, brood size and embryonic survival following co-targeting of both duplicates (Observed Gene1 + 2) were compared with that following the targeting of each single gene alone (Gene1 or Gene2) and with the calculated product of the single gene brood sizes and embryonic survival measurements (Expected Gene1 + 2). Values plotted represent the percentage of average wild-type brood size and embryonic survival rates, respectively, and are the arithmetic mean of two independent experiments performed in the RNAi-hypersensitive strain *rrf-3 *[19]. ***p < 1.0 × 10^-02^, *p < 5.0 × 10^-02^, by Student's t-test. Note that combinatorial RNAi against the gene pair *ptr-2 *+ *ptr-10 *resulted in a significantly increased number (*p *= 7.4 × 10^-09^, by Student's t-test) of first-generation larval growth arrested worms, rather than a brood size defect, hence these data are not shown.

We used the InParanoid algorithm [31] to identify 239 pairs of *C. elegans *genes that correspond to single orthologs in *S. cerevisiae *or *D. melanogaster *genomes (see Materials and methods, below). These genes have thus been duplicated in the genome of *C. elegans *since the divergence from either species. To determine whether there is functional redundancy between the duplicated genes, we compared the phenotype resulting from targeting both duplicated genes simultaneously by RNAi with the RNAi phenotype of each gene alone. We interpret a synthetic genetic interaction - that is, where the combined phenotype is greater than the product of the individual phenotypes [32] - as indicating redundancy. Of 143 duplicate gene pairs amenable to analysis by combinatorial RNAi (see Materials and methods, below; Additional data file 2), we found 16 pairs of duplicated genes to show reproducible synthetic RNAi phenotypes by quantitation (Table 4 and Figure 4), indicating that they are, at least in part, functionally redundant. Of these pairs only two have previously been identified as having redundant functions [33,34]. The pairs of genes that when co-targeted give synthetic phenotypes encode diverse molecular functions, ranging from structural constituents of the ribosome (for example, *rpa-2 *+ C37A2.7, *rpl-25.1 *+ *rpl-25.2*), signaling proteins (for example, *lin-12 *+ *glp-1*, C13G3.3 + W08G11.4), and transcription factors (for example, *elt-6 *+ *egl-18*) to polyadenylate-binding proteins (for example, *pab-1 *+ *pab-2*; Table 5).

The duplicated genes that we focused on in the worm correspond to single genes in either *S. cerevisiae *or *D. melanogaster *genomes. We wished to investigate whether the known function of the single yeast or fly gene was a good predictor of the RNAi phenotype identified by co-targeting the duplicated worm genes with redundant functions. If this were the case, then it is most likely that the redundancy that we observed is due to both duplicates retaining the ancestral function. Based on the gene deletion phenotypes of the single copy orthologs in yeast, we split our set of *C. elegans *duplicated genes into those corresponding to essential and to nonessential *S. cerevisiae *genes (Additional data file 2). We found that five out of 18 worm duplicates (28%) that are orthologous to yeast essential genes exhibited synthetic phenotypic effects by combinatorial RNAi. In contrast, only five out of 55 *C. elegans *duplicated genes (9%) that are orthologous to *S. cerevisiae *nonessential genes were found to produce a synthetic phenotype when co-targeted. We conclude that duplicated genes in *C. elegans *that are related to an essential gene in yeast are about three times more likely to have an essential redundant function than those related to a nonessential yeast gene. Strikingly, this is the same enrichment for nonviable RNAi phenotypes as for nonduplicated genes; 61% of *C. elegans *single copy orthologs of *S. cerevisiae *essential genes have nonviable RNAi phenotypes, as compared with 20% of orthologs of yeast nonessential genes (Additional data file 3). Thus, our finding is entirely consistent with a simple model of redundancy, suggesting that the function of a single gene identified in one organism is a good predictor of the redundant function covered by a pair of duplicated genes in a second organism.

### Duplicated genes can maintain redundant functions for more than 80 million years of evolution

By using combinatorial RNAi we found that 11% of *C. elegans *duplicate gene pairs corresponding to single yeast or fly genes had synthetic phenotypes. These data clearly demonstrate that duplicated genes in metazoans often have at least partially redundant functions, but they do not address the underlying causes for this redundancy. Two simple models might explain why some duplicated genes appear to have redundant functions. First, the redundancy may represent a transient state resulting from a recent duplication event. In this model, the pairs of genes we found to be redundant are likely to be more recent duplicates than those for which we found no functional overlap. Alternatively, several groups have established population-genetic frameworks suggesting that redundant functions can be maintained by natural selection over substantial evolutionary times [12,13]. In this case, we would expect no difference in age between the sets of duplicated genes for which we observed redundant phenotypes and gene pairs with no apparent redundant functions. Instead, we anticipated that there would be evidence that the redundant duplicated genes have been maintained relative to their ancestral sequence, thus retaining their overlapping, redundant functions.

Remarkably, 14 out of the 16 pairs of duplicated genes that we identified as having redundant essential functions in *C. elegans *were duplicated before the divergence from the related nematode *C. briggsae *(see Materials and methods, below; Additional data file 4). *C. elegans *and *C. briggsae*, despite being morphologically very similar, last shared a common ancestor 80-110 million years ago [35]. It is extremely unlikely that the redundancy between these 14 genes has been maintained for more than 80 million years of evolution merely as a consequence of the rate of neutral evolution, that is, that there has been insufficient evolutionary time for the duplicates to drift. To place this time period in the context of the rate of change of coding genes, *C. elegans *and *C. briggsae *only share about 60% of their genes as 1:1 orthologs, and a full 10% of genes encoded in either genome has no identifiable match in the other genome [35]. We thus considered the possibility that these 14 gene pairs retained redundant functions simply as a result of neutral evolution to be very unlikely; instead, these data suggest that the redundancy between these duplicated genes has been maintained over an extensive evolutionary period.

If there has been selection for the maintenance of redundancy between two duplicated genes, then we would expect these duplicates to encode more similar proteins than non-redundant duplicates. Indeed, we found that pairs of redundant duplicated genes are more similar to each other at the amino acid level (*p *= 1.6 × 10^-02^, by Wilcoxon rank sum test), have a greater similarity in alignable protein length (*p *= 2.2 × 10^-02^), and also exhibit a lower rate of nonsynonymous nucleotide substitution per nonsynonymous site (mean Ka for redundant duplicates = 0.34; mean Ka for non-redundant duplicates = 0.50; *p *= 3.8 × 10^-02^) than non-redundant duplicates (Additional data file 4). Using the rate of synonymous nucleotide substitutions (Ks) as a measure of the evolutionary age of gene duplicates, we found no evidence that the redundant genes represent more recent gene duplications (mean Ks = 13.41 for redundant duplicates, mean Ks = 9.48 for non-redundant duplicates; Additional data file 4). Thus, we believe that it is unlikely that this greater similarity is a trivial consequence of their having duplicated more recently. Rather, we suggest that the protein sequences of redundant gene pairs have been maintained relative to each other since duplication as the result of selective pressure to maintain their redundant functions.

## Discussion

RNAi has emerged as a key technique for the analysis of the *in vivo *function of single genes in *C. elegans*. For the systematic identification of genetic interactions by RNAi, we have established and validated methods that allow us to study the loss-of-function RNAi phenotypes of any pair-wise combination of *C. elegans *genes in a high-throughput manner. We found that we can use this methodology to identify the great majority of a testset of previously known synthetic lethal and post-embryonic genetic interactions. This approach should therefore allow researchers to explore genetic interactions in the worm in a far more systematic manner than has been possible in the past.

We used our method to examine systematically the potentially redundant functions of duplicated genes in the genome of *C. elegans*, focusing on genes that correspond to single orthologs in *S. cerevisiae *or *D. melanogaster*. These genes have thus duplicated in the *C. elegans *genome since the divergence from either species. Of the 143 pairs of duplicate genes amenable to analysis by combinatorial RNAi, we identified 16 gene pairs that exhibited unambiguous synthetic RNAi phenotypes, demonstrating that they are at least partially functionally redundant. We found that just as single copy worm genes are more likely to have a nonviable RNAi phenotype if they are orthologous to an essential gene in yeast, duplicated worm genes are more likely to have a redundant essential function if they are co-orthologous to an essential yeast gene. It should therefore be possible to predict the redundant functions of many duplicated genes in higher organisms based on the functions of single copy orthologs in lower organisms.

Most intriguingly, the redundancy we observed between duplicated genes cannot simply be explained by a very recent duplication event; 14 of the 16 redundant gene pairs were duplicated before the divergence of *C. elegans *and *C. briggsae *80-110 million years ago [35]. The redundancy between these 14 gene pairs has therefore been maintained for more than 80 million years of evolution. We believe that it is extremely unlikely that the functional overlap between these 14 duplicated genes is present merely due to the lack of evolutionary time since duplication. Not only is the average half-life of a gene duplicate in eukaryotes typically about 4 million years [11] but also, over this time period, the *C. elegans *and *C. briggsae *genomes have diverged greatly; they only share about 60% of their genes as 1:1 orthologs, and a further 10% of genes are present exclusively in one or other genome [35]. Rather, our findings are consistent with population genetic simulations that demonstrate that under appropriate (but realistic) conditions it is possible to select, directly or indirectly, for redundancy between duplicates to be maintained [12].

## Conclusion

Our data provide the first systematic investigation into the redundancy of duplicated genes in any organism and strongly support models of gene evolution, which suggest that redundancy is not just a transient side effect of recent gene duplication but is instead a phenomenon that can be maintained over substantial periods of evolutionary time.

## Materials and methods

### Ninety-six-well liquid feeding assay

Selected bacterial strains of the *C. elegans *RNAi feeding library [2] were grown to saturation at 37°C in 96-well deep plates in 400 μl 2 × TY containing 100 μg/ml ampicillin. To induce dsRNA expression, 4 mmol/l IPTG (isopropyl-beta-D-thiogalactopyranoside) was added for 1 hour at 37°C before cultures were spun down at 3500 rpm for 5 min and finally resuspended in 400 μl of NGM (nematode growth medium) with 100 μg/ml ampicillin and 4 mmol/l IPTG. Finally, 10 (for wild-type N2) or 15 (for NL2099 *rrf-3 *[*pk1426*] II) L1-stage worms in 15 μl M9 buffer were aliquoted into each well of a 96-well flat-bottom plate and 40 μl of the resuspended bacterial cultures were added. For combinatorial RNAi feeding experiments, resuspended saturated cultures of different bacterial strains were mixed to give a final volume of 40 μl. Plates were incubated shaking at 150 rpm, 20°C, for 96 hours. Worms were scored for embryonic lethality, sterility, and growth defects using a dissecting microscope.

### Testing additive RNAi phenotypes and known synthetic genetic interactions

To score post-embryonic phenotypes (Table 1 and Table 3), L1 larvae from the 96-well liquid feeding assay were collected after 96 hours and allowed to develop further on 12-well NGM plates. Cultures were filtered through a 11 μm nylon mesh (MultiScreen™ Nylon Mesh, Millipore Corporation, Bedford, MA, USA) and L1 larvae were spotted onto 12-well NGM plates containing 100 μg/ml ampicillin and 1 mmol/l IPTG, seeded with bacteria expressing a nontargeting dsRNA (Ahringer library clone Y95B8A_84.g). Adult worms were scored after further incubation at 20°C for 72 hours. Because we were assessing second generation (post-embryonic) phenotypes, we had to exclude genes that resulted in sterility, embryonic lethality, or larval growth arrest after RNAi. Only genes that were (according to the above criteria) amenable to analysis in both wild-type worms and the RNAi-hypersensitive *rrf-3 *background could be included in the study.

### Estimation of false-negative rate of combinatorial RNAi

Assuming each gene is an independent targeting event in combinatorial RNAi, and having evaluated the average failure rate for the successful generation of a phenotypically detectable knockdown for single genes at a given dilution, we were able to estimate the false-negative rate of combinatorial RNAi for multigenic interactions. We calculated the detection rate of n-genic interactions to be x^n^, where x is the detection rate of single gene phenotypes at n-fold dilution.

### Identification of duplicated genes and synthetic phenotypes

We used the InParanoid algorithm (version 4.0) to identify *C. elegans *orthologs of *S. cerevisiae *and *D. melanogaster *genes [31]. All genes that are targeted by bacterial clones from the *C. elegans *whole-genome RNAi library [2] with inserts having more than 80% nucleotide identity over 200 base pairs with multiple predicted genes were excluded from the analysis. This is the threshold for cross-reaction used by Kamath and coworkers [2]. Furthermore, genes that resulted in first-generation larval growth arrest after RNAi were not included in the study for synthetic interactions, because this strong phenotype cannot be enhanced any further.

When screening for phenotypic differences between single gene and combinatorial RNAi knockdowns, single gene phenotypes (as references) were compared with combinatorial phenotypes side by side. To account for dilution effects arising from combining two dsRNA-expressing bacteria, equal amounts of nontargeting dsRNA-expressing bacteria were added to bacteria expressing dsRNA targeting the reference genes. Screens for synthetic phenotypic effects were performed at least twice in triplicates within independent assays. For synthetic interactions to be scored positive, synthetic phenotypes had to be unambiguous and reproducible in at least two independent RNAi experiments.

### Statistical analysis

For statistical analysis of the data, measurements of brood size and embryonic viability following RNAi were normalized to measurements obtained after RNAi against control genes that give no detectable phenotypes ('wild-type brood size' and 'wild-type embryonic survival'). To examine whether the combinatorial phenotypes were synthetic or merely additive, we compared the quantitative phenotypes following combinatorial RNAi with the calculated products of measurements for both individual genes of a pair. Duplicate brood size and embryonic survival measurements for two individual genes were multiplied to generate an array of 16 values that was compared with six measurements obtained for synthetic phenotypes, using a Student's t-test (two-tailed distribution, two-sample equal variance). In cases in which measurements for brood size and embryonic viability exceeded 100% of wild-type brood and viability, values were set to 100% of wild-type values.

### Evolutionary analysis

We used InParanoid (version 4.0) to identify *C. elegans *orthologs of *S. cerevisiae*, *D. melanogaster*, and *C. briggsae *genes [31]. If both *C. elegans *duplicates had a single identifiable ortholog in *C. briggsae*, then this implies that the duplication predates the divergence of *C. elegans *from *C. briggsae*. Protein sequences were aligned using the CLUSTAL W program to determine the percentage of identity between gene duplicates [36].

## Additional data files

The following additional data are included with the online version of this article: A Word document listing *C. elegans *chromosome III genes with a previously assigned nonviable (embryonic lethal or sterile) RNAi phenotype [2] and the effect of dilution following combinatorial RNAi (Additional data file 1); a Word document listing *C. elegans *pairs of duplicated genes that have been screened for synthetic RNAi phenotypes (Additional data file 2); a Word document listing *C. elegans *1:1 orthologs of *S. cerevisiae *genes and their RNAi phenotypes (Additional data file 3); and a Word document presenting *C. elegans *duplicate gene pairs included in this study, their orthologous genes in *C. briggsae*, and levels of protein identity between *C. elegans *gene duplicates, as well as Ka and Ks values between duplicate gene pairs (Additional data file 4).

## Supplementary Material

Additional data file 1 A Word document listing *C. elegans *chromosome III genes with a previously assigned nonviable (embryonic lethal or sterile) RNAi phenotype [2] and the effect of dilution following combinatorial RNAi. Click here for file

Additional data file 2 A Word document listing *C. elegans *pairs of duplicated genes that have been screened for synthetic RNAi phenotypes. Click here for file

Additional data file 3 A Word document listing *C. elegans *1:1 orthologs of *S. cerevisiae *genes and their RNAi phenotypes. Click here for file

Additional data file 4 A Word document presenting *C. elegans *duplicate gene pairs included in this study, their orthologous genes in *C. briggsae*, and levels of protein identity between *C. elegans *gene duplicates, as well as Ka and Ks values between duplicate gene pairs. Click here for file

## Figures and Tables

**Table 1:** Combinatorial RNAi effectively generates additive phenotypes

Gene1	Gene2	Wild-type		*rrf-3*	
		
		Pheno Gene1	Pheno Gene2	Pheno Gene1	Pheno Gene2
*lin-31*	-	5%	-	35%	-
*sma-4*	-	100%	-	100%	-
*unc-22*	-	100%	-	100%	-
*lon-2*	-	100%	-	100%	-
*lin-31*	*sma-4*	2%	100%	20%	100%
*lin-31*	*unc-22*	2%	100%	26%	100%
*lin-31*	*lon-2*	4%	100%	13%	100%
*sma-4*	*unc-22*	100%	100%	100%	100%
*sma-4*	*lon-2*	100%	0%	100%	0%
*unc-22*	*lon-2*	100%	100%	100%	100%

Wild-type and RNA interference (RNAi)-hypersensitive *rrf-3 *worms, respectively, were fed on selected bacterial strains of the *C. elegans *RNAi feeding library [2] targeting the genes *lin-31*, *sma-4*, *unc-22*, and *lon-2*. Independent RNAi phenotypes (Pheno Gene1, Pheno Gene2) were assessed when each gene was targeted individually and also for all possible pair-wise combinations of genes. Percentages represent penetrance of phenotypes.

**Table 2:** Combinatorial RNAi can identify known synthetic lethal interactions

Strain	Interaction Gene1 + Gene 2	Gene1		Gene2		Gene1 + 2		Syn	*p *value	
				
		BS	ES	BS	ES	BS	ES		BS	ES
Wild-type	*mec-8 + sym-1*	88	99	82	98	78	92	Yes	5.5 × 10^-01^	1.3 × 10^-02^
	*sop-3 + sop-1*	91	100	94	99	79	90	Yes	2.8 × 10^-01^	8.5 × 10^-04^
	*tbx-8 + tbx-9*	83	99	78	97	52	11	Yes	7.3 × 10^-02^	1.4 × 10^-24^
	*hlh-1 + unc-120*	91	99	76	99	28	91	Yes	5.2 × 10^-05^	1.2 × 10^-02^
	*hlh-1 + hnd-1*	88	97	75	98	62	81	Yes	6.6 × 10^-01^	5.7 × 10^-03^
	*unc-120 + hnd-1*	54	100	74	98	36	100	No	6.4 × 10^-01^	1.9 × 10^-01^
	*egl-27 + egr-1*	93	99	79	90	90	89	No	6.0 × 10^-02^	7.4 × 10^-01^
*rrf-3*	*mec-8 + sym-1*	67	73	61	73	59	16	Yes	3.3 × 10^-01^	3.0 × 10^-06^
	*sop-3 + sop-1*	82	100	85	96	41	75	Yes	3.1 × 10^-04^	5.7 × 10^-06^
	*tbx-8 + tbx-9*	96	99	86	92	59	2	Yes	8.6 × 10^-03^	6.3 × 10^-27^
	*hlh-1 + unc-120*	90	90	31	99	1	64	Yes	8.1 × 10^-06^	2.9 × 10^-03^
	*hlh-1 + hnd-1*	86	87	82	94	42	24	Yes	1.6 × 10^-03^	8.2 × 10^-14^
	*unc-120 + hnd-1*	33	100	87	94	7	98	Yes	5.7 × 10^-04^	4.8 × 10^-02^
	*egl-27 + egr-1*	97	99	83	93	73	62	Yes	2.9 × 10^-01^	5.7 × 10^-08^

Quantitative analysis of known synthetic lethal interactions (Interaction Gene1 + Gene2; see below for references) after combinatorial RNA interference (RNAi) in wild-type or RNAi-hypersensitive *rrf-3 *worms [19]. Percentages of average wild-type brood size (BS) and embryonic survival (ES) rates resulting from RNAi targeting each gene individually (Gene1 or Gene2) as well as targeting both genes simultaneously (Gene1 + 2) are shown. A synthetic interaction (Syn) was scored positive for *p *< 5.0 × 10^-02 ^(by Student's t-test). References for genes tested: *mec-8 + sym-1 *[21]; *sop-3 + sop-1 *[22]; *tbx-8 + tbx-9 *[23]; *hlh-1 + unc-120*, *hlh-1 + hnd-1*, *unc-120 + hnd-1 *[24]; and *egl-27 + egr-1 *[25].

**Table 3:** Genetic interactions of synthetic multivulval genes can be recapitulated by combinatorial RNAi

SynMuv gene	Predicted gene	Locus	SynMuv pathway	Wild-type	*rrf-3*
*lin-15B*	T27C4.4	*egr-1*	A	-	-
	ZK678.1	*lin-15A*	A	Muv	Muv
	K12C11.2	*smo-1*	A, B	ns	ns
	W02A11.4	*uba-2*	A, B	Muv	Muv
*lin-15A*	K12C11.2	*smo-1*	A, B	ns	ns
	W02A11.4	*uba-2*	A, B	-	Muv
	C32F10.2	*lin-35*	B	Muv	Muv
	C47D12.1	*trr-1*	B	ns	ns
	C53A5.3	*hda-1/gon-10*	B	ns	ns
	E01A2.4		B	-	-
	F44B9.6	*lin-36*	B	-	Muv
	JC8.6		B	ns	ns
	K07A1.12	*lin-53/rba-2*	B	ns	ns
	M04B2.1	*mep-1/gei-2*	B	-	Muv
	R05D3.11	*met-2*	B	-	Muv
	R06C7.7	*rls-1/lin-61*	B	Muv	Muv
	W01G7.3		B	ns	ns
	W07B3.2	*gei-4*	B	ns	ns
	Y71G12B.9		B	-	Muv
	Y1O2A5C.18	*efl-1*	B	Muv	Muv
	ZK632.13	*lin-52*	B	Muv	Muv
	ZK637.7	*lin-9*	B	Muv	Muv
	ZK662.4	*lin-15B*	B	Muv	Muv

Previously studied synthetic multivulval (synMuv) genes were targeted by combinatorial RNA interference (RNAi) in wild-type or RNAi-hypersensitive *rrf-3 *worms [19]. We show predicted gene name, its corresponding genetic locus name, a definition of the gene as a component of either the synMuv A (A), synMuv B (B), or both (A, B) pathways. All synMuv A genes were targeted by RNAi in combination with a double-stranded (ds)RNA-expressing strain targeting the synMuv B gene *lin-15B*; corresponding experiments were performed with synMuv B genes and a dsRNA-expressing strain targeting *lin-15A*. In both cases, worms were scored for the presence of the multivulva (Muv) phenotype. -, absence of Muv phenotype; ns, not scored (RNAi resulted in embryonic lethality or sterility).

**Table 4:** *C. elegans *duplicate gene pairs with at least partially redundant functions

Interaction Gene1 + Gene2	Gene1		Gene2		Gene1 + 2		*p *value	
	
	BS	ES	BS	ES	BS	ES	BS	ES
*pab-1 *+ *pab-2*	15	100	88	100	0	ns	1.9 × 10^-04^	ns
*rpl-25.2 *+ *rpl-25.1*	6	50	17	63	0	ns	3.6 × 10^-04^	ns
*ptr-2 *+ *ptr-10*	^a^	53	^a^	98	^a^	ns	^a^	ns
*unc-78 *+ *tag-216*	85	96	98	97	0	ns	6.4 × 10^-15^	ns
*rab-8 *+ *rab-10*	87	98	70	96	1	ns	7.3 × 10^-05^	ns
B0495.2 + ZC504.3	84	99	97	99	2	55	6.3 × 10^-09^	2.9 × 10^-03^
*rpa-2 *+ C37A2.7	67	74	50	81	1	ns	1.9 × 10^-07^	ns
C28H8.4 + *erd-2*	93	95	86	94	10	10	5.6 × 10^-08^	2.2 × 10^-15^
*lin-12 *+ *glp-1*	90	88	99	83	16	75	1.2 × 10^-13^	7.3 × 10^-01^
C13G3.3 + W08G11.4	73	94	80	97	17	89	1.6 × 10^-06^	3.5 × 10^-01^
*lin-53 *+ *rba-1*	74	63	48	5	16	75	1.1 × 10^-02^	7.3 × 10^-17^
Y53C12A.4 + R02E12.2	84	81	78	87	32	75	1.3 × 10^-03^	6.9 × 10^-01^
F37C12.7 + *acs-17*	95	100	77	98	44	73	9.4 × 10^-03^	4.2 × 10^-06^
C05G5.4 + F23H11.3	96	100	94	98	58	72	5.1 × 10^-06^	1.5 × 10^-08^
*elt-6 *+ *egl-18*	100	97	82	88	63	73	4.0 × 10^-02^	6.3 × 10^-03^
*dsh-1 *+ *dsh-2*	97	98	75	54	58	17	1.6 × 10^-02^	1.1 × 10^-11^

*C. elegans *duplicate gene pairs (Interaction Gene1 + Gene2) displaying synthetic phenotypic effects upon combinatorial RNA interference (RNAi) in the RNAi-hypersensitive strain *rrf-3 *[19] are listed. Numbers shown are percentages of average wild-type brood size (BS) and embryonic survival (ES) rates for each gene individually (Gene1 or Gene2) as well as for duplicate gene pairs (Gene1 + 2), and are the arithmetic mean of two independent biological repeats. *p *values were assigned using a Student's t-test. ^a^Note that combinatorial RNAi against the duplicate gene pair *ptr-2 *+ *ptr-10 *resulted in an increased number of first generation larval growth arrested worms, rather than in reduced brood size; fraction of population which is wild-type (does not arrest at an early larval stage): 70% (*ptr-2*), 100% (*ptr-10*), 0% (*ptr-2 *+ *ptr-10*), *p *= 7.4 × 10^-09^. ns, given phenotype could not be scored.

**Table 5:** Molecular functions of *C. elegans *duplicate gene pairs with synthetic phenotypes

Duplicate gene pair	NCBI KOGs
*pab-1 *+ *pab-2*	Polyadenylate-binding protein (RRM superfamily)
*rpl-25.2 *+ *rpl-25.1*	60s ribosomal protein L23
*ptr-2 *+ *ptr-10*	Predicted membrane protein (patched superfamily)
*unc-78 *+ *tag-216*	WD40 repeat stress protein/actin interacting protein
*rab-8 *+ *rab-10*	GTP-binding protein SEC4, small G protein superfamily, and related Ras family GTP-binding proteins
B0495.2 + ZC504.3	Protein kinase PITSLRE and related kinases
*rpa-2 *+ C37A2.7	60S acidic ribosomal protein P2
C28H8.4 + *erd-2*	ER lumen protein retaining receptor
*lin-12 *+ *glp-1*	Member of the Notch/LIN-12/glp-1 transmembrane receptor family^a^
C13G3.3 + W08G11.4	Serine/threonine protein phosphatase 2A, regulatory subunit
*lin-53 *+ *rba-1*	Nucleosome remodeling factor, subunit CAF1/NURF55/MSI1
Y53C12A.4 + R02E12.2	Conserved protein Mo25
F37C12.7 + *acs-17*	Acyl-CoA synthetase
C05G5.4 + F23H11.3	Succinyl-CoA synthetase, alpha subunit
*elt-6 *+ *egl-18*	GATA-4/5/6 transcription factors
*dsh-1 *+ *dsh-2*	Dishevelled 3 and related proteins

NCBI eukaryotic orthologous groups (KOGs) [37] are listed for duplicate gene pairs with synthetic phenotypic effects upon combinatorial RNA interference (RNAi). ^a^Note that WormBase gene descriptions are used for the duplicate gene pair *lin-12 *+ *glp-1*.

## References

[B1] Giaever G, Chu AM, Ni L, Connelly C, Riles L, Veronneau S, Dow S, Lucau-Danila A, Anderson K, Andre B (2002). Functional profiling of the *Saccharomyces cerevisiae *genome.. Nature.

[B2] Kamath RS, Fraser AG, Dong Y, Poulin G, Durbin R, Gotta M, Kanapin A, Le Bot N, Moreno S, Sohrmann M (2003). Systematic functional analysis of the *Caenorhabditis elegans *genome using RNAi.. Nature.

[B3] Boutros M, Kiger AA, Armknecht S, Kerr K, Hild M, Koch B, Haas SA, Consortium HF, Paro R, Perrimon N (2004). Genome-wide RNAi analysis of growth and viability in *Drosophila *cells.. Science.

[B4] Hartman JL, Garvik B, Hartwell L (2001). Principles for the buffering of genetic variation.. Science.

[B5] Davierwala AP, Haynes J, Li Z, Brost RL, Robinson MD, Yu L, Mnaimneh S, Ding H, Zhu H, Chen Y (2005). The synthetic genetic interaction spectrum of essential genes.. Nat Genet.

[B6] Tong AH, Lesage G, Bader GD, Ding H, Xu H, Xin X, Young J, Berriz GF, Brost RL, Chang M (2004). Global mapping of the yeast genetic interaction network.. Science.

[B7] Lynch M, Force A (2000). The probability of duplicate gene preservation by subfunctionalization.. Genetics.

[B8] Force A, Lynch M, Pickett FB, Amores A, Yan YL, Postlethwait J (1999). Preservation of duplicate genes by complementary, degenerative mutations.. Genetics.

[B9] Wagner A (2002). Selection and gene duplication: a view from the genome.. Genome Biol.

[B10] Kondrashov FA, Rogozin IB, Wolf YI, Koonin EV (2002). Selection in the evolution of gene duplications.. Genome Biol.

[B11] Lynch M, Conery JS (2000). The evolutionary fate and consequences of duplicate genes.. Science.

[B12] Nowak MA, Boerlijst MC, Cooke J, Smith JM (1997). Evolution of genetic redundancy.. Nature.

[B13] Wagner A (2000). The role of population size, pleiotropy and fitness effects of mutations in the evolution of overlapping gene functions.. Genetics.

[B14] Conant GC, Wagner A (2004). Duplicate genes and robustness to transient gene knock-downs in *Caenorhabditis elegans*.. Proc Biol Sci.

[B15] Gu Z, Steinmetz LM, Gu X, Scharfe C, Davis RW, Li WH (2003). Role of duplicate genes in genetic robustness against null mutations.. Nature.

[B16] Castillo-Davis CI, Hartl DL (2002). Genome evolution and developmental constraint in *Caenorhabditis elegans*.. Mol Biol Evol.

[B17] Lehner B, Calixto A, Crombie C, Tischler J, Fortunato A, Chalfie M, Fraser AG (2006). Loss of LIN-35, the *Caenorhabditis elegans *ortholog of the tumor suppressor p105Rb, results in enhanced RNA interference.. Genome Biol.

[B18] Lehner B, Crombie C, Tischler J, Fortunato A, Fraser AG (2006). Systematic mapping of genetic interactions in *Caenorhabditis elegans *identifies common modifiers of diverse signaling pathways.. Nat Genet.

[B19] Simmer F, Tijsterman M, Parrish S, Koushika SP, Nonet ML, Fire A, Ahringer J, Plasterk RH (2002). Loss of the putative RNA-directed RNA polymerase RRF-3 makes *C. elegans *hypersensitive to RNAi.. Curr Biol.

[B20] Brenner S (1974). The genetics of *Caenorhabditis elegans*.. Genetics.

[B21] Davies AG, Spike CA, Shaw JE, Herman RK (1999). Functional overlap between the mec-8 gene and five sym genes in *Caenorhabditis elegans*.. Genetics.

[B22] Zhang H, Emmons SW (2001). The novel *C. elegans *gene sop-3 modulates Wnt signaling to regulate Hox gene expression.. Development.

[B23] Pocock R, Ahringer J, Mitsch M, Maxwell S, Woollard A (2004). A regulatory network of T-box genes and the even-skipped homologue vab-7 controls patterning and morphogenesis in *C. elegans*.. Development.

[B24] Baugh LR, Wen JC, Hill AA, Slonim DK, Brown EL, Hunter CP (2005). Synthetic lethal analysis of *Caenorhabditis elegans *posterior embryonic patterning genes identifies conserved genetic interactions.. Genome Biol.

[B25] Solari F, Bateman A, Ahringer J (1999). The *Caenorhabditis elegans *genes egl-27 and egr-1 are similar to MTA1, a member of a chromatin regulatory complex, and are redundantly required for embryonic patterning.. Development.

[B26] Ferguson EL, Horvitz HR (1989). The multivulva phenotype of certain *Caenorhabditis elegans *mutants results from defects in two functionally redundant pathways.. Genetics.

[B27] Poulin G, Dong Y, Fraser AG, Hopper NA, Ahringer J (2005). Chromatin regulation and sumoylation in the inhibition of Ras-induced vulval development in *Caenorhabditis elegans*.. EMBO J.

[B28] Poulin G, Dong Y, Fraser AG, Hopper NA, Ahringer J (2006). Chromatin regulation and sumoylation in the inhibition of Ras-induced vulval development in *C. elegans*.. EMBO J.

[B29] Gonczy P, Echeverri C, Oegema K, Coulson A, Jones SJ, Copley RR, Duperon J, Oegema J, Brehm M, Cassin E (2000). Functional genomic analysis of cell division in *C. elegans *using RNAi of genes on chromosome III.. Nature.

[B30] Parrish S, Fleenor J, Xu S, Mello C, Fire A (2000). Functional anatomy of a dsRNA trigger: differential requirement for the two trigger strands in RNA interference.. Mol Cell.

[B31] Remm M, Storm CE, Sonnhammer EL (2001). Automatic clustering of orthologs and in-paralogs from pairwise species comparisons.. J Mol Biol.

[B32] Drees BL, Thorsson V, Carter GW, Rives AW, Raymond MZ, Avila-Campillo I, Shannon P, Galitski T (2005). Derivation of genetic interaction networks from quantitative phenotype data.. Genome Biol.

[B33] Koh K, Peyrot SM, Wood CG, Wagmaister JA, Maduro MF, Eisenmann DM, Rothman JH (2002). Cell fates and fusion in the *C. elegans *vulval primordium are regulated by the EGL-18 and ELT-6 GATA factors - apparent direct targets of the LIN-39 Hox protein.. Development.

[B34] Lambie EJ, Kimble J (1991). Two homologous regulatory genes, lin-12 and glp-1, have overlapping functions.. Development.

[B35] Stein LD, Bao Z, Blasiar D, Blumenthal T, Brent MR, Chen N, Chinwalla A, Clarke L, Clee C, Coghlan A (2003). The genome sequence of *Caenorhabditis briggsae*: a platform for comparative genomics.. PLoS Biol.

[B36] Thompson JD, Higgins DG, Gibson TJ (1994). CLUSTAL W: improving the sensitivity of progressive multiple sequence alignment through sequence weighting, position-specific gap penalties and weight matrix choice.. Nucleic Acids Res.

[B37] Koonin EV, Fedorova ND, Jackson JD, Jacobs AR, Krylov DM, Makarova KS, Mazumder R, Mekhedov SL, Nikolskaya AN, Rao BS (2004). A comprehensive evolutionary classification of proteins encoded in complete eukaryotic genomes.. Genome Biol.

