# Supplementary material for: Combinatorial RNA interference in Caenorhabditis elegans reveals that redundancy between gene duplicates can be maintained for more than 80 million years of evolution
Source: Genome Biol. 2006 Aug 2;7(8):R69. doi: 10.1186/gb-2006-7-8-r69 (PMC1779603; doi:10.1186/gb-2006-7-8-r69)
Supplement: Additional data file 1 — A Word document listing C. elegans chromosome III genes with a previously assigned nonviable (embryonic lethal or sterile) RNAi phenotype [2] and the effect of dilution following combinatorial RNAi. [file gb-2006-7-8-r69-S1.doc]

*C. elegans* chromosome III genes with nonviable phenotypes [2]. Shown are targeting strains (‘GPName’), nonviable RNAi phenotypes (‘Emb’, ‘Ste’, ‘Lga’) observed for *rrf-3*, the n-fold dilution of targeting bacteria which first results in a weaker detectable phenotype of nonviable genes (‘Weaker phenotype’) and the n-fold dilution that results in the loss of any detectable phenotype (‘No phenotype’). Emb, embryonic lethal. Ste, sterile. Lga, larval growth arrest. Strengths of phenotypes were encoded numerically ranging from 1 (weak), 2 (medium), to 3 (strong). 0, no observed phenotype or no observed dilution effect in a given category, respectively.

| **GPName** | **Emb** | **Ste** | **Lga** | **Weaker phenotype** | **No phenotype** |
| --- | --- | --- | --- | --- | --- |
| B0285.2 | 2 | 2 | 0 | 0 | 2-fold |
| B0303.15 | 0 | 2 | 0 | 0 | 2-fold |
| B0336.1 | 0 | 0 | 0 | 0 | 0 |
| B0336.10 | 0 | 0 | 1 | 10-fold | 0 |
| B0336.2 | 0 | 3 | 0 | 2-fold | 3-fold |
| B0336.6 | 0 | 2 | 0 | 2-fold | 4-fold |
| B0361.10 | 0 | 2 | 0 | 2-fold | 3-fold |
| B0393.1 | 0 | 0 | 1 | 5-fold | 0 |
| B0393.6 | 0 | 1 | 0 | 0 | 2-fold |
| B0412.4 | 0 | 0 | 1 | 2-fold | 0 |
| B0464.1 | 0 | 0 | 1 | 2-fold | 0 |
| B0464.7 | 0 | 2 | 0 | 0 | 3-fold |
| C02F5.1 | 0 | 3 | 0 | 4-fold | 0 |
| C02F5.9 | 0 | 0 | 1 | 3-fold | 0 |
| C03C10.3 | 0 | 3 | 0 | 3-fold | 0 |
| C05D11.10 | 0 | 0 | 0 | 0 | 0 |
| C05D11.11 | 0 | 0 | 0 | 0 | 0 |
| C05D11.12 | 2 | 0 | 0 | 2-fold | 3-fold |
| C05D11.2 | 0 | 0 | 0 | 0 | 0 |
| C05D11.3 | 0 | 0 | 0 | 0 | 0 |
| C06E1.10 | 0 | 2 | 0 | 2-fold | 5-fold |
| C07A9.2 | 2 | 2 | 0 | 3-fold | 5-fold |
| C07A9.3 | 2 | 2 | 0 | 0 | 3-fold |
| C07G2.3 | 0 | 0 | 1 | 3-fold | 0 |
| C07H6.5 | 0 | 2 | 0 | 5-fold | 10-fold |
| C08C3.4 | 0 | 0 | 0 | 0 | 0 |
| C13B9.3 | 0 | 0 | 1 | 10-fold | 0 |
| C14B9.4 | 0 | 3 | 0 | 2-fold | 0 |
| C14B9.7 | 0 | 0 | 1 | 5-fold | 0 |
| C16A3.4 | 0 | 2 | 0 | 0 | 5-fold |
| C16A3.5 | 0 | 2 | 0 | 0 | 3-fold |
| C16A3.6 | 0 | 3 | 0 | 2-fold | 4-fold |
| C16A3.9 | 0 | 0 | 1 | 2-fold | 10-fold |
| C16C10.6 | 3 | 2 | 0 | 2-fold | 10-fold |
| C23G10.3 | 0 | 0 | 1 | 3-fold | 0 |
| C23G10.4 | 0 | 0 | 1 | 0 | 0 |
| C23G10.8 | 0 | 3 | 0 | 10-fold | 0 |
| C23G10.9 | 0 | 3 | 0 | 2-fold | 4-fold |
| C24H11.7 | 0 | 0 | 1 | 2-fold | 5-fold |
| C26E6.4 | 0 | 0 | 1 | 2-fold | 0 |
| C26E6.6 | 0 | 2 | 0 | 0 | 2-fold |
| C26E6.8 | 0 | 0 | 0 | 0 | 0 |
| C27D11.1 | 0 | 0 | 1 | 3-fold | 0 |
| C27F2.7 | 0 | 0 | 0 | 0 | 0 |
| C28H8.6 | 2 | 2 | 0 | 2-fold | 4-fold |
| C29E4.2 | 0 | 3 | 0 | 3-fold | 0 |
| C29E4.8 | 0 | 2 | 0 | 3-fold | 10-fold |
| C29F9.7 | 0 | 3 | 0 | 3-fold | 10-fold |
| C30C11.1 | 0 | 2 | 0 | 0 | 2-fold |
| C30C11.2 | 0 | 0 | 1 | 3-fold | 0 |
| C30C11.4 | 0 | 2 | 0 | 3-fold | 4-fold |
| C32A3.1 | 2 | 2 | 0 | 2-fold | 4-fold |
| C34C12.8 | 0 | 2 | 0 | 3-fold | 4-fold |
| C34E10.1 | 0 | 0 | 0 | 0 | 0 |
| C34E10.2 | 0 | 0 | 0 | 0 | 0 |
| C34E10.6 | 0 | 0 | 1 | 4-fold | 0 |
| C35D10.1 | 0 | 0 | 0 | 0 | 0 |
| C35D10.13 | 0 | 0 | 0 | 0 | 0 |
| C35D10.5 | 0 | 0 | 0 | 0 | 0 |
| C36A4.4 | 0 | 0 | 0 | 0 | 0 |
| C36E8.5 | 0 | 3 | 0 | 3-fold | 0 |
| C37G2.7 | 2 | 2 | 0 | 2-fold | 3-fold |
| C38C10.4 | 0 | 2 | 0 | 2-fold | 3-fold |
| C38D4.3 | 1 | 2 | 0 | 2-fold | 3-fold |
| C38D4.6 | 3 | 0 | 0 | 2-fold | 0 |
| C45G9.5 | 0 | 2 | 0 | 2-fold | 4-fold |
| C54C6.1 | 0 | 0 | 1 | 2-fold | 0 |
| C56G2.2 | 0 | 0 | 0 | 0 | 0 |
| C56G2.6 | 0 | 0 | 1 | 5-fold | 0 |
| D2007.4 | 0 | 0 | 0 | 0 | 0 |
| D2045.1 | 0 | 2 | 0 | 0 | 2-fold |
| D2045.6 | 1 | 2 | 0 | 0 | 3-fold |
| E03A3.3 | 0 | 3 | 0 | 2-fold | 10-fold |
| F01F1.12 | 0 | 0 | 0 | 0 | 0 |
| F01F1.7 | 2 | 2 | 0 | 2-fold | 4-fold |
| F01F1.8 | 0 | 0 | 1 | 2-fold | 10-fold |
| F02A9.4 | 0 | 0 | 0 | 0 | 0 |
| F02A9.6 | 2 | 2 | 0 | 2-fold | 3-fold |
| F08F8.2 | 0 | 2 | 0 | 0 | 2-fold |
| F09F7.3 | 0 | 3 | 0 | 2-fold | 10-fold |
| F09G8.3 | 0 | 2 | 0 | 0 | 3-fold |
| F10C5.1 | 0 | 2 | 0 | 2-fold | 4-fold |
| F10C5.2 | 0 | 2 | 0 | 4-fold | 5-fold |
| F10E9.7 | 0 | 0 | 1 | 2-fold | 10-fold |
| F10E9.8 | 2 | 2 | 0 | 2-fold | 3-fold |
| F11H8.4 | 0 | 2 | 0 | 0 | 3-fold |
| F13B10.2 | 0 | 0 | 1 | 5-fold | 0 |
| F20H11.3 | 0 | 0 | 0 | 0 | 0 |
| F21H11.4 | 0 | 0 | 0 | 0 | 0 |
| F22B7.5 | 0 | 2 | 0 | 0 | 2-fold |
| F23F12.2 | 0 | 0 | 0 | 0 | 0 |
| F23F12.6 | 0 | 0 | 1 | 10-fold | 0 |
| F23H11.5 | 0 | 3 | 0 | 2-fold | 10-fold |
| F25B5.4 | 0 | 0 | 1 | 0 | 0 |
| F26F4.10 | 0 | 2 | 0 | 2-fold | 3-fold |
| F26F4.11 | 0 | 2 | 0 | 0 | 3-fold |
| F30H5.1 | 0 | 3 | 0 | 10-fold | 0 |
| F31E3.3 | 0 | 0 | 0 | 0 | 0 |
| F35G12.10 | 0 | 2 | 0 | 0 | 2-fold |
| F35G12.8 | 0 | 3 | 0 | 3-fold | 0 |
| F37A4.8 | 0 | 0 | 0 | 0 | 0 |
| F37C12.1 | 2 | 2 | 0 | 2-fold | 3-fold |
| F37C12.11 | 0 | 0 | 1 | 3-fold | 0 |
| F37C12.13 | 2 | 2 | 0 | 2-fold | 4-fold |
| F37C12.3 | 0 | 0 | 0 | 0 | 0 |
| F37C12.4 | 0 | 3 | 0 | 0 | 2-fold |
| F37C12.9 | 0 | 0 | 1 | 5-fold | 0 |
| F43C1.2 | 0 | 2 | 0 | 2-fold | 3-fold |
| F43C1.5 | 0 | 0 | 0 | 0 | 0 |
| F43D9.3 | 0 | 0 | 1 | 4-fold | 0 |
| F44B9.7 | 0 | 0 | 0 | 0 | 0 |
| F48E8.2 | 0 | 0 | 0 | 0 | 0 |
| F48E8.5 | 0 | 0 | 0 | 0 | 0 |
| F53A2.4 | 0 | 2 | 0 | 2-fold | 3-fold |
| F53A3.3 | 0 | 0 | 1 | 2-fold | 0 |
| F54C4.1 | 0 | 0 | 0 | 0 | 0 |
| F54C8.2 | 2 | 2 | 0 | 2-fold | 3-fold |
| F54C8.3 | 0 | 2 | 0 | 0 | 2-fold |
| F54E7.2 | 0 | 3 | 0 | 2-fold | 3-fold |
| F54E7.3 | 3 | 2 | 0 | 2-fold | 0 |
| F54E7.4 | 0 | 0 | 0 | 0 | 0 |
| F54H12.1 | 0 | 2 | 0 | 2-fold | 5-fold |
| F54H12.6 | 0 | 2 | 0 | 3-fold | 4-fold |
| F56A8.6 | 0 | 3 | 0 | 2-fold | 10-fold |
| F56D2.1 | 0 | 3 | 0 | 2-fold | 5-fold |
| F56D2.6 | 0 | 3 | 0 | 2-fold | 3-fold |
| F56F3.2 | 2 | 2 | 0 | 0 | 2-fold |
| F56F3.5 | 0 | 0 | 1 | 10-fold | 0 |
| F57B9.10 | 0 | 0 | 1 | 10-fold | 0 |
| F57B9.2 | 0 | 0 | 1 | 4-fold | 0 |
| F57B9.3 | 0 | 0 | 1 | 2-fold | 5-fold |
| F57B9.5 | 0 | 3 | 0 | 2-fold | 4-fold |
| F57B9.6 | 0 | 0 | 1 | 3-fold | 10-fold |
| F58A4.3 | 3 | 2 | 0 | 3-fold | 10-fold |
| F58A4.4 | 0 | 3 | 0 | 3-fold | 0 |
| F58A4.8 | 0 | 2 | 0 | 0 | 2-fold |
| F58B6.3 | 2 | 2 | 0 | 2-fold | 0 |
| F59A2.1 | 0 | 3 | 0 | 2-fold | 10-fold |
| H06I04.a | 0 | 3 | 0 | 5-fold | 0 |
| H06I04.f | 0 | 0 | 1 | 2-fold | 0 |
| H14A12.2 | 0 | 0 | 0 | 0 | 0 |
| H19M22.1 | 0 | 0 | 1 | 2-fold | 0 |
| H19M22.3 | 0 | 3 | 0 | 2-fold | 0 |
| H38K22.2 | 0 | 0 | 0 | 0 | 0 |
| K01G5.1 | 0 | 3 | 0 | 2-fold | 3-fold |
| K01G5.4 | 0 | 0 | 1 | 4-fold | 0 |
| K01G5.7 | 0 | 3 | 0 | 2-fold | 5-fold |
| K02D10.5 | 0 | 0 | 1 | 4-fold | 10-fold |
| K03H1.2 | 2 | 2 | 0 | 2-fold | 3-fold |
| K04G7.1 | 0 | 2 | 0 | 3-fold | 4-fold |
| K04G7.4 | 0 | 2 | 0 | 0 | 10-fold |
| K04H4.1 | 0 | 2 | 0 | 2-fold | 3-fold |
| K06H7.1 | 0 | 3 | 0 | 2-fold | 0 |
| K06H7.6 | 0 | 2 | 0 | 2-fold | 3-fold |
| K08E3.5 | 2 | 2 | 0 | 2-fold | 4-fold |
| K08E3.6 | 0 | 3 | 0 | 3-fold | 0 |
| K10D2.4 | 0 | 0 | 0 | 0 | 0 |
| K10D2.6 | 2 | 2 | 0 | 2-fold | 5-fold |
| K11D9.1 | 0 | 0 | 0 | 0 | 0 |
| K11D9.2 | 0 | 0 | 1 | 2-fold | 0 |
| K12H4.3 | 0 | 3 | 0 | 2-fold | 3-fold |
| K12H4.4 | 0 | 2 | 0 | 2-fold | 3-fold |
| K12H4.5 | 0 | 2 | 0 | 2-fold | 3-fold |
| M01F1.3 | 0 | 3 | 0 | 2-fold | 5-fold |
| M03C11.7 | 2 | 2 | 0 | 2-fold | 3-fold |
| M88.2 | 0 | 0 | 0 | 0 | 0 |
| R01H10.1 | 1 | 1 | 0 | 0 | 4-fold |
| R07E5.10 | 0 | 2 | 0 | 2-fold | 4-fold |
| R07E5.14 | 0 | 3 | 0 | 2-fold | 10-fold |
| R07E5.3 | 0 | 3 | 0 | 2-fold | 10-fold |
| R08D7.1 | 2 | 2 | 0 | 2-fold | 4-fold |
| R08D7.2 | 0 | 2 | 0 | 0 | 2-fold |
| R08D7.3 | 0 | 3 | 0 | 2-fold | 0 |
| R107.6 | 0 | 0 | 0 | 0 | 0 |
| R10E11.1 | 0 | 0 | 1 | 10-fold | 0 |
| R10E11.2 | 0 | 0 | 1 | 3-fold | 0 |
| R10E11.8 | 0 | 0 | 1 | 0 | 0 |
| R10E4.4 | 0 | 3 | 0 | 2-fold | 10-fold |
| R12B2.4 | 0 | 2 | 0 | 2-fold | 3-fold |
| R12B2.5 | 0 | 0 | 1 | 2-fold | 0 |
| R13A5.12 | 0 | 3 | 0 | 3-fold | 0 |
| R13A5.13 | 0 | 3 | 0 | 3-fold | 0 |
| R13A5.8 | 0 | 0 | 1 | 5-fold | 0 |
| R13F6.1 | 2 | 2 | 0 | 2-fold | 3-fold |
| R13F6.10 | 0 | 0 | 0 | 0 | 0 |
| R144.2 | 0 | 0 | 1 | 2-fold | 10-fold |
| R144.3 | 0 | 1 | 0 | 0 | 2-fold |
| R144.7 | 0 | 0 | 0 | 0 | 0 |
| R151.3 | 0 | 0 | 1 | 2-fold | 0 |
| R151.9 | 0 | 0 | 0 | 0 | 0 |
| R74.1 | 0 | 3 | 0 | 2-fold | 10-fold |
| T03F6.5 | 0 | 1 | 0 | 0 | 2-fold |
| T04A8.11 | 0 | 2 | 0 | 0 | 2-fold |
| T04A8.6 | 0 | 3 | 0 | 2-fold | 5-fold |
| T04A8.7 | 1 | 1 | 0 | 3-fold | 5-fold |
| T05G5.10 | 0 | 0 | 0 | 0 | 0 |
| T05G5.3 | 0 | 3 | 0 | 2-fold | 0 |
| T07C4.7 | 0 | 0 | 0 | 0 | 0 |
| T08A11.2 | 0 | 0 | 1 | 2-fold | 10-fold |
| T10F2.1 | 0 | 0 | 1 | 2-fold | 0 |
| T10F2.4 | 3 | 2 | 0 | 2-fold | 10-fold |
| T12A2.2 | 0 | 0 | 1 | 2-fold | 10-fold |
| T12A2.7 | 0 | 0 | 0 | 0 | 0 |
| T12D8.1 | 2 | 2 | 0 | 2-fold | 4-fold |
| T12D8.6 | 3 | 2 | 0 | 3-fold | 0 |
| T12D8.7 | 0 | 0 | 0 | 0 | 0 |
| T16H12.4 | 0 | 1 | 0 | 0 | 2-fold |
| T17E9.1 | 0 | 0 | 0 | 0 | 0 |
| T17E9.2 | 0 | 2 | 0 | 0 | 2-fold |
| T17H7.5 | 0 | 0 | 1 | 2-fold | 10-fold |
| T20B12.1 | 0 | 2 | 0 | 0 | 5-fold |
| T20B12.2 | 0 | 2 | 0 | 0 | 3-fold |
| T20B12.7 | 0 | 0 | 0 | 0 | 0 |
| T20B12.8 | 0 | 2 | 0 | 0 | 5-fold |
| T20G5.1 | 0 | 0 | 1 | 0 | 0 |
| T20G5.2 | 0 | 3 | 0 | 2-fold | 10-fold |
| T20G5.3 | 0 | 0 | 1 | 0 | 0 |
| T20H4.3 | 0 | 0 | 1 | 4-fold | 0 |
| T20H4.5 | 0 | 2 | 0 | 0 | 2-fold |
| T23G5.1 | 0 | 3 | 0 | 3-fold | 0 |
| T24C4.5 | 0 | 3 | 0 | 2-fold | 0 |
| T25C8.2 | 0 | 0 | 1 | 0 | 0 |
| T26A5.3 | 0 | 2 | 0 | 0 | 3-fold |
| T26A5.9 | 2 | 2 | 0 | 2-fold | 10-fold |
| T26G10.1 | 0 | 3 | 0 | 2-fold | 10-fold |
| T27E9.1 | 0 | 2 | 0 | 0 | 10-fold |
| W04B5.4 | 0 | 0 | 0 | 0 | 0 |
| W06E11.1 | 0 | 2 | 0 | 4-fold | 10-fold |
| W06F12.1 | 0 | 3 | 0 | 2-fold | 10-fold |
| W07B3.2 | 0 | 3 | 0 | 10-fold | 0 |
| Y111B2C.e | 0 | 3 | 0 | 2-fold | 4-fold |
| Y111B2D.b | 0 | 0 | 1 | 0 | 0 |
| Y111B2D.h | 3 | 2 | 0 | 2-fold | 10-fold |
| Y119D3_444.b | 0 | 2 | 0 | 0 | 3-fold |
| Y119D3_446.a | 0 | 0 | 1 | 2-fold | 10-fold |
| Y119D3_446.c | 0 | 2 | 0 | 2-fold | 3-fold |
| Y37D8A.10 | 0 | 3 | 0 | 2-fold | 10-fold |
| Y37D8A.14 | 0 | 3 | 0 | 2-fold | 4-fold |
| Y37D8A.18 | 0 | 2 | 0 | 0 | 2-fold |
| Y39A1B.3 | 1 | 1 | 0 | 2-fold | 4-fold |
| Y39E4B.1 | 1 | 2 | 0 | 0 | 2-fold |
| Y41C4A.10 | 0 | 0 | 0 | 0 | 0 |
| Y42G9A.c | 0 | 0 | 0 | 0 | 0 |
| Y43F4B.6 | 1 | 2 | 0 | 2-fold | 3-fold |
| Y47D3A.c | 0 | 3 | 0 | 5-fold | 0 |
| Y47D3A.d | 0 | 3 | 0 | 2-fold | 10-fold |
| Y47D3B.7 | 0 | 0 | 1 | 2-fold | 0 |
| Y48A6B.11 | 0 | 0 | 0 | 0 | 0 |
| Y49E10.1 | 0 | 0 | 1 | 10-fold | 0 |
| Y49E10.14 | 2 | 2 | 0 | 10-fold | 0 |
| Y49E10.15 | 0 | 0 | 1 | 2-fold | 10-fold |
| Y49E10.19 | 2 | 2 | 0 | 10-fold | 0 |
| Y49E10.2 | 0 | 0 | 0 | 0 | 0 |
| Y49E10.21 | 0 | 2 | 0 | 2-fold | 3-fold |
| Y49E10.6 | 0 | 0 | 1 | 2-fold | 10-fold |
| Y53G8A_9248.c | 0 | 2 | 0 | 0 | 2-fold |
| Y53G8A_9248.d | 0 | 0 | 0 | 0 | 0 |
| Y53G8B_1025.a | 0 | 0 | 1 | 4-fold | 10-fold |
| Y53G8B_1025.b | 0 | 0 | 1 | 3-fold | 0 |
| Y55B1A_115.c | 0 | 1 | 0 | 0 | 3-fold |
| Y56A3A.32 | 0 | 0 | 0 | 0 | 0 |
| Y66A7A.8 | 2 | 2 | 0 | 2-fold | 4-fold |
| Y71D11A.b | 0 | 0 | 1 | 4-fold | 0 |
| Y71H2_375.b | 2 | 2 | 0 | 2-fold | 3-fold |
| Y71H2_378.a | 0 | 2 | 0 | 3-fold | 10-fold |
| Y71H2_388.c | 0 | 2 | 0 | 3-fold | 5-fold |
| Y71H2_388.d | 0 | 2 | 0 | 2-fold | 3-fold |
| Y71H2_389.e | 0 | 0 | 1 | 3-fold | 0 |
| Y75B8A.2 | 1 | 1 | 0 | 3-fold | 4-fold |
| Y76A2B.1 | 2 | 2 | 0 | 3-fold | 10-fold |
| ZK1010.1 | 0 | 0 | 1 | 10-fold | 0 |
| ZK1058.2 | 0 | 0 | 1 | 3-fold | 0 |
| ZK1098.7 | 0 | 0 | 0 | 0 | 0 |
| ZK1236.3 | 0 | 3 | 0 | 3-fold | 10-fold |
| ZK328.1 | 0 | 0 | 0 | 0 | 0 |
| ZK328.2 | 0 | 0 | 1 | 2-fold | 0 |
| ZK328.5 | 0 | 0 | 1 | 4-fold | 0 |
| ZK632.1 | 0 | 3 | 0 | 3-fold | 0 |
| ZK637.8 | 0 | 3 | 0 | 2-fold | 10-fold |
| ZK652.1 | 0 | 0 | 1 | 2-fold | 10-fold |
| ZK652.4 | 0 | 0 | 1 | 3-fold | 0 |
| ZK686.3 | 0 | 2 | 0 | 3-fold | 4-fold |
