# Supplementary material for: Combinatorial RNA interference in Caenorhabditis elegans reveals that redundancy between gene duplicates can be maintained for more than 80 million years of evolution
Source: Genome Biol. 2006 Aug 2;7(8):R69. doi: 10.1186/gb-2006-7-8-r69 (PMC1779603; doi:10.1186/gb-2006-7-8-r69)
Supplement: Additional data file 2 — A Word document listing C. elegans pairs of duplicated genes that have been screened for synthetic RNAi phenotypes. [file gb-2006-7-8-r69-S2.doc]

*C. elegans* duplicate gene pairs corresponding to single orthologs in *S. cerevisiae* and *D. melanogster* genomes. Shown are yeast and fly gene names (‘SC-ID/DM-ID’), whether the yeast gene is essential for viability, as defined by the gene deletion phenotype (‘SC essentiality’), *C. elegans* GenePairs names (‘GPName’), and Wormbase gene names (‘WBGeneName’). Lga, larval growth arrest (RNAi resulted in first generation larval growth arrest, rendering these genes and their corresponding duplicates not amenable to analysis).

| **SC-ID/DM-ID** | **SC essentiality** | **GPName** | **WBGeneName** | **Comments** |
| --- | --- | --- | --- | --- |
| S000000042/CG7758 | nonessential | D1025.2 | WBGene00008354 |  |
| S000000042/CG7758 | nonessential | F52A8.5 | WBGene00009918 |  |
| S000000071 | nonessential | F19H6.1 | WBGene00008956 |  |
| S000000071 | nonessential | Y39G10AR.3 | WBGene00021461 |  |
| S000000136/CG5183 | essential | C28H8.4 | WBGene00016195 |  |
| S000000136/CG5183 | essential | F09B9.3 | WBGene00001331 |  |
| S000000194 | nonessential | R07B7.5 | WBGene00011089 |  |
| S000000194 | nonessential | R07B7.4 | WBGene00011088 |  |
| S000000245 | nonessential | D1009.1 | WBGene00017012 |  |
| S000000245 | nonessential | F28D1.9 | WBGene00009218 |  |
| S000000313 | essential | T21H3.3 | WBGene00000552 | Lga |
| S000000313 | essential | C13C12.1 | WBGene00000285 |  |
| S000000331 | nonessential | F20B6.2 | WBGene00006921 | Lga |
| S000000331 | nonessential | Y110A7A.12 | WBGene00006921 |  |
| S000000339 | essential | Y71G12B.27 | WBGene00022162 |  |
| S000000339 | essential | C09G4.3 | WBGene00001051 |  |
| S000000426 | nonessential | C50H11.1 | WBGene00016849 |  |
| S000000426 | nonessential | F41C3.3 | WBGene00018269 |  |
| S000000730 | nonessential | C53B4.6 | WBGene00008275 |  |
| S000000730 | nonessential | F15B10.1 | WBGene00017480 |  |
| S000000782 | nonessential | K07A1.12 | WBGene00003036 |  |
| S000000782 | nonessential | K07A1.11 | WBGene00004312 |  |
| S000000807 | nonessential | R07E4.4 | WBGene00003254 |  |
| S000000807 | nonessential | C33H5.14 | WBGene00016380 |  |
| S000000967/CG5119 | essential | Y106G6H.2 | WBGene00003902 |  |
| S000000967/CG5119 | essential | F18H3.3 | WBGene00003903 |  |
| S000001156 | nonessential | F09E10.8 | WBGene00017298 |  |
| S000001156 | nonessential | K08E3.3 | WBGene00010663 |  |
| S000001219 | nonessential | C46H11.2 | WBGene00016728 |  |
| S000001219 | nonessential | C01H6.4 | WBGene00007254 |  |
| S000001285 | nonessential | H13N06.5 | WBGene00010398 |  |
| S000001285 | nonessential | T28F3.3 | WBGene00012129 |  |
| S000001324/CG9881 | essential | M01B12.3 | WBGene00000205 |  |
| S000001324/CG9881 | essential | C46H11.3 | WBGene00016729 |  |
| S000001327/CG17510 | nonessential | F13B9.8 | WBGene00001425 |  |
| S000001327/CG17510 | nonessential | F41G3.4 | WBGene00001424 |  |
| S000001417 | nonessential | T25G3.4 | WBGene00012031 |  |
| S000001417 | nonessential | Y50E8A.6 | WBGene00013049 |  |
| S000001448 | nonessential | K08D10.3 | WBGene00004386 |  |
| S000001448 | nonessential | K08D10.4 | WBGene00004385 |  |
| S000001458 | nonessential | K06A9.1 | WBGene00019435 |  |
| S000001458 | nonessential | H02F09.3 | WBGene00019146 |  |
| S000001501 | essential | C33H5.7 | WBGene00016374 |  |
| S000001501 | essential | C33H5.6 | WBGene00016373 |  |
| S000001507/CG6092 | essential | C29F7.3 | WBGene00007812 |  |
| S000001507/CG6092 | essential | F40F8.1 | WBGene00009575 |  |
| S000001672/CG4083 | essential | Y53C12A.4 | WBGene00013140 |  |
| S000001672/CG4083 | essential | R02E12.2 | WBGene00019827 |  |
| S000001676 | essential | T09A5.9 | WBGene00011637 |  |
| S000001676 | essential | C06A8.6 | WBGene00015516 |  |
| S000001701 | nonessential | Y51H7C.9 | WBGene00021787 |  |
| S000001701 | nonessential | K01C8.1 | WBGene00010456 |  |
| S000001873 | nonessential | F52C12.5 | WBGene00001253 |  |
| S000001873 | nonessential | F55A8.1 | WBGene00001186 |  |
| S000001877/CG1969 | essential | B0024.12 | WBGene00001646 |  |
| S000001877/CG1969 | essential | T23G11.2 | WBGene00001647 |  |
| S000001889 | essential | D1037.4 | WBGene00004272 |  |
| S000001889 | essential | T23H2.5 | WBGene00004273 |  |
| S000002161 | essential | K08A8.3 | WBGene00000591 |  |
| S000002161 | essential | F10G7.4 | WBGene00004737 |  |
| S000002195 | nonessential | C25G4.1 | WBGene00007729 |  |
| S000002195 | nonessential | C49C3.13 | WBGene00008203 |  |
| S000002210 | nonessential | T06E8.1 | WBGene00011543 |  |
| S000002210 | nonessential | F59F4.4 | WBGene00010339 |  |
| S000002255 | essential | F57B9.10 | WBGene00004462 | Lga |
| S000002255 | essential | F59B2.5 | WBGene00010309 |  |
| S000002290/CG1877 | essential | D2045.6 | WBGene00000836 |  |
| S000002290/CG1877 | essential | K08E7.7 | WBGene00000841 |  |
| S000002324 | nonessential | B0286.4 | WBGene00003825 |  |
| S000002324 | nonessential | F44A2.1 | WBGene00006499 |  |
| S000002389 | nonessential | K04D7.4 | WBGene00010558 |  |
| S000002389 | nonessential | C09D8.1 | WBGene00004215 |  |
| S000002424 | nonessential | Y22D7AR.6 | WBGene00021260 |  |
| S000002424 | nonessential | F30A10.3 | WBGene00009262 |  |
| S000002469 | essential | F43H9.2 | WBGene00018398 |  |
| S000002469 | essential | T22G5.5 | WBGene00011932 |  |
| S000002512/CG4672 | nonessential | Y57E12AL.1 | WBGene00021956 |  |
| S000002512/CG4672 | nonessential | R11H6.2 | WBGene00011250 |  |
| S000002571 | essential | F27D9.1 | WBGene00006757 |  |
| S000002571 | essential | T07A9.10 | WBGene00020298 |  |
| S000002584 | nonessential | F40G9.3 | WBGene00006715 |  |
| S000002584 | nonessential | C06E2.3 | WBGene00006716 |  |
| S000002727/CG10671 | nonessential | ZK265.5 | WBGene00013957 |  |
| S000002727/CG10671 | nonessential | F36D1.2 | WBGene00009462 |  |
| S000002781 | essential | C44C1.3 | WBGene00003563 |  |
| S000002781 | essential | K03E6.3 | WBGene00003565 |  |
| S000002808 | nonessential | F13H8.3 | WBGene00017436 |  |
| S000002808 | nonessential | Y43F8C.13 | WBGene00012834 |  |
| S000002818/CG11268 | nonessential | F21F3.3 | WBGene00017673 |  |
| S000002818/CG11268 | nonessential | M01E11.1 | WBGene00019710 |  |
| S000002819 | nonessential | R151.6 | WBGene00020109 |  |
| S000002819 | nonessential | F25D7.1 | WBGene00009111 |  |
| S000002885 | nonessential | T01C8.1 | WBGene00020142 |  |
| S000002885 | nonessential | PAR2.3 | WBGene00019801 |  |
| S000003292/CG1998 | essential | F49E12.9 | WBGene00009902 |  |
| S000003292/CG1998 | essential | F49E12.10 | WBGene00009903 |  |
| S000003596/CG6950 | nonessential | R03A10.4 | WBGene00010984 |  |
| S000003596/CG6950 | nonessential | F28H6.3 | WBGene00009232 |  |
| S000003691/CG3400 | nonessential | Y110A7A.6 | WBGene00022456 |  |
| S000003691/CG3400 | nonessential | K02B2.1 | WBGene00019295 |  |
| S000003723 | nonessential | F35H8.7 | WBGene00006938 |  |
| S000003723 | nonessential | Y53C12A.1 | WBGene00006940 |  |
| S000003865/CG11793 | nonessential | C15F1.7 | WBGene00004930 |  |
| S000003865/CG11793 | nonessential | ZK430.3 | WBGene00007036 |  |
| S000003887 | nonessential | R57.1 | WBGene00020082 |  |
| S000003887 | nonessential | C35C5.2 | WBGene00007954 |  |
| S000004017/CG4233 | nonessential | C44E4.3 | WBGene00016652 |  |
| S000004017/CG4233 | nonessential | C14F11.1 | WBGene00015778 |  |
| S000004252 | nonessential | F59B2.7 | WBGene00004269 |  |
| S000004252 | nonessential | T25G12.4 | WBGene00004270 |  |
| S000004372/CG32485 | nonessential | T03F7.7 | WBGene00011404 |  |
| S000004372/CG32485 | nonessential | C34C12.6 | WBGene00007925 |  |
| S000004524 | nonessential | ZK370.4 | WBGene00022718 |  |
| S000004524 | nonessential | M110.7 | WBGene00010915 |  |
| S000004640 | nonessential | B0280.4 | WBGene00003845 |  |
| S000004640 | nonessential | C34H3.2 | WBGene00003846 |  |
| S000004698/CG10724 | nonessential | C04F6.4 | WBGene00006810 |  |
| S000004698/CG10724 | nonessential | K08F9.2 | WBGene00010685 |  |
| S000004716/CG11140 | nonessential | T05H4.13 | WBGene00000110 |  |
| S000004716/CG11140 | nonessential | T08B1.3 | WBGene00000111 |  |
| S000004840/CG2670 | essential | F54F7.1 | WBGene00006388 |  |
| S000004840/CG2670 | essential | Y111B2A.16 | WBGene00006389 |  |
| S000004990/CG10602 | nonessential | C42C1.11 | WBGene00016589 |  |
| S000004990/CG10602 | nonessential | ZC416.6 | WBGene00022610 |  |
| S000005027/CG32103 | nonessential | F17E5.2 | WBGene00008924 |  |
| S000005027/CG32103 | nonessential | F55A11.4 | WBGene00010077 |  |
| S000005187/CG10971 | nonessential | ZK370.3 | WBGene00022717 |  |
| S000005187/CG10971 | nonessential | F08A8.6 | WBGene00006484 |  |
| S000005274 | nonessential | C53A5.3 | WBGene00001834 |  |
| S000005274 | nonessential | R06C1.1 | WBGene00001836 |  |
| S000005487/CG7977 | essential | F52B5.6 | WBGene00004439 |  |
| S000005487/CG7977 | essential | F55D10.2 | WBGene00004438 |  |
| S000005540 | nonessential | C13G3.3 | WBGene00007554 |  |
| S000005540 | nonessential | W08G11.4 | WBGene00012348 |  |
| S000005668/CG1065 | nonessential | C05G5.4 | WBGene00007350 |  |
| S000005668/CG1065 | nonessential | F23H11.3 | WBGene00017759 |  |
| S000005701/CG18445 | nonessential | C54G7.2 | WBGene00016934 |  |
| S000005701/CG18445 | nonessential | C08F8.4 | WBGene00007446 |  |
| S000005927 | nonessential | F02E8.6 | WBGene00003561 |  |
| S000005927 | nonessential | F09G8.4 | WBGene00003562 |  |
| S000006075 | nonessential | R12H7.2 | WBGene00000217 |  |
| S000006075 | nonessential | H22K11.1 | WBGene00000216 |  |
| S000006168 | nonessential | F53C11.7 | WBGene00009976 |  |
| S000006168 | nonessential | F53C11.8 | WBGene00009977 |  |
| S000006180 | nonessential | K11D2.3 | WBGene00006829 |  |
| S000006180 | nonessential | F55A12.7 | WBGene00000150 | Lga |
| S000006225 | nonessential | F55G1.5 | WBGene00018901 |  |
| S000006225 | nonessential | F20D1.9 | WBGene00008979 |  |
| S000006240 | nonessential | T14F9.1 | WBGene00020507 | Lga |
| S000006240 | nonessential | F52E1.10 | WBGene00018698 |  |
| S000006266 | nonessential | R13A5.10 | WBGene00020052 |  |
| S000006266 | nonessential | Y48A6B.7 | WBGene00012968 |  |
| S000006351/CG9186 | nonessential | F26A3.1 | WBGene00009140 |  |
| S000006351/CG9186 | nonessential | F11C1.4 | WBGene00008693 |  |
| S000006437/CG10071 | nonessential | B0513.3 | WBGene00004443 |  |
| S000006437/CG10071 | nonessential | Y47H9C.14 | WBGene00012956 |  |
| CG10211 |  | T06D8.10 | WBGene00011530 |  |
| CG10211 |  | C46A5.4 | WBGene00016700 |  |
| CG10512 |  | VF13D12L.3 | WBGene00012149 |  |
| CG10512 |  | F36A2.3 | WBGene00009453 |  |
| CG1086 |  | H17B01.1 | WBGene00019207 |  |
| CG1086 |  | R09B5.11 | WBGene00019979 |  |
| CG10992 |  | C25B8.3 | WBGene00000786 |  |
| CG10992 |  | F57F5.1 | WBGene00010204 | Lga |
| CG11049 |  | K06B9.5 | WBGene00003938 |  |
| CG11049 |  | C04G2.7 | WBGene00001204 |  |
| CG11212 |  | C32E8.8 | WBGene00004217 |  |
| CG11212 |  | F55F8.1 | WBGene00004224 |  |
| CG12002 |  | ZK994.3 | WBGene00004256 |  |
| CG12002 |  | K09C8.5 | WBGene00004257 |  |
| CG12070 |  | C28C12.7 | WBGene00004995 |  |
| CG12070 |  | C28C12.5 | WBGene00004993 |  |
| CG12265 |  | C50B8.2 | WBGene00000250 |  |
| CG12265 |  | T27F2.3 | WBGene00000249 |  |
| CG12428 |  | T20B3.1 | WBGene00011850 |  |
| CG12428 |  | F41E7.6 | WBGene00009622 |  |
| CG12512 |  | F46E10.1 | WBGene00018488 | Lga |
| CG12512 |  | F28F8.2 | WBGene00009221 |  |
| CG13431 |  | B0416.6 | WBGene00001638 |  |
| CG13431 |  | M01F1.1 | WBGene00001639 |  |
| CG13645 |  | F26H9.4 | WBGene00009176 |  |
| CG13645 |  | W06B3.1 | WBGene00012295 |  |
| CG13994 |  | ZK945.8 | WBGene00014170 |  |
| CG13994 |  | K10H10.7 | WBGene00010763 |  |
| CG1411 |  | C47E12.8 | WBGene00000964 |  |
| CG1411 |  | R06C7.3 | WBGene00000963 |  |
| CG14351 |  | C44H4.3 | WBGene00006366 |  |
| CG14351 |  | C44H4.2 | WBGene00006370 | Lga |
| CG14507 |  | C03H5.4 | WBGene00015406 |  |
| CG14507 |  | C07E3.9 | WBGene00007419 |  |
| CG1607 |  | F27C8.1 | WBGene00000002 |  |
| CG1607 |  | F52H2.2 | WBGene00000004 |  |
| CG16726 |  | F16C3.1 | WBGene00008885 |  |
| CG16726 |  | C24B5.1 | WBGene00016044 |  |
| CG16975 |  | R06C7.7 | WBGene00003041 |  |
| CG16975 |  | Y48G1A.6 | WBGene00021661 |  |
| CG16986 |  | C25H3.3 | WBGene00016112 |  |
| CG16986 |  | C25H3.14 | WBGene00016123 |  |
| CG1772 |  | T05A6.1 | WBGene00000516 |  |
| CG1772 |  | T05A6.2 | WBGene00000517 |  |
| CG1815 |  | F23H11.1 | WBGene00017757 |  |
| CG1815 |  | F54B11.6 | WBGene00010029 |  |
| CG18361 |  | C34F11.9 | WBGene00001101 |  |
| CG18361 |  | C27A2.6 | WBGene00001102 |  |
| CG1882 |  | C37H5.3 | WBGene00016507 |  |
| CG1882 |  | C37H5.2 | WBGene00016506 |  |
| CG2092 |  | K10B2.5 | WBGene00019608 |  |
| CG2092 |  | Y43F8C.14 | WBGene00012835 |  |
| CG2201 |  | C28D4.2 | WBGene00000509 |  |
| CG2201 |  | C52B9.1 | WBGene00000510 |  |
| CG2493 |  | ZK688.6 | WBGene00022801 |  |
| CG2493 |  | ZK112.1 | WBGene00003956 |  |
| CG2669 |  | C24H12.5 | WBGene00016074 |  |
| CG2669 |  | C24H12.2 | WBGene00016072 |  |
| CG2864 |  | H23L24.5 | WBGene00004052 |  |
| CG2864 |  | F20C5.1 | WBGene00004051 |  |
| CG31022 |  | F35G2.4 | WBGene00004025 |  |
| CG31022 |  | Y47D3B.10 | WBGene00001077 |  |
| CG31033 |  | K06A1.5 | WBGene00019427 |  |
| CG31033 |  | F02E8.5 | WBGene00017178 |  |
| CG3105 |  | F45H7.4 | WBGene00004183 |  |
| CG3105 |  | C06E8.3 | WBGene00004182 |  |
| CG3156 |  | F57A10.3 | WBGene00001813 |  |
| CG3156 |  | C30H6.6 | WBGene00001811 |  |
| CG31645 |  | T07D4.1 | WBGene00011578 |  |
| CG31645 |  | T23C6.5 | WBGene00020727 |  |
| CG3186 |  | T05G5.10 | WBGene00002064 |  |
| CG3186 |  | F54C9.1 | WBGene00002065 |  |
| CG3187 |  | F46G10.7 | WBGene00004801 |  |
| CG3187 |  | F46G10.3 | WBGene00004802 |  |
| CG3198 |  | C50D2.8 | WBGene00016811 |  |
| CG3198 |  | Y119D3B.12 | WBGene00022489 |  |
| CG32099 |  | T04G9.4 | WBGene00020215 |  |
| CG32099 |  | T28H10.1 | WBGene00012142 |  |
| CG3280 |  | T13G4.3 | WBGene00020490 |  |
| CG3280 |  | B0416.1 | WBGene00015177 |  |
| CG3456 |  | C10E2.6 | WBGene00015676 |  |
| CG3456 |  | T02G5.12 | WBGene00020168 |  |
| CG3752 |  | F54D8.3 | WBGene00000107 |  |
| CG3752 |  | K04F1.15 | WBGene00000108 |  |
| CG3887 |  | C35C5.3 | WBGene00007955 |  |
| CG3887 |  | F28H7.4 | WBGene00009238 |  |
| CG3902 |  | C55B7.4 | WBGene00016943 |  |
| CG3902 |  | K06A5.6 | WBGene00019433 |  |
| CG3936 |  | R107.8 | WBGene00003001 |  |
| CG3936 |  | F02A9.6 | WBGene00001609 |  |
| CG40080 |  | C01H6.9 | WBGene00007258 |  |
| CG40080 |  | Y18H1A.10 | WBGene00021214 |  |
| CG4239 |  | Y57A10A.28 | WBGene00013268 |  |
| CG4239 |  | Y57A10A.10 | WBGene00013255 |  |
| CG4268 |  | B0495.2 | WBGene00015203 |  |
| CG4268 |  | ZC504.3 | WBGene00013917 |  |
| CG4349 |  | D1037.3 | WBGene00001501 |  |
| CG4349 |  | C54F6.14 | WBGene00001500 |  |
| CG4918 |  | Y62E10A.1 | WBGene00004410 |  |
| CG4918 |  | C37A2.7 | WBGene00016493 |  |
| CG4965 |  | F16B4.8 | WBGene00000387 |  |
| CG4965 |  | ZK637.11 | WBGene00000388 |  |
| CG5014 |  | F33D11.11 | WBGene00018008 |  |
| CG5014 |  | F42G2.5 | WBGene00018354 |  |
| CG5036 |  | F16H9.1 | WBGene00004345 |  |
| CG5036 |  | C05B5.7 | WBGene00004344 |  |
| CG5695 |  | F47G6.4 | WBGene00004969 |  |
| CG5695 |  | Y66H1A.6 | WBGene00002041 |  |
| CG5805 |  | F13G3.7 | WBGene00008767 |  |
| CG5805 |  | Y43C5B.3 | WBGene00012786 |  |
| CG6016 |  | Y49A3A.1 | WBGene00013024 |  |
| CG6016 |  | F22E10.5 | WBGene00009057 |  |
| CG6214 |  | F57C12.5 | WBGene00003407 |  |
| CG6214 |  | F57C12.4 | WBGene00003408 |  |
| CG6455 |  | T14G11.3 | WBGene00020511 |  |
| CG6455 |  | W06H3.1 | WBGene00012315 |  |
| CG8032 |  | C24G6.6 | WBGene00016061 |  |
| CG8032 |  | F25C8.2 | WBGene00000139 |  |
| CG8055 |  | F21A10.2 | WBGene00008999 |  |
| CG8055 |  | F59B10.1 | WBGene00004134 | Lga |
| CG8055 |  | C56C10.3 | WBGene00016961 | Lga |
| CG8055 |  | C37C3.3 | WBGene00016497 | Lga |
| CG8057 |  | F55F3.1 | WBGene00010115 |  |
| CG8057 |  | Y47D3A.15 | WBGene00012928 |  |
| CG8245 |  | C09B8.4 | WBGene00015623 |  |
| CG8245 |  | T10B11.6 | WBGene00020402 |  |
| CG8286 |  | C55B6.2 | WBGene00001025 |  |
| CG8286 |  | Y54E10BL.4 | WBGene00001046 |  |
| CG8428 |  | Y111B2A.19 | WBGene00013739 |  |
| CG8428 |  | C13C4.5 | WBGene00007549 |  |
| CG8434 |  | T21D12.9 | WBGene00020649 |  |
| CG8434 |  | R13.3 | WBGene00011258 |  |
| CG8460 |  | C44C1.2 | WBGene00016642 |  |
| CG8460 |  | R09B5.12 | WBGene00019980 |  |
| CG8732 |  | F37C12.7 | WBGene00018152 |  |
| CG8732 |  | C46F4.2 | WBGene00016716 |  |
| CG9117 |  | F53B1.6 | WBGene00018738 |  |
| CG9117 |  | C03F11.2 | WBGene00015388 |  |
| CG9247 |  | ZK1098.3 | WBGene00014220 |  |
| CG9247 |  | ZK1098.8 | WBGene00003504 |  |
| CG9347 |  | F53C3.12 | WBGene00018755 |  |
| CG9347 |  | Y46G5A.24 | WBGene00012914 |  |
| CG9427 |  | F37H8.5 | WBGene00009514 |  |
| CG9427 |  | K07D4.8 | WBGene00004135 |  |
| CG9577 |  | Y25C1A.13 | WBGene00021296 |  |
| CG9577 |  | F58A6.1 | WBGene00019022 |  |
| CG9738 |  | F42G10.2 | WBGene00003368 |  |
| CG9738 |  | VZC374L.1 | WBGene00012162 |  |
| CG9755 |  | C30G12.7 | WBGene00004244 |  |
| CG9755 |  | W06B11.2 | WBGene00004245 |  |
