# Supplementary material for: Combinatorial RNA interference in Caenorhabditis elegans reveals that redundancy between gene duplicates can be maintained for more than 80 million years of evolution
Source: Genome Biol. 2006 Aug 2;7(8):R69. doi: 10.1186/gb-2006-7-8-r69 (PMC1779603; doi:10.1186/gb-2006-7-8-r69)
Supplement: Additional data file 3 — A Word document listing C. elegans 1:1 orthologs of S. cerevisiae genes and their RNAi phenotypes. [file gb-2006-7-8-r69-S3.doc]

*C. elegans* 1:1 orthologs of *S. cerevisiae* genes. *S. cerevisiae* genes (‘SC Gene’) and their phenotypes (‘SC essentiality’), their corresponding orthologs in *C. elegans* and whether they show nonviable (‘NonV’, i.e. embryonic lethal or sterile) phenotypes in two independent RNAi experiments (indicated by x) are listed. GPName, *C. elegans* GenePairs name. WBGeneName, Wormbase gene name.

| **SC Gene** | **SC essentiality** | **GPName** | **WBGeneName** | **NonV** |
| --- | --- | --- | --- | --- |
| 2AAA_YEAST | nonessential | F48E8.5 | WBGene00003901 |  |
| 2ABA_YEAST | nonessential | F26E4.1 | WBGene00006352 | x |
| 3HAO_YEAST | nonessential | K06A4.5 | WBGene00010595 |  |
| ABC1_YEAST | nonessential | C35D10.4 | WBGene00000767 |  |
| ABD1_YEAST | essential | C25A1.3 | WBGene00006447 |  |
| ABP1_YEAST | nonessential | K08E3.4 | WBGene00010664 |  |
| ABPX_YEAST | nonessential | ZK1058.5 | WBGene00014205 |  |
| ACBP_YEAST | nonessential | C44E4.6 | WBGene00016655 |  |
| ACON_YEAST | nonessential | F54H12.1 | WBGene00000041 | x |
| ADA_YEAST | nonessential | C06G3.5 | WBGene00015551 |  |
| ADA1_YEAST | nonessential | T20B5.1 | WBGene00000161 |  |
| ADA2_YEAST | nonessential | F32A5.1 | WBGene00017967 |  |
| ADB2_YEAST | nonessential | Y71H2_389.e | WBGene00000160 | x |
| ADB6_YEAST | nonessential | R11A5.1 | WBGene00000163 |  |
| ADK_YEAST | nonessential | R07H5.8 | WBGene00011128 |  |
| ADPP_YEAST | nonessential | W02G9.1 | WBGene00003579 |  |
| ADRO_YEAST | essential | Y62E10A.f | WBGene00013376 |  |
| AFG1_YEAST | nonessential | C30F12.2 | WBGene00016261 |  |
| AGM1_YEAST | essential | F21D5.1 | WBGene00009006 |  |
| ALG1_YEAST | essential | T26A5.4 | WBGene00020820 |  |
| ALG2_YEAST | essential | F09E5.2 | WBGene00017282 | x |
| ALG3_YEAST | nonessential | K09E4.2 | WBGene00010720 |  |
| ALG6_YEAST | nonessential | C08B11.8 | WBGene00007435 |  |
| ALG9_YEAST | nonessential | C14A4.3 | WBGene00007556 |  |
| AMDM_YEAST | nonessential | C34F11.3 | WBGene00016415 |  |
| AMP2_YEAST | nonessential | Y116A8A.9 | WBGene00003130 |  |
| AP10_YEAST | nonessential | F15H10.3 | WBGene00000144 |  |
| AP17_YEAST | nonessential | F02E8.3 | WBGene00000157 |  |
| AP19_YEAST | nonessential | F29G9.3 | WBGene00000159 |  |
| APG6_YEAST | nonessential | T19E7.3 | WBGene00000247 |  |
| APG6_YEAST | nonessential | T19E7.4 | WBGene00000247 |  |
| APG7_YEAST | nonessential | M7.5 | WBGene00010882 |  |
| APG8_YEAST | nonessential | C32D5.9 | WBGene00002980 |  |
| APN1_YEAST | nonessential | T05H10.2 | WBGene00000151 |  |
| APN2_YEAST | nonessential | R09B3.1 | WBGene00001372 |  |
| AR21_YEAST | nonessential | Y37D8A.1 | WBGene00000203 |  |
| AR41_YEAST | essential | Y79H2A.6 | WBGene00000201 |  |
| ARG1_YEAST | nonessential | D1081.2 | WBGene00006844 | x |
| ARGI_YEAST | nonessential | T21F4.1 | WBGene00020658 |  |
| ARL1_YEAST | nonessential | F54C9.10 | WBGene00000187 | x |
| ARP2_YEAST | essential | K07C5.1 | WBGene00000200 |  |
| ARP6_YEAST | nonessential | C08B11.6 | WBGene00007434 |  |
| ATC6_YEAST | nonessential | C10C6.6 | WBGene00007514 |  |
| ATPB_YEAST | nonessential | C34E10.6 | WBGene00000229 | x |
| ATPD_YEAST | nonessential | F58F12.1 | WBGene00019061 |  |
| ATPO_YEAST | nonessential | F27C1.7 | WBGene00017856 | x |
| ATU2_YEAST | nonessential | Y76A2A.3 | WBGene00000834 |  |
| ATU2_YEAST | nonessential | F45G2.11 | WBGene00000834 |  |
| ATU2_YEAST | nonessential | Y76A2A.2 | WBGene00000834 |  |
| ATX1_YEAST | nonessential | ZK652.2 | WBGene00000835 |  |
| ATX2_YEAST | nonessential | T01D3.5 | WBGene00011329 |  |
| AUT1_YEAST | nonessential | Y55F3A_746.a | WBGene00021922 |  |
| AUT1_YEAST | nonessential | Y55F3A_746.e | WBGene00021922 |  |
| BCS1_YEAST | nonessential | F54C9.6 | WBGene00010042 |  |
| BET2_YEAST | essential | B0280.11 | WBGene00015099 |  |
| BET3_YEAST | essential | ZK1098.5 | WBGene00014222 |  |
| BET4_YEAST | essential | M57.2 | WBGene00019778 |  |
| BMS1_YEAST | essential | Y61A9LA_74.b | WBGene00022021 |  |
| BMS1_YEAST | essential | Y61A9LA_75.a | WBGene00022021 | x |
| BPH1_YEAST | nonessential | VT23B5.2 | WBGene00012154 |  |
| BRX1_YEAST | essential | K12H4.3 | WBGene00019678 | x |
| BU31_YEAST | nonessential | C07A9.2 | WBGene00007400 | x |
| BUB2_YEAST | nonessential | C33F10.2 | WBGene00016352 |  |
| CAC2_YEAST | nonessential | Y71G12A_202.d | WBGene00022141 |  |
| CACM_YEAST | nonessential | R07H5.2 | WBGene00011122 |  |
| CACP_YEAST | nonessential | B0395.3 | WBGene00007175 |  |
| CALX_YEAST | nonessential | ZK632.6 | WBGene00000567 |  |
| CAP_YEAST | nonessential | F41G4.2 | WBGene00000294 |  |
| CAPA_YEAST | nonessential | D2024.6 | WBGene00000292 | x |
| CAPB_YEAST | nonessential | M106.5 | WBGene00000293 | x |
| CAT5_YEAST | nonessential | ZC395.2 | WBGene00000536 |  |
| CBF5_YEAST | essential | K01G5.5 | WBGene00010478 |  |
| CBP3_YEAST | nonessential | C35D10.5 | WBGene00016442 |  |
| CC16_YEAST | essential | F10B5.6 | WBGene00001281 | x |
| CC27_YEAST | essential | Y110A7A.d | WBGene00003132 | x |
| CC28_YEAST | essential | T05G5.3 | WBGene00000405 | x |
| CC42_YEAST | essential | R07G3.1 | WBGene00000390 | x |
| CC45_YEAST | essential | F34D10.2 | WBGene00009372 |  |
| CC45_YEAST | essential | F34D10.3 | WBGene00009372 |  |
| CC47_YEAST | essential | F32D1.10 | WBGene00003159 | x |
| CC54_YEAST | essential | Y39G10A_246.e | WBGene00003156 | x |
| CC68_YEAST | essential | F55A3.7 | WBGene00018849 |  |
| CCHL_YEAST | nonessential | T06D8.6 | WBGene00011527 |  |
| CCL1_YEAST | essential | Y49F6B.r | WBGene00021714 |  |
| CCR4_YEAST | nonessential | ZC518.3 | WBGene00000376 |  |
| CDC6_YEAST | essential | C43E11.10 | WBGene00000382 |  |
| CDS1_YEAST | essential | C33H5.18 | WBGene00016384 |  |
| CEF1_YEAST | essential | D1081.8 | WBGene00008386 | x |
| CEM1_YEAST | nonessential | F10G8.9 | WBGene00008667 |  |
| CG48_YEAST | nonessential | B0280.9 | WBGene00015104 | x |
| CH12_YEAST | nonessential | K08F4.1 | WBGene00010676 |  |
| CHD1_YEAST | nonessential | H06O01.2 | WBGene00010369 |  |
| CHL1_YEAST | nonessential | M03C11.2 | WBGene00010839 |  |
| CHS5_YEAST | nonessential | ZC449.5 | WBGene00022615 |  |
| CLH_YEAST | nonessential | T20G5.1 | WBGene00011867 | x |
| CND1_YEAST | essential | Y39A1B.3 | WBGene00001087 |  |
| CNS1_YEAST | essential | C17G10.2 | WBGene00015916 |  |
| COD1_YEAST | essential | Y51H7C_255.f | WBGene00021784 | x |
| COD1_YEAST | essential | Y51H7C_255.g | WBGene00021784 | x |
| COD1_YEAST | essential | Y51H7C_255.b | WBGene00021784 |  |
| COD1_YEAST | essential | Y51H7C_255.c | WBGene00021784 | x |
| COD2_YEAST | nonessential | K07C11.9 | WBGene00019481 |  |
| COPA_YEAST | essential | Y71F9A_282.b | WBGene00022119 | x |
| COPB_YEAST | essential | Y25C1A.5 | WBGene00021292 | x |
| COPD_YEAST | essential | C13B9.3 | WBGene00015734 | x |
| COPG_YEAST | essential | T14G10.5 | WBGene00011775 | x |
| COPP_YEAST | essential | F38E11.5 | WBGene00009542 | x |
| COPZ_YEAST | essential | F59E10.3 | WBGene00010333 | x |
| COQ1_YEAST | nonessential | C24A11.8 | WBGene00000761 |  |
| COQ1_YEAST | nonessential | C24A11.3 | WBGene00000761 |  |
| COQ1_YEAST | nonessential | C30H7.1 | WBGene00000761 |  |
| COQ3_YEAST | nonessential | Y57G11C.11 | WBGene00000763 |  |
| COQ4_YEAST | nonessential | T03F1.2 | WBGene00000764 |  |
| COQ6_YEAST | nonessential | K07B1.2 | WBGene00000766 |  |
| CORO_YEAST | nonessential | R01H10.3 | WBGene00000768 |  |
| COX6_YEAST | nonessential | Y37D8A.14 | WBGene00012553 | x |
| COXE_YEAST | nonessential | F54D8.2 | WBGene00018800 |  |
| COXG_YEAST | nonessential | Y71H2_388.c | WBGene00022170 | x |
| COXG_YEAST | nonessential | Y71H2_388.f | WBGene00022170 |  |
| COXW_YEAST | nonessential | T06D8.5 | WBGene00011526 |  |
| COXX_YEAST | nonessential | Y46G5.a | WBGene00012895 |  |
| COXZ_YEAST | nonessential | JC8.5 | WBGene00010437 |  |
| CRC1_YEAST | nonessential | F49E8.5 | WBGene00000996 |  |
| CRD1_YEAST | nonessential | F23H11.9 | WBGene00017763 |  |
| CSE1_YEAST | essential | Y48G1A_54.b | WBGene00002079 | x |
| CSE1_YEAST | essential | Y48G1A_54.c | WBGene00002079 | x |
| CTK1_YEAST | nonessential | B0285.1 | WBGene00007135 | x |
| CTK1_YEAST | nonessential | B0285.2 | WBGene00007135 | x |
| CW41_YEAST | nonessential | F13H10.4 | WBGene00008775 |  |
| CW43_YEAST | nonessential | T04A8.12 | WBGene00011413 |  |
| CY1_YEAST | nonessential | C54G4.8 | WBGene00000869 |  |
| CYAA_YEAST | essential | F43C1.1 | WBGene00009647 |  |
| CYB2_YEAST | nonessential | F41E6.5 | WBGene00018286 |  |
| DAN4_YEAST | nonessential | Y51B11A.a | WBGene00021761 |  |
| DAP1_YEAST | nonessential | K07E3.8 | WBGene00006890 |  |
| DBP5_YEAST | essential | T07D4.4 | WBGene00011580 |  |
| DBP9_YEAST | essential | C24H12.4 | WBGene00016073 | x |
| DBR1_YEAST | nonessential | C55B7.8 | WBGene00000937 |  |
| DCAM_YEAST | nonessential | F47G4.7 | WBGene00004875 |  |
| DCTD_YEAST | nonessential | ZK643.2 | WBGene00014034 |  |
| DF10_YEAST | nonessential | B0024.13 | WBGene00007102 |  |
| DHH1_YEAST | nonessential | C07H6.5 | WBGene00000479 | x |
| DHR1_YEAST | essential | C06E1.10 | WBGene00015525 | x |
| DHSB_YEAST | nonessential | F42A8.2 | WBGene00006433 | x |
| DIE2_YEAST | nonessential | T24D1.4 | WBGene00011987 |  |
| DIM1_YEAST | essential | E02H1.1 | WBGene00008455 | x |
| DIP2_YEAST | essential | F13H8.2 | WBGene00017435 |  |
| DLDH_YEAST | nonessential | LLC1.3 | WBGene00010794 | x |
| DNA2_YEAST | essential | F43G6.1 | WBGene00001016 |  |
| DNLI_YEAST | essential | C29A12.3 | WBGene00002985 | x |
| DNM1_YEAST | nonessential | T12E12.4 | WBGene00001093 |  |
| DNPE_YEAST | nonessential | F01F1.9 | WBGene00017163 |  |
| DO34_YEAST | nonessential | R74.6 | WBGene00011280 |  |
| DOA1_YEAST | nonessential | C05C10.6 | WBGene00007333 |  |
| DOR1_YEAST | nonessential | T12D8.9 | WBGene00011736 |  |
| DPB2_YEAST | essential | F08B4.5 | WBGene00017237 |  |
| DPB3_YEAST | nonessential | Y53F4B.d | WBGene00013150 |  |
| DPD2_YEAST | essential | F12F6.7 | WBGene00008722 | x |
| DPD3_YEAST | nonessential | R04F11.3 | WBGene00011016 |  |
| DPH2_YEAST | nonessential | C09G5.3 | WBGene00007488 |  |
| DPH5_YEAST | nonessential | B0491.7 | WBGene00007194 |  |
| DPO2_YEAST | essential | R01H10.1 | WBGene00001002 | x |
| DPOA_YEAST | essential | Y47D3A.c | WBGene00012936 | x |
| DPOA_YEAST | essential | Y47D3A.d | WBGene00012936 | x |
| DPOD_YEAST | essential | F10C2.4 | WBGene00008645 | x |
| DPOE_YEAST | essential | F33H2.5 | WBGene00009368 | x |
| DPOG_YEAST | nonessential | Y57A10A.m | WBGene00013258 |  |
| DPP3_YEAST | nonessential | F02E9.8 | WBGene00008532 |  |
| DR48_YEAST | nonessential | F10F2.9 | WBGene00004118 |  |
| DRS1_YEAST | essential | Y71G12A_201.a | WBGene00022148 |  |
| DRS1_YEAST | essential | Y71G12A_203.c | WBGene00022148 |  |
| DSK2_YEAST | nonessential | F15C11.2 | WBGene00008852 |  |
| DTD_YEAST | nonessential | T16G1.10 | WBGene00004151 |  |
| DUR3_YEAST | nonessential | C48D1.3 | WBGene00000501 |  |
| DYHC_YEAST | nonessential | T21E12.4 | WBGene00000962 | x |
| DYL1_YEAST | nonessential | T26A5.9 | WBGene00001005 | x |
| DYR_YEAST | essential | C36B1.7 | WBGene00007974 |  |
| E2BA_YEAST | nonessential | ZK1098.4 | WBGene00014221 |  |
| E2BD_YEAST | essential | F11A3.2 | WBGene00008670 | x |
| E2BE_YEAST | essential | D2085.3 | WBGene00008428 | x |
| EF2_YEAST | nonessential | F25H5.4 | WBGene00001167 | x |
| EFG1_YEAST | nonessential | F29C12.4 | WBGene00009246 | x |
| EFTU_YEAST | nonessential | Y71H2_378.a | WBGene00007000 | x |
| EGD2_YEAST | nonessential | Y65B4B_10.b | WBGene00022042 | x |
| EGD2_YEAST | nonessential | Y65B4B_10.d | WBGene00022042 | x |
| EM24_YEAST | nonessential | W02D7.7 | WBGene00004766 |  |
| END1_YEAST | nonessential | R06F6.2 | WBGene00011067 |  |
| ENP1_YEAST | essential | F57B9.5 | WBGene00000276 | x |
| ER19_YEAST | essential | Y48B6A.13a | WBGene00012984 |  |
| ERF1_YEAST | essential | T05H4.6 | WBGene00020269 | x |
| ERS1_YEAST | nonessential | C41C4.7 | WBGene00008052 |  |
| ERV1_YEAST | essential | F56C11.3 | WBGene00018955 |  |
| ESA1_YEAST | essential | VC5.4 | WBGene00007029 | x |
| ETFA_YEAST | nonessential | F27D4.1 | WBGene00009187 | x |
| ETFB_YEAST | nonessential | F23C8.5 | WBGene00017734 |  |
| ETFD_YEAST | nonessential | C05D11.12 | WBGene00002855 | x |
| F16P_YEAST | nonessential | K07A3.b | WBGene00001404 |  |
| FAB1_YEAST | nonessential | C05E7.5 | WBGene00004089 |  |
| FAB1_YEAST | nonessential | VF11C1L.1 | WBGene00004089 |  |
| FAD1_YEAST | essential | R53.1 | WBGene00011271 |  |
| FBRL_YEAST | essential | T01C3.7 | WBGene00001423 | x |
| FKB2_YEAST | nonessential | F36H1.1 | WBGene00001426 |  |
| FKH2_YEAST | nonessential | T28H11.4 | WBGene00003976 |  |
| FLX1_YEAST | nonessential | K01C8.7 | WBGene00010459 |  |
| FMS1_YEAST | nonessential | R13G10.3 | WBGene00000137 |  |
| FMS1_YEAST | nonessential | R13G10.2 | WBGene00000137 |  |
| FOLE_YEAST | nonessential | F25B5.6 | WBGene00017777 |  |
| FOX2_YEAST | nonessential | M03A8.1 | WBGene00000991 |  |
| FRDA_YEAST | nonessential | F59G1.7 | WBGene00001486 |  |
| FTHC_YEAST | nonessential | Y106G6E.4 | WBGene00013708 |  |
| FU26_YEAST | nonessential | F36H2.2 | WBGene00009499 |  |
| FUMH_YEAST | nonessential | H14A12.2 | WBGene00001503 | x |
| G6PD_YEAST | nonessential | B0035.5 | WBGene00007108 |  |
| G6PI_YEAST | nonessential | Y87G2A.q | WBGene00013597 |  |
| GAA1_YEAST | essential | F33D11.9 | WBGene00018006 |  |
| GALX_YEAST | nonessential | C47B2.6 | WBGene00008132 |  |
| GALY_YEAST | nonessential | C24A8.2 | WBGene00004106 |  |
| GALY_YEAST | nonessential | C24A8.3 | WBGene00004106 |  |
| GAR1_YEAST | essential | Y66H1A.4 | WBGene00022046 |  |
| GATA_YEAST | nonessential | K04D7.3 | WBGene00001794 |  |
| GATH_YEAST | nonessential | Y41D4A_3192.a | WBGene00021508 |  |
| GBLP_YEAST | nonessential | K04D7.1 | WBGene00010556 | x |
| GC14_YEAST | essential | W02A11.1 | WBGene00012192 |  |
| GC20_YEAST | nonessential | F42A10.1 | WBGene00018339 |  |
| GCH1_YEAST | essential | F32G8.6 | WBGene00000298 |  |
| GCN2_YEAST | nonessential | Y81G3A.3 | WBGene00013591 |  |
| GCN5_YEAST | nonessential | Y47G6A_241.b | WBGene00021636 |  |
| GCR3_YEAST | nonessential | F37E3.1 | WBGene00018156 | x |
| GCS1_YEAST | nonessential | K02B12.7 | WBGene00010500 |  |
| GCSP_YEAST | nonessential | R12C12.1 | WBGene00020022 |  |
| GCST_YEAST | nonessential | F25B4.1 | WBGene00017765 |  |
| GDA1_YEAST | nonessential | K08H10.4 | WBGene00010697 |  |
| GDIR_YEAST | nonessential | F46H6.1 | WBGene00004356 |  |
| GEF1_YEAST | nonessential | C07H4.2 | WBGene00000532 |  |
| GIR2_YEAST | nonessential | T26E3.4 | WBGene00012037 |  |
| GLGB_YEAST | nonessential | T04A8.7 | WBGene00011409 |  |
| GLN3_YEAST | nonessential | W09C2.1 | WBGene00001249 | x |
| GLO3_YEAST | nonessential | F07F6.4 | WBGene00017217 |  |
| GLPK_YEAST | nonessential | R11F4.1 | WBGene00020007 |  |
| GLT1_YEAST | nonessential | W07E11.3 | WBGene00012326 | x |
| GLT1_YEAST | nonessential | W07E11.1 | WBGene00012326 |  |
| GLY1_YEAST | nonessential | R102.4 | WBGene00011291 |  |
| GLYC_YEAST | nonessential | C05D11.11 | WBGene00003214 | x |
| GP12_YEAST | essential | Y52B11C.1 | WBGene00013131 |  |
| GPI1_YEAST | nonessential | F01G4.5 | WBGene00008504 |  |
| GPI3_YEAST | essential | D2085.6 | WBGene00008431 |  |
| GPI8_YEAST | essential | T05E11.6 | WBGene00011482 |  |
| GRPE_YEAST | essential | C34C12.8 | WBGene00007927 |  |
| GSH1_YEAST | nonessential | F37B12.2 | WBGene00001527 |  |
| GSHB_YEAST | nonessential | M176.2 | WBGene00010941 |  |
| GSHR_YEAST | nonessential | C46F11.2 | WBGene00008117 |  |
| GTR1_YEAST | nonessential | T24F1.1 | WBGene00006414 |  |
| GTR2_YEAST | nonessential | Y24F12A.a | WBGene00012497 |  |
| GUAA_YEAST | nonessential | M106.4 | WBGene00010912 |  |
| GUAD_YEAST | nonessential | F38E11.3 | WBGene00000775 |  |
| GUF1_YEAST | nonessential | ZK1236.1 | WBGene00022862 |  |
| GYP1_YEAST | nonessential | F32B6.8 | WBGene00009322 |  |
| GYP2_YEAST | nonessential | Y38H8A.1 | WBGene00012868 |  |
| GYP2_YEAST | nonessential | Y45F10A.6 | WBGene00012868 |  |
| H2AV_YEAST | nonessential | R08C7.3 | WBGene00019947 |  |
| HAP2_YEAST | nonessential | Y53H1A.d | WBGene00013178 |  |
| HAS1_YEAST | essential | B0511.6 | WBGene00015232 | x |
| HAT1_YEAST | nonessential | M03C11.4 | WBGene00010841 |  |
| HCM1_YEAST | nonessential | C25A1.2 | WBGene00001442 |  |
| HDA1_YEAST | nonessential | F41H10.6 | WBGene00018319 |  |
| HMCS_YEAST | essential | F25B4.6 | WBGene00017769 | x |
| HMT1_YEAST | nonessential | Y113G7B.17 | WBGene00013766 |  |
| HNT1_YEAST | nonessential | F21C3.3 | WBGene00009002 |  |
| HR25_YEAST | nonessential | C03C10.1 | WBGene00002202 |  |
| HS49_YEAST | essential | C08B11.5 | WBGene00004723 | x |
| HST2_YEAST | nonessential | R11A8.4 | WBGene00004800 |  |
| HUL5_YEAST | nonessential | Y39A1C.2 | WBGene00003898 |  |
| IDH2_YEAST | nonessential | F43G9.1 | WBGene00009664 | x |
| IF1A_YEAST | essential | H06H21.3 | WBGene00019162 | x |
| IF2B_YEAST | essential | K04G2.1 | WBGene00010560 | x |
| IF2G_YEAST | essential | Y39G10A_246.h | WBGene00021466 | x |
| IF2G_YEAST | essential | Y39G10A_246.c | WBGene00021466 | x |
| IF2M_YEAST | nonessential | F46B6.6 | WBGene00009771 |  |
| IF32_YEAST | essential | Y39G10A_237.b | WBGene00001232 | x |
| IF34_YEAST | essential | F22B5.2 | WBGene00001230 | x |
| IF39_YEAST | essential | Y54E2A.11 | WBGene00001225 | x |
| IF3A_YEAST | essential | C27D11.1 | WBGene00001209 | x |
| IF3X_YEAST | nonessential | F55H2.6 | WBGene00000550 |  |
| IF3Y_YEAST | essential | ZK858.7 | WBGene00014120 |  |
| IF5_YEAST | essential | C37C3.2 | WBGene00016496 |  |
| IF6_YEAST | essential | C47B2.5 | WBGene00001234 | x |
| IKI3_YEAST | nonessential | Y110A7A.e | WBGene00022463 |  |
| IM13_YEAST | nonessential | DY3.1 | WBGene00006574 |  |
| IM17_YEAST | essential | E04A4.5 | WBGene00017119 | x |
| IM44_YEAST | essential | T09B4.9 | WBGene00020383 | x |
| IMA1_YEAST | essential | F32E10.4 | WBGene00002074 | x |
| IMB2_YEAST | nonessential | R06A4.4 | WBGene00002076 | x |
| IMB3_YEAST | essential | C53D5.a | WBGene00002077 | x |
| IMB3_YEAST | essential | C53D5.i | WBGene00002077 | x |
| IME2_YEAST | nonessential | M04C9.5 | WBGene00010860 |  |
| IMP3_YEAST | essential | C48B6.2 | WBGene00016740 | x |
| IMP4_YEAST | essential | ZK795.3 | WBGene00014083 | x |
| INO1_YEAST | nonessential | VF13D12L.1 | WBGene00012148 |  |
| IPYR_YEAST | essential | C47E12.4 | WBGene00008149 | x |
| IRE1_YEAST | nonessential | C41C4.4 | WBGene00002147 |  |
| ISC1_YEAST | nonessential | T27F6.6 | WBGene00012105 |  |
| ISY1_YEAST | nonessential | F53B7.3 | WBGene00009966 | x |
| KAD1_YEAST | nonessential | C29E4.8 | WBGene00016205 | x |
| KAPR_YEAST | nonessential | R07E4.6 | WBGene00002190 |  |
| KAR3_YEAST | nonessential | T09A5.2 | WBGene00002216 |  |
| KC2C_YEAST | nonessential | T01G9.6 | WBGene00002196 |  |
| KEM1_YEAST | nonessential | Y39G8C.1 | WBGene00012730 |  |
| KEM1_YEAST | nonessential | Y39G8C.b | WBGene00012730 |  |
| KGUA_YEAST | essential | T03F1.8 | WBGene00020190 |  |
| KIME_YEAST | essential | Y42G9A.c | WBGene00021534 |  |
| KIP1_YEAST | nonessential | F23B12.8 | WBGene00000257 |  |
| KYNU_YEAST | nonessential | C15H9.7 | WBGene00015802 |  |
| LA17_YEAST | nonessential | Y63D3A.5 | WBGene00006565 | x |
| LAH1_YEAST | nonessential | C44E4.4 | WBGene00016653 | x |
| LCB1_YEAST | essential | C23H3.4 | WBGene00016020 | x |
| LCP5_YEAST | essential | C48E7.3 | WBGene00003059 | x |
| LEO1_YEAST | nonessential | B0035.11 | WBGene00007110 |  |
| LIP5_YEAST | nonessential | M01F1.3 | WBGene00010809 | x |
| LONM_YEAST | nonessential | C34B2.6 | WBGene00016391 |  |
| LOS1_YEAST | nonessential | C49H3.10 | WBGene00002080 |  |
| LSM1_YEAST | nonessential | F40F8.9 | WBGene00003076 |  |
| LSM2_YEAST | essential | T10G3.6 | WBGene00001808 |  |
| LSM3_YEAST | essential | Y62E10A.l | WBGene00003077 |  |
| LSM4_YEAST | essential | F32A5.7 | WBGene00003078 |  |
| LSM5_YEAST | essential | F28F8.3 | WBGene00003079 |  |
| LSM6_YEAST | nonessential | Y71G12A_187.b | WBGene00003080 |  |
| LSM7_YEAST | nonessential | ZK593.7 | WBGene00003081 |  |
| LYS9_YEAST | nonessential | R02D3.1 | WBGene00019819 |  |
| MAD2_YEAST | nonessential | Y69A2A_2326.a | WBGene00003161 |  |
| MAF1_YEAST | nonessential | C43H8.2 | WBGene00016622 |  |
| MAK3_YEAST | nonessential | B0238.10 | WBGene00015074 |  |
| MAK5_YEAST | essential | F55F8.2 | WBGene00018890 | x |
| MAOX_YEAST | nonessential | Y48B6A.12 | WBGene00012983 |  |
| MCM2_YEAST | essential | Y17G7B.5 | WBGene00003154 | x |
| MCM5_YEAST | essential | R10E4.4 | WBGene00003157 | x |
| MCX1_YEAST | nonessential | D2030.2 | WBGene00008412 |  |
| MDHM_YEAST | nonessential | F20H11.3 | WBGene00003162 | x |
| MDN1_YEAST | essential | F55F10.1 | WBGene00018898 | x |
| MDN1_YEAST | essential | F55F10.2 | WBGene00018898 | x |
| MED7_YEAST | nonessential | Y54E5B.3 | WBGene00002324 |  |
| MEU1_YEAST | nonessential | B0228.7 | WBGene00015064 |  |
| MK16_YEAST | essential | C16A3.6 | WBGene00015811 | x |
| MK21_YEAST | essential | F23B12.7 | WBGene00009084 | x |
| MLH1_YEAST | nonessential | T28A8.7 | WBGene00003373 |  |
| MMS2_YEAST | nonessential | F39B2.2 | WBGene00006730 |  |
| MOD5_YEAST | nonessential | ZC395.6 | WBGene00001740 |  |
| MOT1_YEAST | essential | F15D4.1 | WBGene00000274 |  |
| MP10_YEAST | essential | Y75B8A.7 | WBGene00013544 | x |
| MPG1_YEAST | essential | C42C1.5 | WBGene00016583 | x |
| MPPB_YEAST | essential | ZC410.2 | WBGene00013880 |  |
| MR11_YEAST | nonessential | ZC302.1 | WBGene00003405 |  |
| MSH2_YEAST | nonessential | H26D21.2 | WBGene00003418 |  |
| MSH4_YEAST | nonessential | ZK1127.11 | WBGene00001872 |  |
| MSH4_YEAST | nonessential | ZK1127.1 | WBGene00001872 |  |
| MSH5_YEAST | nonessential | F09E8.3 | WBGene00003421 |  |
| MSH5_YEAST | nonessential | F09E8.4 | WBGene00003421 |  |
| MSH6_YEAST | nonessential | Y47G6A_242.c | WBGene00003422 |  |
| MSN4_YEAST | nonessential | F56F11.3 | WBGene00018990 |  |
| MSP1_YEAST | nonessential | K04D7.2 | WBGene00010557 |  |
| MSRA_YEAST | nonessential | F43E2.5 | WBGene00018393 |  |
| MSS1_YEAST | nonessential | F39B2.7 | WBGene00009557 |  |
| MSS4_YEAST | essential | F55A12.3 | WBGene00004087 | x |
| MTHS_YEAST | nonessential | C06A8.1 | WBGene00015512 |  |
| MTO1_YEAST | nonessential | F52H3.2 | WBGene00009944 |  |
| MTR4_YEAST | essential | W08D2.7 | WBGene00012342 | x |
| MTRA_YEAST | nonessential | F53G2.6 | WBGene00006647 | x |
| N145_YEAST | essential | ZK328.5 | WBGene00003796 | x |
| NAB4_YEAST | essential | R10E9.1 | WBGene00003423 |  |
| NADE_YEAST | essential | C24F3.4 | WBGene00007698 |  |
| NAH2_YEAST | nonessential | F57C7.2 | WBGene00003733 |  |
| NAP1_YEAST | nonessential | D2096.8 | WBGene00017075 |  |
| NAT1_YEAST | nonessential | Y50D7_162.b | WBGene00021754 |  |
| NAT1_YEAST | nonessential | Y50D7_164.a | WBGene00021754 |  |
| NB35_YEAST | essential | F10G8.6 | WBGene00008664 |  |
| NCL1_YEAST | nonessential | Y48G8A_2614.a | WBGene00021686 |  |
| NCPR_YEAST | nonessential | K10D2.6 | WBGene00019632 | x |
| NDK_YEAST | nonessential | F25H2.5 | WBGene00009119 |  |
| NEP1_YEAST | essential | Y39A1A.14 | WBGene00012652 |  |
| NFU1_YEAST | nonessential | R10H10.1 | WBGene00003064 |  |
| NHP2_YEAST | essential | Y48A6B.3 | WBGene00012964 | x |
| NHPX_YEAST | nonessential | M28.5 | WBGene00010896 | x |
| NIP7_YEAST | essential | C43E11.9 | WBGene00016607 | x |
| NMD3_YEAST | essential | T25G3.3 | WBGene00012030 | x |
| NMT_YEAST | essential | T17E9.2 | WBGene00020549 | x |
| NOG1_YEAST | essential | T07A9.9 | WBGene00020297 | x |
| NOP2_YEAST | essential | W07E6.1 | WBGene00021073 | x |
| NOP4_YEAST | essential | R05H10.2 | WBGene00011043 | x |
| NOP5_YEAST | essential | W01B11.3 | WBGene00020915 |  |
| NOT1_YEAST | essential | F57B9.2 | WBGene00003824 |  |
| NPR2_YEAST | nonessential | F49E8.1 | WBGene00018635 |  |
| NRD1_YEAST | essential | D1007.7 | WBGene00017004 |  |
| NRK1_YEAST | essential | T19A5.2 | WBGene00001526 |  |
| NTF2_YEAST | essential | R05D11.3 | WBGene00004305 | x |
| NU49_YEAST | essential | Y54E5A.4 | WBGene00003790 | x |
| O14467 | nonessential | H21P03.1 | WBGene00003148 |  |
| OAT_YEAST | nonessential | C16A3.10 | WBGene00015814 |  |
| ODO1_YEAST | nonessential | T22B11.5 | WBGene00020679 | x |
| ODO2_YEAST | nonessential | W02F12.5 | WBGene00020950 | x |
| ODP2_YEAST | nonessential | F23B12.5 | WBGene00009082 | x |
| ODPA_YEAST | nonessential | T05H10.6 | WBGene00011510 | x |
| ODPB_YEAST | nonessential | C04C3.3 | WBGene00015413 |  |
| OM20_YEAST | nonessential | F23H12.2 | WBGene00009092 |  |
| OM40_YEAST | essential | C18E9.6 | WBGene00007686 | x |
| ORC2_YEAST | essential | F59E10.1 | WBGene00003882 |  |
| ORN_YEAST | nonessential | C08B6.8 | WBGene00007429 |  |
| OSTA_YEAST | essential | T22D1.4 | WBGene00020683 |  |
| OSTB_YEAST | essential | T09A5.11 | WBGene00011638 | x |
| OSTE_YEAST | essential | F57B10.10 | WBGene00000896 | x |
| OSTG_YEAST | nonessential | ZK686.3 | WBGene00022793 | x |
| OXA1_YEAST | nonessential | C01A2.3 | WBGene00007215 | x |
| PAC1_YEAST | nonessential | T03F6.5 | WBGene00003047 |  |
| PAC2_YEAST | nonessential | K07H8.1 | WBGene00019503 |  |
| PAF1_YEAST | nonessential | C55A6.9 | WBGene00008338 | x |
| PAN3_YEAST | nonessential | ZK632.7 | WBGene00014015 |  |
| PBP2_YEAST | nonessential | F26B1.2 | WBGene00017816 |  |
| PBS2_YEAST | nonessential | Y54E10B_152.e | WBGene00003186 | x |
| PBS2_YEAST | nonessential | Y54E10B_152.b | WBGene00003186 | x |
| PCH2_YEAST | nonessential | F10B5.5 | WBGene00008641 |  |
| PDAT_YEAST | nonessential | M05B5.4 | WBGene00010872 |  |
| PDS5_YEAST | essential | H38K22.1 | WBGene00001352 |  |
| PDX3_YEAST | nonessential | F57B9.1 | WBGene00018996 |  |
| PE11_YEAST | nonessential | ZK1128.8 | WBGene00014234 |  |
| PEP3_YEAST | nonessential | W06B4.3 | WBGene00021058 |  |
| PESC_YEAST | essential | R13A5.12 | WBGene00003063 | x |
| PESC_YEAST | essential | R13A5.13 | WBGene00003063 | x |
| PET8_YEAST | nonessential | D1046.3 | WBGene00008364 |  |
| PEX1_YEAST | nonessential | C11H1.4 | WBGene00004191 |  |
| PEX1_YEAST | nonessential | C11H1.6 | WBGene00004191 |  |
| PEX5_YEAST | nonessential | C34C6.6 | WBGene00004194 | x |
| PEX6_YEAST | nonessential | F39G3.7 | WBGene00004195 |  |
| PEXC_YEAST | nonessential | F08B12.2 | WBGene00004197 |  |
| PEXD_YEAST | nonessential | F32A5.6 | WBGene00004198 |  |
| PFD2_YEAST | nonessential | H20J04.d | WBGene00019220 |  |
| PFD3_YEAST | nonessential | T06G6.9 | WBGene00006889 | x |
| PFD4_YEAST | nonessential | B0035.4 | WBGene00007107 |  |
| PFD5_YEAST | nonessential | R151.9 | WBGene00020112 |  |
| PFD6_YEAST | nonessential | F21C3.5 | WBGene00009004 | x |
| PGK_YEAST | nonessential | T03F1.3 | WBGene00020185 |  |
| PHSG_YEAST | nonessential | F40C5.b | WBGene00020696 |  |
| PHSG_YEAST | nonessential | F40C5.c | WBGene00020696 |  |
| PHSG_YEAST | nonessential | F40C5.d | WBGene00020696 |  |
| PHSG_YEAST | nonessential | F40C5.a | WBGene00020696 |  |
| PHSG_YEAST | nonessential | F40C5.e | WBGene00020696 |  |
| PHSG_YEAST | nonessential | F40C5.h | WBGene00020696 |  |
| PHSG_YEAST | nonessential | T22F3.3 | WBGene00020696 |  |
| PIF1_YEAST | nonessential | Y18H1A_67.d | WBGene00004028 |  |
| PIF1_YEAST | nonessential | Y18H1A_67.f | WBGene00004028 |  |
| PIF1_YEAST | nonessential | Y18H1A_67.c | WBGene00004028 |  |
| PIK1_YEAST | essential | F35H12.4 | WBGene00018076 |  |
| PIS_YEAST | essential | Y46G5.e | WBGene00012897 |  |
| PLC1_YEAST | nonessential | T01E8.3 | WBGene00004038 | x |
| PMM_YEAST | essential | F52B11.2 | WBGene00009925 | x |
| PNPH_YEAST | nonessential | K02D7.1 | WBGene00019298 |  |
| POP1_YEAST | essential | C05D11.9 | WBGene00015486 |  |
| POP2_YEAST | nonessential | Y56A3A.20 | WBGene00000369 |  |
| POP4_YEAST | essential | C15C6.4 | WBGene00007603 |  |
| PPE1_YEAST | nonessential | B0464.7 | WBGene00007188 | x |
| PPT1_YEAST | nonessential | Y39B6B.ff | WBGene00012665 |  |
| PR16_YEAST | essential | K03H1.2 | WBGene00003389 | x |
| PR17_YEAST | nonessential | F49D11.1 | WBGene00018625 | x |
| PR21_YEAST | essential | W07E6.4 | WBGene00004188 | x |
| PR22_YEAST | nonessential | EEED8.5 | WBGene00003393 | x |
| PR28_YEAST | essential | F01F1.7 | WBGene00017162 | x |
| PR31_YEAST | essential | Y110A7A.m | WBGene00022458 | x |
| PR39_YEAST | essential | F25B4.5 | WBGene00017768 |  |
| PR40_YEAST | essential | ZK1098.7 | WBGene00014218 |  |
| PR40_YEAST | essential | ZK1098.1 | WBGene00014218 |  |
| PR43_YEAST | essential | F56D2.6 | WBGene00018967 | x |
| PR46_YEAST | essential | D1054.15 | WBGene00006481 | x |
| PRI2_YEAST | essential | W02D9.1 | WBGene00004181 | x |
| PROA_YEAST | nonessential | T22H6.2 | WBGene00011938 |  |
| PROC_YEAST | essential | M153.1 | WBGene00010924 |  |
| PROF_YEAST | essential | K03E6.6 | WBGene00003991 |  |
| PRP3_YEAST | essential | M03C11.7 | WBGene00010844 | x |
| PRP4_YEAST | essential | C36B1.5 | WBGene00007972 | x |
| PRP9_YEAST | essential | T13H5.4 | WBGene00011758 | x |
| PRS6_YEAST | essential | F23F12.6 | WBGene00004503 | x |
| PRS7_YEAST | essential | C52E4.4 | WBGene00004501 | x |
| PRSA_YEAST | nonessential | F56H1.4 | WBGene00004505 | x |
| PSA1_YEAST | essential | CD4.6 | WBGene00003927 | x |
| PSA2_YEAST | essential | D1054.2 | WBGene00003923 |  |
| PSA5_YEAST | essential | F25H2.9 | WBGene00003926 | x |
| PSA7_YEAST | essential | C36B1.4 | WBGene00003925 | x |
| PSB1_YEAST | essential | C02F5.3 | WBGene00003952 | x |
| PSB2_YEAST | essential | T20F5.2 | WBGene00003950 | x |
| PSB3_YEAST | essential | Y38A8.2 | WBGene00003949 | x |
| PSB4_YEAST | essential | F39H11.5 | WBGene00003953 | x |
| PSB5_YEAST | essential | K05C4.1 | WBGene00003951 | x |
| PSB7_YEAST | essential | C47B2.4 | WBGene00003948 | x |
| PSD1_YEAST | nonessential | B0361.5 | WBGene00015159 |  |
| PSD9_YEAST | nonessential | C44B7.1 | WBGene00016623 |  |
| PSDA_YEAST | nonessential | F40G9.1 | WBGene00018239 |  |
| PSDA_YEAST | nonessential | W10C4.b | WBGene00018239 | x |
| PSF2_YEAST | essential | F31C3.5 | WBGene00009287 | x |
| PSP2_YEAST | nonessential | T23C6.2 | WBGene00020726 |  |
| PT56_YEAST | nonessential | Y45F3A.9 | WBGene00012864 |  |
| PT91_YEAST | nonessential | Y18D10A.16 | WBGene00012483 |  |
| PUF3_YEAST | nonessential | W06B11.2 | WBGene00004245 |  |
| PUR1_YEAST | nonessential | T04A8.5 | WBGene00011407 |  |
| PUR2_YEAST | nonessential | F38B6.4 | WBGene00018174 |  |
| PUR4_YEAST | nonessential | F10F2.2 | WBGene00008654 |  |
| PUR8_YEAST | essential | R06C7.5 | WBGene00011064 | x |
| PURA_YEAST | nonessential | C37H5.6 | WBGene00016509 |  |
| PUS3_YEAST | nonessential | E02H1.3 | WBGene00006473 |  |
| PUT2_YEAST | nonessential | Y57G7A.10 | WBGene00000112 |  |
| PUT2_YEAST | nonessential | F56D12.1 | WBGene00000112 |  |
| PWP2_YEAST | essential | F55F8.3 | WBGene00018891 | x |
| PYR1_YEAST | nonessential | D2085.1 | WBGene00004259 | x |
| PYRD_YEAST | nonessential | W02D3.2 | WBGene00020932 |  |
| PYRF_YEAST | nonessential | T07C4.1 | WBGene00011559 |  |
| Q02804 | nonessential | Y54E10B_159.i | WBGene00021841 |  |
| Q02875 | nonessential | C18H9.3 | WBGene00016002 |  |
| Q02889 | nonessential | F15D4.3 | WBGene00008860 |  |
| Q02890 | nonessential | Y53H1C.a | WBGene00010160 |  |
| Q02908 | nonessential | ZK863.3 | WBGene00014123 |  |
| Q03195 | essential | Y39E4B.1 | WBGene00012714 | x |
| Q03201 | essential | Y37D8A.18 | WBGene00012556 |  |
| Q03390 | nonessential | F41E6.9 | WBGene00018290 |  |
| Q03761 | essential | Y56A3A.4 | WBGene00006396 |  |
| Q03778 | essential | R10H10.6 | WBGene00011224 |  |
| Q03786 | nonessential | F26D11.1 | WBGene00017818 |  |
| Q03920 | nonessential | C33C12.9 | WBGene00016341 |  |
| Q03940 | essential | C27H6.2 | WBGene00007784 | x |
| Q04048 | essential | C50F2.3 | WBGene00016837 | x |
| Q04049 | nonessential | F53A3.2 | WBGene00018721 |  |
| Q04081 | nonessential | B0285.4 | WBGene00007137 |  |
| Q04149 | nonessential | C43E11.2 | WBGene00016602 |  |
| Q04311 | nonessential | K06H7.3 | WBGene00019457 |  |
| Q04396 | nonessential | T28D9.3 | WBGene00020895 |  |
| Q04430 | essential | C10G11.5 | WBGene00004068 |  |
| Q04600 | nonessential | C25H3.4 | WBGene00016113 |  |
| Q05498 | essential | F30A10.9 | WBGene00009266 | x |
| Q05521 | nonessential | F53C3.13 | WBGene00018756 |  |
| Q05583 | essential | Y18D10A.9 | WBGene00012479 |  |
| Q05946 | essential | Y53C12B.1 | WBGene00013143 | x |
| Q06102 | essential | F11A10.3 | WBGene00008689 | x |
| Q06106 | essential | T23F6.4 | WBGene00004315 | x |
| Q06132 | essential | Y52B11A.10 | WBGene00013129 |  |
| Q06143 | nonessential | K11G12.5 | WBGene00019656 |  |
| Q06152 | nonessential | T04H1.5 | WBGene00011451 |  |
| Q06338 | essential | ZK1127.4 | WBGene00022851 | x |
| Q06344 | essential | F58B3.4 | WBGene00010231 | x |
| Q06385 | nonessential | Y47G6A_247.a | WBGene00021644 | x |
| Q06505 | essential | F13B12.1 | WBGene00008729 |  |
| Q06510 | nonessential | ZK809.2 | WBGene00006491 |  |
| Q06632 | essential | Y76B12C_65.a | WBGene00022301 | x |
| Q06632 | essential | Y76B12C_66.c | WBGene00022301 | x |
| Q06672 | nonessential | Y51H4A.m | WBGene00013109 | x |
| Q06685 | nonessential | F46F11.1 | WBGene00018508 |  |
| Q06696 | nonessential | F17C11.8 | WBGene00008919 |  |
| Q07381 | essential | F10G7.1 | WBGene00006497 | x |
| Q07457 | nonessential | R05D3.4 | WBGene00007008 |  |
| Q07508 | nonessential | B0495.8 | WBGene00015207 |  |
| Q07830 | essential | C27A12.9 | WBGene00016159 |  |
| Q07896 | essential | C37H5.5 | WBGene00016508 | x |
| Q07914 | essential | T19B4.4 | WBGene00001039 |  |
| Q08004 | nonessential | Y56A3A.18 | WBGene00013236 |  |
| Q08023 | nonessential | K11D2.1 | WBGene00010768 |  |
| Q08444 | essential | Y54E10B_159.e | WBGene00021843 |  |
| Q08444 | essential | Y54E10B_159.g | WBGene00021843 |  |
| Q08685 | essential | F59A2.4 | WBGene00010304 |  |
| Q08723 | essential | R12E2.3 | WBGene00004464 | x |
| Q08726 | essential | B0207.6 | WBGene00015029 |  |
| Q08920 | nonessential | F26A3.2 | WBGene00009141 | x |
| Q08951 | nonessential | W09G10.4 | WBGene00000162 |  |
| Q08952 | nonessential | F52E1.13 | WBGene00018700 |  |
| Q08963 | nonessential | H20J04.c | WBGene00019223 |  |
| Q08971 | nonessential | Y54E5A.5 | WBGene00013200 |  |
| Q12000 | nonessential | F27D4.4 | WBGene00009189 |  |
| Q12004 | essential | ZK1128.4 | WBGene00014230 |  |
| Q12028 | nonessential | Y105E8C.n | WBGene00000158 | x |
| Q12029 | nonessential | C41C4.2 | WBGene00005150 |  |
| Q12035 | essential | Y49F6B.q | WBGene00021715 |  |
| Q12049 | nonessential | F01F1.1 | WBGene00017158 |  |
| Q12052 | nonessential | T08G11.4 | WBGene00011631 | x |
| Q12059 | nonessential | C26E6.8 | WBGene00006735 |  |
| Q12090 | nonessential | F52C9.4 | WBGene00004095 |  |
| Q12102 | essential | F09G2.4 | WBGene00017313 | x |
| Q12118 | nonessential | R05F9.10 | WBGene00019893 |  |
| Q12142 | nonessential | T22H9.2 | WBGene00020706 |  |
| Q12186 | essential | Y116A8C.32 | WBGene00013808 | x |
| Q12199 | nonessential | C02C2.6 | WBGene00022803 |  |
| Q12275 | nonessential | F28B3.1 | WBGene00017882 | x |
| Q12280 | nonessential | F09C3.1 | WBGene00003980 |  |
| Q12309 | essential | M03F8.3 | WBGene00019762 |  |
| Q12311 | nonessential | F42A9.2 | WBGene00003034 |  |
| Q12354 | nonessential | K04G2.5 | WBGene00010564 |  |
| Q12368 | essential | ZK686.4 | WBGene00022794 |  |
| Q12383 | nonessential | Y49A3A.3 | WBGene00013026 |  |
| Q12395 | nonessential | H38K22.2 | WBGene00010428 | x |
| Q12400 | nonessential | F25H8.1 | WBGene00009131 |  |
| Q12449 | nonessential | C01G10.8 | WBGene00007235 |  |
| Q12453 | nonessential | W07G4.3 | WBGene00012337 |  |
| Q12463 | nonessential | Y71F9A_270.a | WBGene00022107 |  |
| Q12463 | nonessential | Y71F9A_270.b | WBGene00022107 |  |
| Q12464 | essential | T22D1.10 | WBGene00020687 | x |
| Q12468 | nonessential | B0547.1 | WBGene00000817 |  |
| Q12481 | essential | T22H9.1 | WBGene00020705 |  |
| Q12483 | nonessential | C27F2.5 | WBGene00016167 |  |
| Q12500 | nonessential | T26A5.6 | WBGene00020822 |  |
| Q12511 | nonessential | ZK973.a | WBGene00022832 |  |
| Q874G8 | nonessential | Y49A3A.2 | WBGene00013025 | x |
| Q92317 | essential | F53A2.5 | WBGene00001092 |  |
| Q99190 | essential | C15F1.c | WBGene00000198 |  |
| Q99207 | essential | Y48G1A_54.d | WBGene00021660 | x |
| Q99216 | essential | Y53C12B.2 | WBGene00013144 |  |
| Q99247 | nonessential | F35G12.4 | WBGene00009441 |  |
| Q99344 | nonessential | F11H8.1 | WBGene00004341 |  |
| QOR_YEAST | nonessential | F39B2.3 | WBGene00009554 |  |
| QRI7_YEAST | nonessential | C01G10.10 | WBGene00007237 |  |
| R10A_YEAST | nonessential | Y71F9A_294.c | WBGene00004412 | x |
| R161_YEAST | nonessential | F58G6.1 | WBGene00010272 |  |
| R167_YEAST | nonessential | T04C9.1 | WBGene00020209 |  |
| R167_YEAST | nonessential | ZK328.3 | WBGene00020209 |  |
| RA14_YEAST | nonessential | K07G5.2 | WBGene00006963 |  |
| RA23_YEAST | nonessential | ZK20.3 | WBGene00013924 |  |
| RA27_YEAST | nonessential | Y47G6A_247.i | WBGene00000794 | x |
| RA50_YEAST | nonessential | T04H1.4 | WBGene00004296 |  |
| RA51_YEAST | nonessential | Y43C5A.6 | WBGene00004297 |  |
| RA54_YEAST | nonessential | W06D4.6 | WBGene00004298 |  |
| RAD1_YEAST | nonessential | C47D12.8 | WBGene00008140 |  |
| RAD2_YEAST | nonessential | F57B10.6 | WBGene00019004 |  |
| RAD5_YEAST | nonessential | F54E12.2 | WBGene00010061 |  |
| RAM1_YEAST | nonessential | F23B12.6 | WBGene00009083 |  |
| RAM2_YEAST | essential | R02D3.5 | WBGene00019823 |  |
| RBSK_YEAST | nonessential | F07A11.5 | WBGene00008548 |  |
| RBX1_YEAST | essential | ZK287.5 | WBGene00004320 | x |
| RCC1_YEAST | essential | C26D10.1 | WBGene00004304 | x |
| RCL1_YEAST | essential | ZK1127.5 | WBGene00022852 | x |
| RER1_YEAST | nonessential | F46C5.8 | WBGene00009783 |  |
| REV1_YEAST | nonessential | ZK675.2 | WBGene00014066 | x |
| RF1M_YEAST | nonessential | W03F8.3 | WBGene00020993 |  |
| RFA1_YEAST | essential | F18A1.5 | WBGene00017546 | x |
| RFC1_YEAST | essential | C54G10.2 | WBGene00004337 |  |
| RFC2_YEAST | essential | F31E3.3 | WBGene00004340 |  |
| RFC4_YEAST | essential | F58F6.4 | WBGene00004338 |  |
| RFC5_YEAST | essential | C39E9.13 | WBGene00004339 |  |
| RGD1_YEAST | nonessential | F45H7.2 | WBGene00001559 |  |
| RGD1_YEAST | nonessential | F45H7.3 | WBGene00001559 |  |
| RIM2_YEAST | nonessential | T09F3.2 | WBGene00011662 |  |
| RIO1_YEAST | essential | M01B12.5 | WBGene00019698 |  |
| RIR2_YEAST | essential | C03C10.3 | WBGene00004392 | x |
| RL10_YEAST | essential | F10B5.1 | WBGene00004421 | x |
| RL12_YEAST | nonessential | JC8.3 | WBGene00004424 | x |
| RL18_YEAST | nonessential | Y45F10D.12 | WBGene00004430 | x |
| RL19_YEAST | nonessential | C09D4.5 | WBGene00004431 | x |
| RL2_YEAST | nonessential | B0250.1 | WBGene00004413 | x |
| RL20_YEAST | nonessential | E04A4.8 | WBGene00004432 | x |
| RL20_YEAST | nonessential | Y17G9B.e | WBGene00004432 | x |
| RL23_YEAST | nonessential | B0336.10 | WBGene00004435 | x |
| RL3_YEAST | essential | F13B10.2 | WBGene00004414 | x |
| RL30_YEAST | essential | Y106G6H.3 | WBGene00004444 | x |
| RL35_YEAST | nonessential | ZK652.4 | WBGene00004449 | x |
| RL38_YEAST | nonessential | C06B8.8 | WBGene00004452 | x |
| RL39_YEAST | nonessential | C26F1.9 | WBGene00004453 |  |
| RL43_YEAST | nonessential | Y48B6A.2 | WBGene00004456 |  |
| RL5_YEAST | essential | F54C9.5 | WBGene00004416 | x |
| RLA0_YEAST | essential | F25H2.10 | WBGene00004408 | x |
| RLR1_YEAST | nonessential | C16A3.8 | WBGene00015813 |  |
| RM09_YEAST | nonessential | C26E6.6 | WBGene00016142 | x |
| RM19_YEAST | nonessential | B0303.15 | WBGene00015133 |  |
| RN15_YEAST | essential | F56A8.6 | WBGene00000774 | x |
| RNH1_YEAST | nonessential | F59A6.6 | WBGene00019088 |  |
| ROK1_YEAST | essential | R05D11.4 | WBGene00011032 |  |
| ROX1_YEAST | nonessential | T22H6.6 | WBGene00001560 |  |
| ROX1_YEAST | nonessential | C52G5.1 | WBGene00001560 |  |
| RPA2_YEAST | essential | F14B4.3 | WBGene00008781 | x |
| RPA9_YEAST | nonessential | C15H11.8 | WBGene00007616 | x |
| RPB1_YEAST | essential | F36A4.7 | WBGene00000123 |  |
| RPB2_YEAST | essential | C26E6.4 | WBGene00016140 | x |
| RPB3_YEAST | essential | C36B1.3 | WBGene00007971 | x |
| RPB6_YEAST | essential | C06A1.5 | WBGene00007355 |  |
| RPB7_YEAST | essential | Y54E10B_159.c | WBGene00021845 | x |
| RPB8_YEAST | essential | F26F4.11 | WBGene00017830 | x |
| RPBY_YEAST | essential | W01G7.3 | WBGene00012187 | x |
| RPC1_YEAST | essential | C42D4.8 | WBGene00004411 | x |
| RPC2_YEAST | essential | F09F7.3 | WBGene00017300 | x |
| RPC5_YEAST | essential | H43I07.2 | WBGene00019275 | x |
| RPC6_YEAST | essential | W09C3.4 | WBGene00021112 |  |
| RPC9_YEAST | essential | F58A4.9 | WBGene00010230 |  |
| RPCY_YEAST | essential | ZK856.10 | WBGene00014111 | x |
| RPCZ_YEAST | essential | Y77E11A_3443.q | WBGene00022309 |  |
| RPF1_YEAST | essential | F44G4.1 | WBGene00009711 | x |
| RPIA_YEAST | nonessential | B0280.3 | WBGene00015101 |  |
| RPN1_YEAST | essential | T22D1.9 | WBGene00004458 | x |
| RPN2_YEAST | essential | C23G10.4 | WBGene00004459 | x |
| RPN3_YEAST | essential | C30C11.2 | WBGene00004460 | x |
| RPN5_YEAST | essential | F10G7.8 | WBGene00004461 |  |
| RPN7_YEAST | essential | F49C12.8 | WBGene00004463 |  |
| RPN9_YEAST | nonessential | T06D8.8 | WBGene00004465 |  |
| RPNA_YEAST | nonessential | B0205.3 | WBGene00004466 |  |
| RPNC_YEAST | essential | ZK20.5 | WBGene00004468 |  |
| RR40_YEAST | essential | F59C6.4 | WBGene00010325 | x |
| RR41_YEAST | essential | B0564.1 | WBGene00007201 | x |
| RR44_YEAST | nonessential | C04G2.6 | WBGene00001001 | x |
| RR45_YEAST | essential | F37C12.13 | WBGene00018154 | x |
| RRN3_YEAST | essential | C36E8.1 | WBGene00007980 |  |
| RRP1_YEAST | essential | C47E12.7 | WBGene00008151 | x |
| RRP3_YEAST | essential | T26G10.1 | WBGene00012059 | x |
| RRP5_YEAST | essential | C16A3.3 | WBGene00015808 |  |
| RRP6_YEAST | nonessential | C14A4.4 | WBGene00000796 | x |
| RRS1_YEAST | essential | C15H11.9 | WBGene00007617 | x |
| RS11_YEAST | nonessential | F40F11.1 | WBGene00004480 | x |
| RS13_YEAST | essential | C16A3.9 | WBGene00004482 |  |
| RS15_YEAST | essential | F36A2.6 | WBGene00004484 | x |
| RS16_YEAST | nonessential | T01C3.6 | WBGene00004485 | x |
| RS18_YEAST | nonessential | Y57G11C.16 | WBGene00004487 | x |
| RS2_YEAST | essential | C49H3.11 | WBGene00004471 | x |
| RS20_YEAST | essential | Y105E8C.e | WBGene00004489 | x |
| RS21_YEAST | nonessential | F37C12.11 | WBGene00004490 | x |
| RS22_YEAST | nonessential | F53A3.3 | WBGene00004491 | x |
| RS23_YEAST | nonessential | F28D1.7 | WBGene00004492 | x |
| RS24_YEAST | nonessential | T07A9.11 | WBGene00004493 | x |
| RS25_YEAST | nonessential | K02B2.5 | WBGene00004494 |  |
| RS28_YEAST | nonessential | Y41D4A_3613.a | WBGene00004497 |  |
| RS3_YEAST | essential | C23G10.3 | WBGene00004472 | x |
| RS30_YEAST | nonessential | C26F1.4 | WBGene00004499 |  |
| RS37_YEAST | nonessential | H06I04.a | WBGene00006725 | x |
| RS37_YEAST | nonessential | H06I04.f | WBGene00006725 | x |
| RS37_YEAST | nonessential | Y53G8B_1025.a | WBGene00006725 | x |
| RS37_YEAST | nonessential | Y53G8B_1025.b | WBGene00006725 | x |
| RS5_YEAST | essential | T05E11.1 | WBGene00004474 | x |
| RS8_YEAST | nonessential | F42C5.8 | WBGene00004477 | x |
| RS8_YEAST | nonessential | F42C5.1 | WBGene00004477 | x |
| RSD1_YEAST | nonessential | F30A10.6 | WBGene00009264 | x |
| RSMB_YEAST | nonessential | W08E3.1 | WBGene00004915 | x |
| RSP5_YEAST | essential | Y65B4B_10.a | WBGene00007009 |  |
| RSP5_YEAST | essential | Y65B4B_10.e | WBGene00007009 |  |
| RSP5_YEAST | essential | Y65B4B_11.a | WBGene00007009 | x |
| RT04_YEAST | nonessential | T23B12.3 | WBGene00020718 |  |
| RTF1_YEAST | nonessential | F25B3.6 | WBGene00009103 | x |
| RTS2_YEAST | essential | Y52B11A.9 | WBGene00013128 | x |
| RUXE_YEAST | essential | Y49E10.15 | WBGene00004919 | x |
| RUXF_YEAST | essential | ZK652.1 | WBGene00004918 | x |
| RUXG_YEAST | essential | Y71F9B_286.b | WBGene00004920 | x |
| S160_YEAST | nonessential | C08H9.2 | WBGene00007463 |  |
| S3B1_YEAST | essential | T08A11.2 | WBGene00011605 | x |
| SAHH_YEAST | essential | K02F2.2 | WBGene00019322 | x |
| SAR1_YEAST | essential | ZK180.4 | WBGene00022678 | x |
| SC10_YEAST | essential | C33H5.9 | WBGene00016376 |  |
| SC13_YEAST | essential | Y77E11A_3670.c | WBGene00003806 | x |
| SC15_YEAST | essential | C28G1.3 | WBGene00016188 |  |
| SC17_YEAST | essential | D1014.3 | WBGene00017016 |  |
| SC18_YEAST | essential | ZK1014.1 | WBGene00003818 | x |
| SC18_YEAST | essential | H15N14.1 | WBGene00003818 | x |
| SC22_YEAST | nonessential | F55A4.1 | WBGene00018853 |  |
| SC23_YEAST | essential | Y113G7A.3 | WBGene00004754 | x |
| SC62_YEAST | essential | C18E9.2 | WBGene00007683 |  |
| SC65_YEAST | nonessential | F37F2.2 | WBGene00018159 |  |
| SCD6_YEAST | nonessential | Y18D10A.17 | WBGene00012484 |  |
| SCS7_YEAST | nonessential | C25A1.5 | WBGene00007707 | x |
| SEC6_YEAST | essential | F09E5.6 | WBGene00017284 | x |
| SEC7_YEAST | essential | Y87G2A.y | WBGene00012386 | x |
| SEC7_YEAST | essential | Y6B3A.1 | WBGene00012386 | x |
| SED5_YEAST | essential | F55A11.2 | WBGene00006373 | x |
| SEH1_YEAST | nonessential | Y43F4B.4 | WBGene00003804 |  |
| SERB_YEAST | nonessential | Y62E10A.m | WBGene00013379 |  |
| SET2_YEAST | nonessential | Y41D4A_2615.a | WBGene00021515 |  |
| SET2_YEAST | nonessential | Y41D4A_3457.b | WBGene00021515 |  |
| SFT2_YEAST | nonessential | C18E9.10 | WBGene00007690 |  |
| SFT2_YEAST | nonessential | C18E9.4 | WBGene00007690 | x |
| SGPL_YEAST | nonessential | Y66H1B.4 | WBGene00004981 |  |
| SGS1_YEAST | nonessential | T04A11.6 | WBGene00001865 |  |
| SGT1_YEAST | essential | D1054.3 | WBGene00008371 |  |
| SIK1_YEAST | essential | K07C5.4 | WBGene00010627 | x |
| SIN3_YEAST | nonessential | F02E9.4 | WBGene00004117 |  |
| SIS1_YEAST | essential | F54D5.8 | WBGene00001031 |  |
| SKI2_YEAST | nonessential | F01G4.3 | WBGene00008502 |  |
| SLU7_YEAST | essential | K07C5.6 | WBGene00010629 | x |
| SLY1_YEAST | essential | F43D9.3 | WBGene00009654 | x |
| SMC1_YEAST | essential | F28B3.7 | WBGene00001860 | x |
| SMC2_YEAST | essential | M106.1 | WBGene00003367 |  |
| SMC2_YEAST | essential | R06F6.10 | WBGene00003367 | x |
| SMC3_YEAST | essential | Y47D3A.aa | WBGene00004873 |  |
| SMC4_YEAST | essential | F35G12.8 | WBGene00004874 | x |
| SMD1_YEAST | essential | T28D9.10 | WBGene00004916 | x |
| SMD2_YEAST | essential | C52E4.3 | WBGene00004917 | x |
| SMD3_YEAST | essential | Y116A8C.42 | WBGene00004914 | x |
| SMM1_YEAST | nonessential | Y54E5A.6 | WBGene00013201 |  |
| SMP2_YEAST | nonessential | H37A05.1 | WBGene00010425 |  |
| SMT3_YEAST | essential | K12C11.2 | WBGene00004888 | x |
| SNF4_YEAST | nonessential | Y111B2C.h | WBGene00013732 |  |
| SNF5_YEAST | nonessential | R07E5.3 | WBGene00011111 | x |
| SNX3_YEAST | nonessential | W06D4.5 | WBGene00006503 |  |
| SNX4_YEAST | nonessential | Y37A1B.2a | WBGene00003086 |  |
| SNX4_YEAST | nonessential | Y37A1B.3 | WBGene00003086 |  |
| SOF1_YEAST | essential | ZK430.7 | WBGene00022742 | x |
| SOK1_YEAST | nonessential | M05D6.2 | WBGene00010875 |  |
| SP11_YEAST | nonessential | T05E11.4 | WBGene00004985 |  |
| SP14_YEAST | nonessential | C04G6.5 | WBGene00004040 |  |
| SP14_YEAST | nonessential | C04G6.2 | WBGene00004040 |  |
| SP14_YEAST | nonessential | C04G6.3 | WBGene00004040 |  |
| SPB1_YEAST | essential | H06I04.h | WBGene00019168 | x |
| SPC3_YEAST | essential | K12H4.4 | WBGene00019679 | x |
| SPEE_YEAST | nonessential | Y46G5.w | WBGene00012909 |  |
| SPT4_YEAST | nonessential | F54C4.2 | WBGene00005014 |  |
| SPT5_YEAST | essential | K08E4.1 | WBGene00005015 | x |
| SR40_YEAST | nonessential | C25A1.10 | WBGene00000931 |  |
| SR54_YEAST | nonessential | F21D5.7 | WBGene00009012 |  |
| SR68_YEAST | nonessential | F55C5.8 | WBGene00010097 | x |
| SRPR_YEAST | nonessential | F38A1.8 | WBGene00009521 | x |
| SSL1_YEAST | essential | T16H12.4 | WBGene00011814 | x |
| ST20_YEAST | nonessential | C09B8.7 | WBGene00003911 |  |
| ST22_YEAST | nonessential | C09G12.9 | WBGene00015658 |  |
| ST24_YEAST | nonessential | C04F12.10 | WBGene00001405 |  |
| STI1_YEAST | nonessential | R09E12.3 | WBGene00019983 |  |
| STT3_YEAST | essential | T12A2.2 | WBGene00020437 | x |
| SUB2_YEAST | nonessential | C26D10.2 | WBGene00001840 | x |
| SUCB_YEAST | nonessential | F47B10.1 | WBGene00009812 |  |
| SUG2_YEAST | essential | F23F1.8 | WBGene00004504 | x |
| SULX_YEAST | nonessential | F14D12.5 | WBGene00017464 |  |
| SUV3_YEAST | nonessential | C08F8.2 | WBGene00007444 |  |
| SYAC_YEAST | essential | F28H1.3 | WBGene00000197 | x |
| SYC_YEAST | essential | Y23H5A.7 | WBGene00000800 | x |
| SYDC_YEAST | essential | B0464.1 | WBGene00001094 | x |
| SYDM_YEAST | nonessential | F10C2.6 | WBGene00001095 |  |
| SYEM_YEAST | nonessential | T07A9.2 | WBGene00001338 |  |
| SYFA_YEAST | essential | T08B2.9 | WBGene00001497 | x |
| SYFB_YEAST | essential | F22B5.9 | WBGene00001498 | x |
| SYH_YEAST | essential | T11G6.1 | WBGene00002001 | x |
| SYIC_YEAST | essential | R11A8.6 | WBGene00002152 | x |
| SYIM_YEAST | nonessential | C25A1.7 | WBGene00002153 | x |
| SYKC_YEAST | essential | T02G5.9 | WBGene00002238 | x |
| SYLC_YEAST | essential | R74.1 | WBGene00003073 | x |
| SYMC_YEAST | essential | F58B3.5 | WBGene00003415 | x |
| SYNC_YEAST | essential | F22D6.3 | WBGene00003815 | x |
| SYPC_YEAST | nonessential | T27F6.5 | WBGene00004190 |  |
| SYQ_YEAST | essential | Y41E3.4 | WBGene00001336 | x |
| SYSC_YEAST | essential | C47E12.1 | WBGene00005663 | x |
| SYTC_YEAST | essential | C47D12.6 | WBGene00006617 | x |
| SYV_YEAST | nonessential | Y87G2A.l | WBGene00006936 | x |
| SYWC_YEAST | essential | Y80D3A.a | WBGene00006945 | x |
| SYWC_YEAST | essential | Y80D3A.b | WBGene00006945 |  |
| SYWM_YEAST | nonessential | C34E10.4 | WBGene00006946 |  |
| SYYM_YEAST | nonessential | K08F11.4 | WBGene00006968 | x |
| T145_YEAST | essential | W04A8.7 | WBGene00006382 | x |
| T145_YEAST | essential | Y71A12B.a | WBGene00006382 | x |
| T145_YEAST | essential | Y71A12B.b | WBGene00006382 | x |
| T145_YEAST | essential | Y71A12B.c | WBGene00006382 |  |
| T2D4_YEAST | essential | F30F8.8 | WBGene00006386 | x |
| T2D5_YEAST | essential | W09B6.2 | WBGene00006387 |  |
| T2EA_YEAST | essential | ZK550.4 | WBGene00013998 | x |
| T2FA_YEAST | essential | C01F1.1 | WBGene00015296 |  |
| T2FB_YEAST | essential | Y39B6A.f | WBGene00012694 |  |
| TAD2_YEAST | essential | JC8.4 | WBGene00010436 |  |
| TBG_YEAST | essential | F58A4.8 | WBGene00006540 | x |
| TBP_YEAST | essential | T20B12.2 | WBGene00006542 | x |
| TBP7_YEAST | nonessential | F11A10.1 | WBGene00008682 |  |
| TCPA_YEAST | essential | T05C12.7 | WBGene00000377 | x |
| TCPB_YEAST | essential | T21B10.7 | WBGene00011889 | x |
| TCPD_YEAST | essential | K01C8.10 | WBGene00000379 | x |
| TCPE_YEAST | essential | C07G2.3 | WBGene00000380 | x |
| TCPG_YEAST | essential | F54A3_31.e | WBGene00018782 | x |
| TCPH_YEAST | essential | T10B5.5 | WBGene00020391 | x |
| TCPQ_YEAST | essential | Y55F3A_750.c | WBGene00021934 | x |
| TCPQ_YEAST | essential | Y55F3A_750.d | WBGene00021934 | x |
| TCPZ_YEAST | essential | F01F1.8 | WBGene00000381 | x |
| TCTP_YEAST | nonessential | F25H2.11 | WBGene00009122 | x |
| TEL1_YEAST | nonessential | Y48G1C_55.a | WBGene00000227 |  |
| TF3B_YEAST | essential | F45E12.2 | WBGene00000271 | x |
| TFC5_YEAST | essential | B0261.1 | WBGene00015091 | x |
| TFS2_YEAST | nonessential | T24H10.1 | WBGene00012000 |  |
| TLG2_YEAST | nonessential | ZC155.7 | WBGene00022534 |  |
| TOP1_YEAST | nonessential | M01E5.5 | WBGene00006595 |  |
| TPIS_YEAST | essential | Y17G7B.7 | WBGene00006601 |  |
| TR20_YEAST | essential | W05H7.3 | WBGene00021046 |  |
| TRM8_YEAST | nonessential | W02B12.10 | WBGene00012205 |  |
| TRMU_YEAST | nonessential | B0035.16 | WBGene00007114 |  |
| TWF1_YEAST | nonessential | F38E9.5 | WBGene00018187 |  |
| TXTP_YEAST | nonessential | K11H3.3 | WBGene00010780 |  |
| TYDP_YEAST | nonessential | F52C12.1 | WBGene00018678 |  |
| TYSY_YEAST | essential | Y110A7A.q | WBGene00022455 |  |
| UBA1_YEAST | essential | C47E12.5 | WBGene00006699 |  |
| UBA2_YEAST | essential | W02A11.4 | WBGene00006700 |  |
| UBC2_YEAST | nonessential | C35B1.1 | WBGene00006701 |  |
| UBC3_YEAST | essential | Y71G12A_187.a | WBGene00006702 |  |
| UBC9_YEAST | essential | F29B9.6 | WBGene00006706 |  |
| UBPE_YEAST | nonessential | T27A3.2 | WBGene00020839 |  |
| UBR1_YEAST | nonessential | C32E8.11 | WBGene00016326 |  |
| UCR7_YEAST | nonessential | T02H6.11 | WBGene00020181 |  |
| UDPG_YEAST | essential | K08E3.5 | WBGene00010665 | x |
| UFD1_YEAST | essential | F19B6.2 | WBGene00006733 | x |
| UFD2_YEAST | nonessential | T05H10.5 | WBGene00006734 |  |
| UGA2_YEAST | nonessential | F45H10.1 | WBGene00000113 |  |
| ULP1_YEAST | essential | T10F2.3 | WBGene00006736 |  |
| UME3_YEAST | nonessential | H14E04.5 | WBGene00000506 |  |
| UME5_YEAST | nonessential | F39H11.3 | WBGene00000409 |  |
| UNG_YEAST | nonessential | Y56A3A.29 | WBGene00013241 |  |
| UT11_YEAST | essential | C16C10.2 | WBGene00007623 | x |
| UTR4_YEAST | nonessential | F58H1.3 | WBGene00010286 |  |
| VATC_YEAST | nonessential | Y38F2A_5743.f | WBGene00006920 |  |
| VATD_YEAST | nonessential | F55H2.2 | WBGene00010130 |  |
| VATE_YEAST | nonessential | C17H12.14 | WBGene00006917 | x |
| VATF_YEAST | nonessential | ZK970.4 | WBGene00006918 | x |
| VATG_YEAST | nonessential | F46F11.5 | WBGene00006919 | x |
| VATO_YEAST | nonessential | T01H3.1 | WBGene00011347 | x |
| VP13_YEAST | nonessential | T08G11.1 | WBGene00011629 |  |
| VP15_YEAST | nonessential | ZK930.1 | WBGene00014151 |  |
| VP15_YEAST | nonessential | ZK930.7 | WBGene00014151 |  |
| VP16_YEAST | nonessential | C05D11.2 | WBGene00006516 |  |
| VP26_YEAST | nonessential | T20D3.7 | WBGene00006931 |  |
| VP27_YEAST | nonessential | C07G1.5 | WBGene00004101 | x |
| VP28_YEAST | nonessential | Y87G2A.s | WBGene00013598 | x |
| VP34_YEAST | nonessential | B0025.1 | WBGene00006932 |  |
| VP35_YEAST | nonessential | F59G1.3 | WBGene00006933 |  |
| VP45_YEAST | nonessential | C44C1.1 | WBGene00016643 |  |
| VP45_YEAST | nonessential | C44C1.4 | WBGene00016643 |  |
| VPS4_YEAST | nonessential | Y34D9A_152.a | WBGene00021334 | x |
| VPS5_YEAST | nonessential | C05D9.1 | WBGene00004927 |  |
| VPS9_YEAST | nonessential | Y39A1A.5 | WBGene00012644 |  |
| WEB1_YEAST | essential | T01G1.3 | WBGene00011338 |  |
| XPO1_YEAST | essential | ZK742.1 | WBGene00002078 | x |
| Y08L_YEAST | nonessential | R166.3 | WBGene00011303 |  |
| YAB9_YEAST | nonessential | M03C11.8 | WBGene00010845 |  |
| YAD2_YEAST | essential | T27F2.1 | WBGene00004806 | x |
| YAD6_YEAST | nonessential | Y47D3A.gg | WBGene00012126 |  |
| YAD6_YEAST | nonessential | T28D6.6 | WBGene00012126 |  |
| YAE2_YEAST | nonessential | K09E9.2 | WBGene00010725 |  |
| YAE6_YEAST | nonessential | Y105E8C.j | WBGene00013671 |  |
| YAH3_YEAST | nonessential | F21H12.1 | WBGene00017683 |  |
| YAK1_YEAST | nonessential | T04C10.1 | WBGene00003149 |  |
| YB78_YEAST | nonessential | F56A3.2 | WBGene00018909 |  |
| YB85_YEAST | nonessential | T04B8.5 | WBGene00020207 |  |
| YBA4_YEAST | essential | F18C5.3 | WBGene00017558 |  |
| YBD6_YEAST | nonessential | F09E5.8 | WBGene00017286 |  |
| YBF5_YEAST | nonessential | CD4.2 | WBGene00000795 |  |
| YBF7_YEAST | nonessential | C24G6.8 | WBGene00016062 |  |
| YBN5_YEAST | nonessential | W08E3.3 | WBGene00012344 |  |
| YBS0_YEAST | essential | M02B7.4 | WBGene00019725 |  |
| YBV8_YEAST | nonessential | C37A2.2 | WBGene00004110 |  |
| YBY3_YEAST | nonessential | C34E10.5 | WBGene00016408 |  |
| YCE5_YEAST | nonessential | H17B01.4 | WBGene00019209 |  |
| YCF9_YEAST | essential | C05C8.2 | WBGene00015461 | x |
| YCT4_YEAST | nonessential | R01B10.4 | WBGene00019806 |  |
| YCT7_YEAST | nonessential | C27F2.4 | WBGene00016166 |  |
| YCU1_YEAST | nonessential | F34D10.6 | WBGene00009376 |  |
| YCU9_YEAST | nonessential | Y52B11A.2 | WBGene00013122 |  |
| YCW2_YEAST | essential | W07E6.2 | WBGene00021074 | x |
| YD13_YEAST | essential | R53.6 | WBGene00011275 | x |
| YD23_YEAST | nonessential | ZK632.10 | WBGene00014017 |  |
| YD61_YEAST | nonessential | T07A9.8 | WBGene00020296 |  |
| YD66_YEAST | nonessential | C34D4.4 | WBGene00016400 |  |
| YD83_YEAST | nonessential | C05D11.3 | WBGene00015482 |  |
| YDAK_YEAST | nonessential | F09F7.4 | WBGene00017301 |  |
| YDB6_YEAST | essential | E02H1.6 | WBGene00008458 |  |
| YEC0_YEAST | nonessential | B0334.3 | WBGene00007143 |  |
| YEJ4_YEAST | nonessential | C33F10.3 | WBGene00016353 |  |
| YEJ6_YEAST | nonessential | K01C8.9 | WBGene00003821 | x |
| YEM6_YEAST | essential | Y76A2A.1 | WBGene00012097 |  |
| YEM6_YEAST | essential | T27E9.7 | WBGene00012097 |  |
| YEO1_YEAST | nonessential | T26A5.5 | WBGene00020821 |  |
| YEQ8_YEAST | nonessential | R119.2 | WBGene00020088 |  |
| YER2_YEAST | nonessential | F28D1.1 | WBGene00009211 | x |
| YET7_YEAST | nonessential | F10G8.3 | WBGene00003803 | x |
| YEV6_YEAST | essential | W09C5.1 | WBGene00012351 |  |
| YEX0_YEAST | nonessential | F26F2.7 | WBGene00009172 |  |
| YEY6_YEAST | nonessential | K08H10.8 | WBGene00007787 |  |
| YEZ3_YEAST | nonessential | F22F7.7 | WBGene00017724 |  |
| YFD0_YEAST | nonessential | T14D7.1 | WBGene00011767 |  |
| YFH5_YEAST | essential | F09D1.1 | WBGene00017280 | x |
| YFH6_YEAST | nonessential | K12C11.1 | WBGene00019673 |  |
| YFI8_YEAST | nonessential | H27A22.1 | WBGene00010418 |  |
| YG12_YEAST | essential | Y105E8C.d | WBGene00013676 | x |
| YG1D_YEAST | nonessential | Y50D7_165.b | WBGene00021757 |  |
| YG1D_YEAST | nonessential | Y50D7_165.d | WBGene00021757 |  |
| YG1W_YEAST | essential | Y71F9B_275.b | WBGene00022126 |  |
| YG1W_YEAST | essential | Y71F9B_297.d | WBGene00022126 |  |
| YG22_YEAST | nonessential | E04D5.1 | WBGene00008480 |  |
| YG25_YEAST | nonessential | M04F3.4 | WBGene00019770 |  |
| YG2M_YEAST | nonessential | F17A9.2 | WBGene00017534 |  |
| YG2M_YEAST | nonessential | F17A9.3 | WBGene00017534 |  |
| YG2O_YEAST | nonessential | C42C1.10 | WBGene00016588 |  |
| YG3J_YEAST | essential | F32E10.1 | WBGene00017989 | x |
| YG3Y_YEAST | nonessential | C02F5.2 | WBGene00015346 | x |
| YG4W_YEAST | nonessential | T24H7.1 | WBGene00004015 |  |
| YG58_YEAST | essential | Y39B6B.a | WBGene00012676 | x |
| YG5F_YEAST | nonessential | C16C10.1 | WBGene00007622 |  |
| YG5O_YEAST | nonessential | Y54E2A.6 | WBGene00013191 |  |
| YG5O_YEAST | nonessential | Y54E2A.7 | WBGene00013191 |  |
| YG5U_YEAST | essential | F33A8.1 | WBGene00002957 | x |
| YG5Y_YEAST | nonessential | C54H2.5 | WBGene00004788 | x |
| YGA4_YEAST | nonessential | Y39G10A_246.b | WBGene00021467 |  |
| YGB8_YEAST | essential | K08D10.2 | WBGene00001033 |  |
| YGE7_YEAST | essential | R10D12.12 | WBGene00011193 |  |
| YGG8_YEAST | essential | W09D10.3 | WBGene00012361 |  |
| YGJ9_YEAST | nonessential | C53H9.2 | WBGene00016907 | x |
| YGL1_YEAST | essential | T06E6.1 | WBGene00011538 | x |
| YGO2_YEAST | essential | T27F7.3 | WBGene00020868 | x |
| YGR1_YEAST | nonessential | Y25C1A.7a | WBGene00021294 |  |
| YGS4_YEAST | nonessential | R08D7.1 | WBGene00011142 | x |
| YGW1_YEAST | nonessential | F29C4.6 | WBGene00017928 |  |
| YH04_YEAST | nonessential | C47E12.3 | WBGene00008148 |  |
| YHA2_YEAST | nonessential | C34G6.7 | WBGene00004109 |  |
| YHB3_YEAST | nonessential | F21D5.2 | WBGene00009007 |  |
| YHG4_YEAST | nonessential | F45E12.1 | WBGene00018474 |  |
| YHH1_YEAST | nonessential | W03B1.4 | WBGene00005662 |  |
| YHI0_YEAST | essential | T20H4.3 | WBGene00004189 | x |
| YHN0_YEAST | essential | C53A5.2 | WBGene00008263 |  |
| YHN6_YEAST | nonessential | W09D10.4 | WBGene00012362 |  |
| YHP9_YEAST | essential | C47D12.1 | WBGene00007028 | x |
| YHR1_YEAST | nonessential | F42G8.6 | WBGene00018357 |  |
| YHW8_YEAST | nonessential | M01E5.2 | WBGene00010805 |  |
| YHY6_YEAST | nonessential | C10C5.6 | WBGene00007510 |  |
| YHY8_YEAST | essential | F17C11.7 | WBGene00008918 |  |
| YIE2_YEAST | nonessential | ZK370.5 | WBGene00022719 |  |
| YIE4_YEAST | nonessential | Y17G7B.15 | WBGene00000565 |  |
| YIG4_YEAST | nonessential | F29B9.1 | WBGene00017919 |  |
| YII3_YEAST | essential | Y71H2_388.b | WBGene00022171 |  |
| YIJ1_YEAST | essential | Y41C4A.9 | WBGene00012756 |  |
| YIJ7_YEAST | nonessential | W09G3.2 | WBGene00012366 |  |
| YIK3_YEAST | nonessential | C14B1.5 | WBGene00007576 |  |
| YIK4_YEAST | essential | Y48A5A.1 | WBGene00021655 |  |
| YIL0_YEAST | nonessential | K01A11.2 | WBGene00010450 |  |
| YIL3_YEAST | nonessential | F08B1.1 | WBGene00006923 | x |
| YIS5_YEAST | nonessential | C30B5.4 | WBGene00016245 |  |
| YJ14_YEAST | nonessential | C30B5.2 | WBGene00016244 |  |
| YJ40_YEAST | nonessential | C14A4.1 | WBGene00007555 |  |
| YJ42_YEAST | essential | C34E10.2 | WBGene00001661 | x |
| YJ54_YEAST | nonessential | C27F2.7 | WBGene00016171 |  |
| YJ72_YEAST | nonessential | W02A11.2 | WBGene00012193 |  |
| YJ76_YEAST | nonessential | C07A9.4 | WBGene00003571 |  |
| YJ80_YEAST | nonessential | F53A2.8 | WBGene00003478 |  |
| YJ89_YEAST | nonessential | ZK593.4 | WBGene00004319 |  |
| YJ95_YEAST | nonessential | C34E11.1 | WBGene00004682 |  |
| YJ99_YEAST | nonessential | R08D7.4 | WBGene00011148 |  |
| YJE6_YEAST | nonessential | C45G3.3 | WBGene00001590 |  |
| YJG2_YEAST | nonessential | F28C6.4 | WBGene00009204 |  |
| YJG8_YEAST | nonessential | Y48G10B.b | WBGene00013018 |  |
| YJJ1_YEAST | essential | Y110A2A_54.a | WBGene00022447 |  |
| YJJ1_YEAST | essential | Y110A2A_1898.d | WBGene00022447 |  |
| YJJ1_YEAST | essential | Y110A2A_1898.e | WBGene00022447 |  |
| YJJ7_YEAST | essential | T15B7.2 | WBGene00020517 |  |
| YJK0_YEAST | nonessential | C56A3.8 | WBGene00008346 |  |
| YJK9_YEAST | essential | ZK430.1 | WBGene00022739 | x |
| YJK9_YEAST | essential | T12C9.2 | WBGene00022739 | x |
| YJU1_YEAST | nonessential | W02B12.8 | WBGene00012203 |  |
| YJX8_YEAST | nonessential | C37C3.8 | WBGene00016500 |  |
| YJY3_YEAST | essential | B0491.1 | WBGene00007189 |  |
| YJZ4_YEAST | nonessential | ZC373.5 | WBGene00013870 |  |
| YK10_YEAST | nonessential | T07A5.2 | WBGene00011555 |  |
| YK18_YEAST | essential | Y71H2_385.b | WBGene00022166 |  |
| YK31_YEAST | nonessential | F40E10.6 | WBGene00009574 |  |
| YK50_YEAST | nonessential | K02B2.2 | WBGene00019255 |  |
| YK59_YEAST | essential | E04A4.4 | WBGene00001983 |  |
| YKA2_YEAST | nonessential | Y46G5.m | WBGene00012903 |  |
| YKA9_YEAST | nonessential | F10E7.5 | WBGene00017347 |  |
| YKE1_YEAST | nonessential | T27F7.1 | WBGene00020866 |  |
| YKE1_YEAST | nonessential | F59A6.7 | WBGene00020866 |  |
| YKF4_YEAST | nonessential | F47A4.2 | WBGene00001081 |  |
| YKF9_YEAST | essential | F36F2.3 | WBGene00009477 | x |
| YKI2_YEAST | essential | ZK546.14 | WBGene00022765 | x |
| YKJ5_YEAST | essential | F37C12.14 | WBGene00018149 |  |
| YKL7_YEAST | nonessential | ZC395.10 | WBGene00022599 |  |
| YKP1_YEAST | nonessential | R107.2 | WBGene00011298 |  |
| YKQ0_YEAST | nonessential | Y54G11A.11 | WBGene00013219 |  |
| YKQ5_YEAST | essential | M01B12.2 | WBGene00021840 |  |
| YKQ5_YEAST | essential | Y54E10B_159.a | WBGene00021840 |  |
| YKT6_YEAST | essential | B0361.10 | WBGene00015164 |  |
| YKV5_YEAST | nonessential | Y38F2A_6126.b | WBGene00021429 |  |
| YL01_YEAST | nonessential | Y37E11B.5 | WBGene00021377 |  |
| YL05_YEAST | nonessential | C45G9.2 | WBGene00016674 |  |
| YL09_YEAST | essential | Y45F10D.8 | WBGene00012887 | x |
| YL16_YEAST | nonessential | C32E8.5 | WBGene00016323 | x |
| YL22_YEAST | essential | W06E11.4 | WBGene00021063 |  |
| YL27_YEAST | nonessential | Y39B6B.ee | WBGene00012666 | x |
| YL34_YEAST | essential | Y48C3A.i | WBGene00003119 |  |
| YL51_YEAST | nonessential | Y87G2A.b | WBGene00003585 |  |
| YL53_YEAST | nonessential | D2023.6 | WBGene00008410 |  |
| YL86_YEAST | nonessential | K04G2.6 | WBGene00010565 |  |
| YM62_YEAST | nonessential | C32D5.3 | WBGene00016311 |  |
| YM8L_YEAST | nonessential | Y43F4B.5 | WBGene00012803 |  |
| YM8T_YEAST | nonessential | F41C3.4 | WBGene00018270 | x |
| YMB4_YEAST | nonessential | C35D10.12 | WBGene00016448 |  |
| YMD6_YEAST | nonessential | W03F8.4 | WBGene00020994 |  |
| YME1_YEAST | nonessential | M03C11.5 | WBGene00010842 |  |
| YME9_YEAST | nonessential | K02F2.3 | WBGene00019323 | x |
| YMI0_YEAST | nonessential | F36A2.2 | WBGene00009452 |  |
| YMJ3_YEAST | essential | C24A11.9 | WBGene00017855 |  |
| YMJ3_YEAST | essential | F27C1.6 | WBGene00017855 | x |
| YMJ6_YEAST | nonessential | M18.3 | WBGene00010889 |  |
| YMN1_YEAST | nonessential | M106.3 | WBGene00010911 |  |
| YMO2_YEAST | nonessential | C16C10.11 | WBGene00007630 |  |
| YMO9_YEAST | nonessential | F42F12.4 | WBGene00009636 |  |
| YMT1_YEAST | nonessential | F37C12.12 | WBGene00003176 |  |
| YMT8_YEAST | nonessential | F23C8.9 | WBGene00017738 |  |
| YMT9_YEAST | essential | Y48B6A.1 | WBGene00012978 | x |
| YMW7_YEAST | nonessential | Y65B4A_182.b | WBGene00022027 |  |
| YMW7_YEAST | nonessential | Y65B4A_182.c | WBGene00022027 | x |
| YN03_YEAST | essential | F57A8.2 | WBGene00010178 |  |
| YN15_YEAST | nonessential | F52B5.1 | WBGene00009920 |  |
| YN26_YEAST | nonessential | Y65B4A_174.a | WBGene00022025 |  |
| YN26_YEAST | nonessential | Y65B4A_174.b | WBGene00022025 |  |
| YN26_YEAST | nonessential | Y65B4A_179.b | WBGene00022025 |  |
| YN28_YEAST | nonessential | C26E6.3 | WBGene00016139 |  |
| YN48_YEAST | essential | F54C9.9 | WBGene00010044 | x |
| YN53_YEAST | essential | T20B12.1 | WBGene00020600 | x |
| YN65_YEAST | nonessential | C34B7.2 | WBGene00007912 |  |
| YN8K_YEAST | nonessential | T03D8.2 | WBGene00011391 |  |
| YN8Q_YEAST | essential | C04H5.1 | WBGene00007312 |  |
| YN8U_YEAST | essential | T19A6.2 | WBGene00003596 | x |
| YN8V_YEAST | essential | F57B10.8 | WBGene00019005 |  |
| YNA6_YEAST | essential | C10H11.8 | WBGene00015697 |  |
| YNC2_YEAST | nonessential | Y53F4B.e | WBGene00013151 |  |
| YNC2_YEAST | nonessential | Y53F4B.f | WBGene00013151 |  |
| YNC3_YEAST | nonessential | C16A3.7 | WBGene00015812 |  |
| YNC6_YEAST | essential | C34E10.1 | WBGene00001662 |  |
| YNC7_YEAST | nonessential | C27C12.2 | WBGene00007772 |  |
| YNK7_YEAST | nonessential | M04B2.3 | WBGene00001585 |  |
| YNL0_YEAST | essential | T04A8.6 | WBGene00011408 | x |
| YNM9_YEAST | nonessential | T27A3.6 | WBGene00020842 |  |
| YNN2_YEAST | essential | F55A12.8 | WBGene00018866 | x |
| YNQ8_YEAST | nonessential | ZK688.3 | WBGene00022798 |  |
| YNR5_YEAST | nonessential | B0035.12 | WBGene00007111 | x |
| YNU1_YEAST | nonessential | F41E6.3 | WBGene00018285 | x |
| YNU1_YEAST | nonessential | F41E6.4 | WBGene00018285 |  |
| YNU1_YEAST | nonessential | F41E6.10 | WBGene00018285 |  |
| YNW7_YEAST | nonessential | T03F6.2 | WBGene00001035 |  |
| YNZ3_YEAST | nonessential | F32H2.4 | WBGene00009341 |  |
| YNZ5_YEAST | nonessential | K08D12.h | WBGene00019537 |  |
| YNZ5_YEAST | nonessential | K08D12.c | WBGene00019537 |  |
| YO06_YEAST | nonessential | F52C12.2 | WBGene00018679 |  |
| YO26_YEAST | essential | C07E3.2 | WBGene00007413 | x |
| YO7T_YEAST | nonessential | B0024.11 | WBGene00007101 |  |
| YOJ8_YEAST | nonessential | C05D11.1 | WBGene00015481 |  |
| YOZ1_YEAST | nonessential | C54G4.6 | WBGene00008316 |  |
| YP18_YEAST | nonessential | C01G10.9 | WBGene00007236 |  |
| YP46_YEAST | essential | Y39B6B.o | WBGene00012692 | x |
| YP59_YEAST | nonessential | Y49E10.2 | WBGene00013029 |  |
| YP67_YEAST | nonessential | Y54G11A.9 | WBGene00013218 |  |
| YPT1_YEAST | essential | C39F7.4 | WBGene00004266 | x |
| YPT7_YEAST | nonessential | W03C9.3 | WBGene00004271 | x |
| YRB1_YEAST | essential | F59A2.1 | WBGene00003795 | x |
| YTM1_YEAST | essential | F55F8.5 | WBGene00018893 | x |
| ZPR1_YEAST | essential | W03F9.1 | WBGene00020999 |  |
| ZUO1_YEAST | nonessential | F38A5.13 | WBGene00001029 |  |
