# Supplementary material for: Combinatorial RNA interference in Caenorhabditis elegans reveals that redundancy between gene duplicates can be maintained for more than 80 million years of evolution
Source: Genome Biol. 2006 Aug 2;7(8):R69. doi: 10.1186/gb-2006-7-8-r69 (PMC1779603; doi:10.1186/gb-2006-7-8-r69)
Supplement: Additional data file 4 — A Word document presenting C. elegans duplicate gene pairs included in this study, their orthologous genes in C. briggsae, and levels of protein identity between C. elegans gene duplicates, as well as Ka and Ks values between duplicate gene pairs. [file gb-2006-7-8-r69-S4.doc]

*C. elegans* duplicate gene pairs. Shown are *C. elegans* Wormbase gene names (‘CE_WBGeneName’), the corresponding orthologous genes in *C. briggsae* (‘CB_WBGeneName’), percentages protein identity between *C. elegans* gene duplicates (‘Prot_id’), protein lengths (‘Length’), as well as Ka and Ks values of *C. elegans* duplicates.

| **CE_WBGeneName** | **CB_WBGeneName** | **Prot_id** | **Length** | **Ka** | **Ks** |
| --- | --- | --- | --- | --- | --- |
| WBGene00008354 | WBGene00029517 | 69.86 | 148 | 0.2333 | 13.6919 |
| WBGene00009918 | WBGene00032946 | 69.86 | 146 | 0.2333 | 13.6919 |
| WBGene00008956 | WBGene00023610 | 32.2 | 302 | 0.7607 | 3.4968 |
| WBGene00021461 | WBGene00026730 | 32.2 | 998 | 0.7607 | 3.4968 |
| WBGene00016195 | WBGene00030711 | 68.08 | 213 | 0.2592 | 14.5671 |
| WBGene00001331 | WBGene00036885 | 68.08 | 213 | 0.2592 | 14.5671 |
| WBGene00011089 | WBGene00041735 | 41.69 | 461 | 0.5601 | 7.3706 |
| WBGene00011088 | WBGene00041735 | 41.69 | 464 | 0.5601 | 7.3706 |
| WBGene00017012 | WBGene00023673 | 50.93 | 655 | 0.4403 | 18.7674 |
| WBGene00009218 | WBGene00026207 | 50.93 | 650 | 0.4403 | 18.7674 |
| WBGene00022162 | WBGene00033086 | 54.44 | 118 | 0.4439 | 18.9282 |
| WBGene00001051 | WBGene00035798 | 54.44 | 94 | 0.4439 | 18.9282 |
| WBGene00016849 | WBGene00035798 | 69.7 | 517 | 0.1896 | 2.8373 |
| WBGene00018269 | WBGene00039574 | 69.7 | 505 | 0.1896 | 2.8373 |
| WBGene00008275 | WBGene00040435 | 40.51 | 318 | 0.6291 | 5.0746 |
| WBGene00017480 | WBGene00027856 | 40.51 | 324 | 0.6291 | 5.0746 |
| WBGene00003036 | WBGene00026512 | 54.79 | 417 | 0.3672 | 20.8239 |
| WBGene00004312 | WBGene00026513 | 54.79 | 412 | 0.3672 | 20.8239 |
| WBGene00003254 | WBGene00035179 | 45.45 | 552 | 0.5146 | 16.3993 |
| WBGene00016380 | WBGene00037242 | 45.45 | 485 | 0.5146 | 16.3993 |
| WBGene00003902 | WBGene00025293 | 66.3 | 646 | 0.2681 | 11.5863 |
| WBGene00003903 | WBGene00029486 | 66.3 | 692 | 0.2681 | 11.5863 |
| WBGene00017298 | WBGene00036366 | 31.66 | 592 | 0.8886 | 15.0256 |
| WBGene00010663 | WBGene00037717 | 31.66 | 608 | 0.8886 | 15.0256 |
| WBGene00016728 | WBGene00032079 | 52.23 | 408 | 0.3683 | 18.8067 |
| WBGene00007254 | WBGene00040600 | 52.23 | 423 | 0.3683 | 18.8067 |
| WBGene00010398 | WBGene00029678 | n.d. | 462 | n.d. | n.d. |
| WBGene00012129 | WBGene00023775 | n.d. | 393 | n.d. | n.d. |
| WBGene00000205 | WBGene00026926 | 50 | 152 | 0.3845 | 2.7613 |
| WBGene00016729 | WBGene00032071 | 50 | 146 | 0.3845 | 2.7613 |
| WBGene00001425 | WBGene00041223 | 29.71 | 138 | 0.8693 | 10.5654 |
| WBGene00001424 | WBGene00032315 | 29.71 | 143 | 0.8693 | 10.5654 |
| WBGene00012031 | WBGene00032933 | 46.35 | 722 | 0.4574 | 16.9005 |
| WBGene00013049 | WBGene00027197 | 46.35 | 673 | 0.4574 | 16.9005 |
| WBGene00004386 | WBGene00034641 | 52.45 | 217 | 0.364 | 17.6389 |
| WBGene00004385 | WBGene00034640 | 52.45 | 206 | 0.364 | 17.6389 |
| WBGene00019435 | WBGene00025033 | 34.72 | 1032 | 1.1586 | 9.1 |
| WBGene00019146 | WBGene00025033 | 34.72 | 1275 | 1.1586 | 9.1 |
| WBGene00016374 | WBGene00037234 | 37.96 | 326 | 0.6334 | 18.25 |
| WBGene00016373 | WBGene00037235 | 37.96 | 248 | 0.6334 | 18.25 |
| WBGene00007812 | WBGene00027801 | 81.15 | 191 | 0.1132 | 2.9571 |
| WBGene00009575 | WBGene00025918 | 81.15 | 191 | 0.1132 | 2.9571 |
| WBGene00013140 | WBGene00024343 | 71.01 | 338 | 0.1961 | 16.8103 |
| WBGene00019827 | WBGene00034868 | 71.01 | 634 | 0.1961 | 16.8103 |
| WBGene00011637 | WBGene00026186 | 43.53 | 326 | 0.6258 | 13.1503 |
| WBGene00015516 | WBGene00033815 | 43.53 | 349 | 0.6258 | 13.1503 |
| WBGene00021787 | WBGene00029321 | 52.51 | 448 | 0.4291 | 11.2562 |
| WBGene00010456 | WBGene00024026 | 52.51 | 499 | 0.4291 | 11.2562 |
| WBGene00001253 | WBGene00034303 | 46.25 | 367 | 0.5319 | 6.895 |
| WBGene00001186 | WBGene00034302 | 46.25 | 376 | 0.5319 | 6.8952 |
| WBGene00001646 | WBGene00031144 | 51.88 | 165 | 0.3872 | 9.2763 |
| WBGene00001647 | WBGene00038538 | 51.88 | 347 | 0.3872 | 9.2763 |
| WBGene00004272 | WBGene00033040 | 65.5 | 211 | 0.2688 | 19.587 |
| WBGene00004273 | WBGene00033549 | 65.5 | 201 | 0.2688 | 19.587 |
| WBGene00000591 | WBGene00036571 | 30.82 | 652 | 0.8685 | 8.633 |
| WBGene00004737 | WBGene00027003 | 30.82 | 645 | 0.8685 | 8.633 |
| WBGene00007729 | WBGene00026215 | 30.54 | 708 | 1.0366 | 10.8319 |
| WBGene00008203 | WBGene00023788 | 30.54 | 481 | 1.0366 | 10.8319 |
| WBGene00011543 | WBGene00027436 | 32.82 | 282 | 0.7472 | 9.4114 |
| WBGene00010339 | WBGene00036088 | 32.82 | 262 | 0.7472 | 9.4114 |
| WBGene00000836 | WBGene00037818 | 50.21 | 780 | 0.4082 | 6.3726 |
| WBGene00000841 | WBGene00037818 | 50.21 | 729 | 0.4082 | 6.3726 |
| WBGene00003825 | WBGene00026485 | 19.18 | 444 | 1.2472 | 3.4483 |
| WBGene00006499 | WBGene00038506 | 19.18 | 497 | 1.2472 | 3.4483 |
| WBGene00010558 | WBGene00026268 | 24.89 | 1156 | 0.9815 | 13.4681 |
| WBGene00004215 | WBGene00025942 | 24.89 | 2180 | 0.9815 | 13.4681 |
| WBGene00021260 | WBGene00032812 | 34.16 | 323 | 0.7718 | 10.173 |
| WBGene00009262 | WBGene00042593 | 34.16 | 332 | 0.7718 | 10.173 |
| WBGene00018398 | WBGene00032511 | 48.05 | 558 | 0.4977 | 16.5779 |
| WBGene00011932 | WBGene00032560 | 48.05 | 512 | 0.4977 | 16.5779 |
| WBGene00021956 | WBGene00038350 | 67.19 | 459 | 0.23 | 11.2965 |
| WBGene00011250 | WBGene00027221 | 67.19 | 442 | 0.23 | 11.2965 |
| WBGene00006757 | WBGene00036613 | 38.66 | 591 | 0.5817 | 14.5888 |
| WBGene00020298 | WBGene00034298 | 38.66 | 562 | 0.5817 | 14.5888 |
| WBGene00006715 | WBGene00035871 | 53.27 | 199 | 0.3782 | 14.8893 |
| WBGene00006716 | WBGene00035728 | 53.27 | 260 | 0.3782 | 14.8893 |
| WBGene00013957 | WBGene00042950 | 70.11 | 383 | 0.1913 | 2.9108 |
| WBGene00009462 | WBGene00042950 | 70.11 | 359 | 0.1913 | 2.9108 |
| WBGene00003563 | WBGene00029945 | 64.02 | 191 | 0.2498 | 7.1603 |
| WBGene00003565 | WBGene00029932 | 64.02 | 199 | 0.2498 | 7.1603 |
| WBGene00017436 | WBGene00025632 | 38.81 | 374 | 0.7092 | 5.8279 |
| WBGene00012834 | WBGene00041901 | 38.81 | 338 | 0.7092 | 5.8279 |
| WBGene00017673 | WBGene00041901 | 71.33 | 295 | 0.1847 | 1.2764 |
| WBGene00019710 | WBGene00035270 | 71.33 | 293 | 0.1847 | 1.2764 |
| WBGene00020109 | WBGene00023959 | n.d. | 227 | n.d. | n.d. |
| WBGene00009111 | WBGene00025286 | n.d. | 245 | n.d. | n.d. |
| WBGene00020142 | WBGene00036253 | 51.18 | 624 | 0.4313 | 10.0446 |
| WBGene00019801 | WBGene00029106 | 51.18 | 606 | 0.4313 | 10.0446 |
| WBGene00009902 | WBGene00024008 | 59.11 | 269 | 0.3049 | 2.5368 |
| WBGene00009903 | WBGene00024007 | 59.11 | 286 | 0.3049 | 2.5368 |
| WBGene00010984 | WBGene00029668 | 62.74 | 441 | 0.2636 | 16.7168 |
| WBGene00009232 | WBGene00029668 | 62.74 | 437 | 0.2636 | 16.7168 |
| WBGene00022456 | WBGene00032099 | 63.82 | 450 | 0.2643 | 16.2274 |
| WBGene00019295 | WBGene00031037 | 63.82 | 457 | 0.2643 | 16.2274 |
| WBGene00006938 | WBGene00031037 | 35.42 | 468 | 0.6464 | 15.7517 |
| WBGene00006940 | WBGene00024344 | 35.42 | 677 | 0.6464 | 15.7517 |
| WBGene00004930 | WBGene00032360 | 68.54 | 180 | 0.2087 | 1.1986 |
| WBGene00007036 | WBGene00032360 | 68.54 | 178 | 0.2087 | 1.1986 |
| WBGene00020082 | WBGene00030030 | 38.15 | 770 | 0.654 | 19.5873 |
| WBGene00007954 | WBGene00036974 | 38.15 | 745 | 0.654 | 19.5873 |
| WBGene00016652 | WBGene00040842 | 66.18 | 419 | 0.2383 | 16.2499 |
| WBGene00015778 | WBGene00027571 | 66.18 | 414 | 0.2383 | 16.2499 |
| WBGene00004269 | WBGene00029059 | 78.92 | 205 | 0.134 | 16.4867 |
| WBGene00004270 | WBGene00036043 | 78.92 | 205 | 0.134 | 16.4867 |
| WBGene00011404 | WBGene00041573 | 20.39 | 368 | 1.0782 | 11.4318 |
| WBGene00007925 | WBGene00031308 | 20.39 | 392 | 1.0782 | 11.4318 |
| WBGene00022718 | WBGene00029118 | 46.64 | 1342 | 0.4657 | 18.6434 |
| WBGene00010915 | WBGene00024017 | 46.64 | 880 | 0.4657 | 18.6434 |
| WBGene00003845 | WBGene00036484 | 43.72 | 242 | 0.5872 | 3.2719 |
| WBGene00003846 | WBGene00035233 | 43.72 | 254 | 0.5872 | 3.2719 |
| WBGene00006810 | WBGene00034778 | 69.12 | 611 | 0.2181 | 12.5771 |
| WBGene00010685 | WBGene00041133 | 69.12 | 600 | 0.2181 | 12.5771 |
| WBGene00000110 | WBGene00038231 | 76.73 | 493 | 0.1493 | 2.376 |
| WBGene00000111 | WBGene00038231 | 76.73 | 437 | 0.1493 | 2.376 |
| WBGene00006388 | WBGene00035774 | 43.75 | 254 | 0.6019 | 5.098 |
| WBGene00006389 | WBGene00026403 | 43.75 | 236 | 0.6019 | 5.098 |
| WBGene00016589 | WBGene00028571 | 48.76 | 609 | 0.4708 | 9.3943 |
| WBGene00022610 | WBGene00030119 | 48.76 | 625 | 0.4708 | 9.3943 |
| WBGene00008924 | WBGene00023609 | 67.74 | 531 | 0.2219 | 18.3691 |
| WBGene00010077 | WBGene00041654 | 67.74 | 588 | 0.2219 | 18.3691 |
| WBGene00022717 | WBGene00035644 | 59.56 | 927 | 0.2786 | 1.1239 |
| WBGene00006484 | WBGene00035644 | 59.56 | 362 | 0.2786 | 1.1239 |
| WBGene00001834 | WBGene00027230 | 60.35 | 461 | 0.3118 | 10.0588 |
| WBGene00001836 | WBGene00038066 | 60.35 | 465 | 0.3118 | 10.0588 |
| WBGene00004439 | WBGene00026823 | 70.55 | 146 | 0.2435 | 11.5091 |
| WBGene00004438 | WBGene00034993 | 70.55 | 147 | 0.2435 | 11.5091 |
| WBGene00007554 | WBGene00041499 | 54.84 | 557 | 0.3725 | 18.331 |
| WBGene00012348 | WBGene00041499 | 54.84 | 542 | 0.3725 | 18.331 |
| WBGene00007350 | WBGene00029579 | 76.18 | 322 | 0.1816 | 10.7378 |
| WBGene00017759 | WBGene00035499 | 76.18 | 321 | 0.1816 | 10.7378 |
| WBGene00016934 | WBGene00035192 | 42.74 | 480 | 0.5699 | 9.4187 |
| WBGene00007446 | WBGene00023764 | 42.74 | 540 | 0.5699 | 9.4187 |
| WBGene00003561 | WBGene00034955 | 30.9 | 1383 | 0.773 | 10.3148 |
| WBGene00003562 | WBGene00036534 | 30.9 | 1274 | 0.773 | 10.3148 |
| WBGene00000217 | WBGene00027753 | 47.31 | 444 | 0.5178 | 1.9277 |
| WBGene00000216 | WBGene00032145 | 47.31 | 398 | 0.5178 | 1.9277 |
| WBGene00009976 | WBGene00032590 | 49.08 | 478 | 0.4572 | 19.1938 |
| WBGene00009977 | WBGene00032589 | 49.08 | 388 | 0.4572 | 19.1938 |
| WBGene00018901 | WBGene00028243 | 64.08 | 309 | 0.2624 | 12.798 |
| WBGene00008979 | WBGene00029624 | 64.08 | 300 | 0.2624 | 12.798 |
| WBGene00020052 | WBGene00030797 | 66.01 | 153 | 0.2779 | 11.4635 |
| WBGene00012968 | WBGene00037681 | 66.01 | 168 | 0.2779 | 11.4635 |
| WBGene00009140 | WBGene00040807 | 42.77 | 306 | 0.5372 | 17.4543 |
| WBGene00008693 | WBGene00036716 | 42.77 | 166 | 0.5372 | 17.4543 |
| WBGene00004443 | WBGene00040965 | 72.58 | 62 | 0.2537 | 0.6483 |
| WBGene00012956 | WBGene00040965 | 72.58 | 87 | 0.2537 | 0.6483 |
| WBGene00011530 | WBGene00025898 | 45.16 | 1490 | 0.4823 | 6.59 |
| WBGene00016700 | WBGene00037226 | 45.16 | 1432 | 0.4823 | 6.59 |
| WBGene00012149 | WBGene00026061 | 54.89 | 400 | 0.4132 | 14.0745 |
| WBGene00009453 | WBGene00033328 | 54.89 | 372 | 0.4132 | 14.0745 |
| WBGene00019207 | WBGene00029242 | 50.2 | 492 | 0.4381 | 9.7792 |
| WBGene00019979 | WBGene00028696 | 50.2 | 516 | 0.4381 | 9.7792 |
| WBGene00003938 | WBGene00034255 | 87.54 | 351 | 0.0757 | 0.3888 |
| WBGene00001204 | WBGene00034255 | 87.54 | 289 | 0.0757 | 0.3888 |
| WBGene00004217 | WBGene00033020 | 51.95 | 933 | 0.4029 | 14.7528 |
| WBGene00004224 | WBGene00035294 | 51.95 | 900 | 0.4029 | 14.7528 |
| WBGene00004256 | WBGene00024751 | 52.88 | 997 | 0.3902 | 8.1971 |
| WBGene00004257 | WBGene00037136 | 52.88 | 1328 | 0.3902 | 8.1971 |
| WBGene00004995 | WBGene00037909 | 26.79 | 238 | 0.9051 | 2.3893 |
| WBGene00004993 | WBGene00037906 | 26.79 | 402 | 0.9051 | 2.3893 |
| WBGene00000250 | WBGene00032644 | 33.77 | 308 | 0.7694 | 5.6259 |
| WBGene00000249 | WBGene00041676 | 33.77 | 155 | 0.7694 | 5.6259 |
| WBGene00011850 | WBGene00042135 | 34.77 | 608 | 0.7347 | 12.8928 |
| WBGene00009622 | WBGene00036912 | 34.77 | 557 | 0.7347 | 12.8928 |
| WBGene00001638 | WBGene00036196 | 57.97 | 449 | 0.3122 | 10.7784 |
| WBGene00001639 | WBGene00031306 | 57.97 | 437 | 0.3122 | 10.7784 |
| WBGene00009176 | WBGene00038684 | 45.41 | 223 | 0.4777 | 4.2335 |
| WBGene00012295 | WBGene00036217 | 45.41 | 220 | 0.4777 | 4.2335 |
| WBGene00014170 | WBGene00034158 | 65.14 | 109 | 0.3114 | 5.8458 |
| WBGene00010763 | WBGene00034158 | 65.14 | 132 | 0.3114 | 5.8458 |
| WBGene00000964 | WBGene00026309 | 72.16 | 520 | 0.1811 | 12.6999 |
| WBGene00000963 | WBGene00040591 | 72.16 | 489 | 0.1811 | 12.6999 |
| WBGene00015406 | WBGene00040591 | 37.41 | 160 | 0.6889 | 10.4543 |
| WBGene00007419 | WBGene00025733 | 37.41 | 153 | 0.6889 | 10.4543 |
| WBGene00000002 | WBGene00039142 | 69.25 | 493 | 0.232 | 11.3918 |
| WBGene00000004 | WBGene00025215 | 69.25 | 493 | 0.232 | 11.3918 |
| WBGene00008885 | WBGene00033192 | 32.04 | 399 | 0.7899 | 13.6314 |
| WBGene00016044 | WBGene00038480 | 32.04 | 427 | 0.7899 | 13.6314 |
| WBGene00003041 | WBGene00040585 | 40.69 | 491 | 0.5917 | 8.9133 |
| WBGene00021661 | WBGene00040585 | 40.69 | 544 | 0.5917 | 8.9133 |
| WBGene00016112 | WBGene00025527 | 82.52 | 148 | 0.1001 | 1.0433 |
| WBGene00016123 | WBGene00025527 | 82.52 | 143 | 0.1001 | 1.0433 |
| WBGene00000516 | WBGene00033806 | 30.39 | 184 | 0.9201 | 9.4085 |
| WBGene00000517 | WBGene00033805 | 30.39 | 258 | 0.9201 | 9.4085 |
| WBGene00017757 | WBGene00035494 | n.d. | 214 | n.d. | n.d. |
| WBGene00010029 | WBGene00035494 | n.d. | 183 | n.d. | n.d. |
| WBGene00001101 | WBGene00025421 | 44.07 | 539 | 0.5445 | 11.2613 |
| WBGene00001102 | WBGene00025391 | 44.07 | 654 | 0.5445 | 11.2613 |
| WBGene00016507 | WBGene00030548 | 77.18 | 444 | 0.1406 | 1.4609 |
| WBGene00016506 | WBGene00030548 | 77.18 | 355 | 0.1406 | 1.4609 |
| WBGene00019608 | WBGene00025657 | 53.24 | 1009 | 0.3885 | 6.7028 |
| WBGene00012835 | WBGene00025657 | 53.24 | 709 | 0.3885 | 6.7028 |
| WBGene00000509 | WBGene00035361 | 43.06 | 474 | 0.5615 | 9.8317 |
| WBGene00000510 | WBGene00034909 | 43.06 | 429 | 0.5615 | 9.8317 |
| WBGene00022801 | WBGene00033234 | 40.08 | 507 | 0.5947 | 4.2682 |
| WBGene00003956 | WBGene00023546 | 40.08 | 565 | 0.5947 | 4.2682 |
| WBGene00016074 | WBGene00029229 | 85.21 | 610 | 0.0802 | 0.1931 |
| WBGene00016072 | WBGene00029229 | 85.21 | 142 | 0.0802 | 0.1931 |
| WBGene00004052 | WBGene00036378 | 54.04 | 485 | 0.3565 | 2.9597 |
| WBGene00004051 | WBGene00036378 | 54.04 | 781 | 0.3565 | 2.9597 |
| WBGene00004025 | WBGene00028556 | 56.45 | 539 | 0.366 | 19.8415 |
| WBGene00001077 | WBGene00033991 | 56.45 | 559 | 0.366 | 19.8415 |
| WBGene00019427 | WBGene00025675 | 44.03 | 464 | 0.5459 | 9.8457 |
| WBGene00017178 | WBGene00034951 | 44.03 | 578 | 0.5459 | 9.8457 |
| WBGene00004183 | WBGene00031372 | 35.24 | 441 | 0.737 | 12.0161 |
| WBGene00004182 | WBGene00036415 | 35.24 | 566 | 0.737 | 12.0161 |
| WBGene00001813 | WBGene00042202 | 52.01 | 733 | 0.3978 | 8.8637 |
| WBGene00001811 | WBGene00023795 | 52.01 | 586 | 0.3978 | 8.8637 |
| WBGene00011578 | WBGene00026107 | 34.83 | 393 | 0.734 | 9.7753 |
| WBGene00020727 | WBGene00036051 | 34.83 | 362 | 0.734 | 9.7753 |
| WBGene00002064 | WBGene00031496 | 83.85 | 195 | 0.1043 | 8.6519 |
| WBGene00002065 | WBGene00023941 | 83.85 | 161 | 0.1043 | 8.6519 |
| WBGene00004801 | WBGene00029720 | 75.26 | 287 | 0.1346 | 0.9746 |
| WBGene00004802 | WBGene00029720 | 75.26 | 287 | 0.1346 | 0.9746 |
| WBGene00016811 | WBGene00029166 | 34.86 | 319 | 0.693 | 4.577 |
| WBGene00022489 | WBGene00035475 | 34.86 | 302 | 0.693 | 4.577 |
| WBGene00020215 | WBGene00029977 | 34.39 | 297 | 0.7114 | 15.8038 |
| WBGene00012142 | WBGene00041856 | 34.39 | 299 | 0.7114 | 15.8038 |
| WBGene00020490 | WBGene00026875 | 41.05 | 1285 | 0.5963 | 8.9123 |
| WBGene00015177 | WBGene00036193 | 41.05 | 1203 | 0.5963 | 8.9123 |
| WBGene00015676 | WBGene00036248 | 69.93 | 594 | 0.2024 | 1.4083 |
| WBGene00020168 | WBGene00036248 | 69.93 | 556 | 0.2024 | 1.4083 |
| WBGene00000107 | WBGene00041442 | 82.16 | 510 | 0.1059 | 3.7686 |
| WBGene00000108 | WBGene00041442 | 82.16 | 537 | 0.1059 | 3.7686 |
| WBGene00007955 | WBGene00036973 | 49.54 | 255 | 0.4342 | 4.1253 |
| WBGene00009238 | WBGene00036973 | 49.54 | 216 | 0.4342 | 4.1253 |
| WBGene00016943 | WBGene00033562 | 68.26 | 427 | 0.2209 | 13.3719 |
| WBGene00019433 | WBGene00033551 | 68.26 | 419 | 0.2209 | 13.3719 |
| WBGene00003001 | WBGene00029035 | 47.88 | 1429 | 0.4414 | 17.1208 |
| WBGene00001609 | WBGene00029022 | 47.88 | 1295 | 0.4414 | 17.1208 |
| WBGene00007258 | WBGene00025031 | 69.21 | 920 | 0.2013 | 0.831 |
| WBGene00021214 | WBGene00025031 | 69.21 | 440 | 0.2013 | 0.831 |
| WBGene00013268 | WBGene00039831 | 55.04 | 313 | 0.3707 | 1.6525 |
| WBGene00013255 | WBGene00039835 | 55.04 | 258 | 0.3707 | 1.6525 |
| WBGene00015203 | WBGene00033834 | 53.23 | 719 | 0.3921 | 10.796 |
| WBGene00013917 | WBGene00033834 | 53.23 | 763 | 0.3921 | 10.796 |
| WBGene00001501 | WBGene00033041 | 78.82 | 170 | 0.1369 | 2.3426 |
| WBGene00001500 | WBGene00024156 | 78.82 | 170 | 0.1369 | 2.3426 |
| WBGene00004410 | WBGene00024970 | 65.09 | 110 | 0.3599 | 1.9275 |
| WBGene00016493 | WBGene00033667 | 65.09 | 107 | 0.3599 | 1.9275 |
| WBGene00000387 | WBGene00033667 | 36.42 | 480 | 0.6914 | 9.9438 |
| WBGene00000388 | WBGene00030028 | 36.42 | 316 | 0.6914 | 9.9438 |
| WBGene00018008 | WBGene00026765 | 47.74 | 245 | 0.5575 | 1.6927 |
| WBGene00018354 | WBGene00025735 | 47.74 | 374 | 0.5575 | 1.6927 |
| WBGene00004345 | WBGene00023576 | 65.27 | 169 | 0.2454 | 7.7218 |
| WBGene00004344 | WBGene00031423 | 65.27 | 201 | 0.2454 | 7.7218 |
| WBGene00004969 | WBGene00040884 | 64.01 | 1219 | 0.27 | 18.9485 |
| WBGene00002041 | WBGene00034297 | 64.01 | 1219 | 0.27 | 18.9485 |
| WBGene00008767 | WBGene00040564 | 43.4 | 373 | 0.4964 | 18.2245 |
| WBGene00012786 | WBGene00037222 | 43.4 | 341 | 0.4964 | 18.2245 |
| WBGene00013024 | WBGene00027270 | 59.45 | 465 | 0.355 | 8.2817 |
| WBGene00009057 | WBGene00023571 | 59.45 | 406 | 0.355 | 8.2817 |
| WBGene00003407 | WBGene00030001 | 73.85 | 1528 | 0.1707 | 1.9256 |
| WBGene00003408 | WBGene00030002 | 73.85 | 1525 | 0.1707 | 1.9256 |
| WBGene00020511 | WBGene00025199 | 33.23 | 679 | 0.8212 | 9.9764 |
| WBGene00012315 | WBGene00042487 | 33.23 | 654 | 0.8212 | 9.9764 |
| WBGene00016061 | WBGene00030975 | 28.87 | 527 | 0.8767 | 11.3615 |
| WBGene00000139 | WBGene00027980 | 28.87 | 483 | 0.8767 | 11.3615 |
| WBGene00010115 | WBGene00029442 | 58.36 | 269 | 0.3395 | 18.0476 |
| WBGene00012928 | WBGene00034008 | 58.36 | 274 | 0.3395 | 18.0476 |
| WBGene00015623 | WBGene00033580 | 41.18 | 289 | 0.5125 | 16.2805 |
| WBGene00020402 | WBGene00026690 | 41.18 | 325 | 0.5125 | 16.2805 |
| WBGene00001025 | WBGene00032209 | 53.07 | 491 | 0.4279 | 11.2808 |
| WBGene00001046 | WBGene00026912 | 53.07 | 494 | 0.4279 | 11.2808 |
| WBGene00013739 | WBGene00026406 | 39.68 | 518 | 0.6165 | 10.917 |
| WBGene00007549 | WBGene00041750 | 39.68 | 531 | 0.6165 | 10.917 |
| WBGene00020649 | WBGene00041068 | 15.08 | 881 | 1.4424 | 12.1645 |
| WBGene00011258 | WBGene00037195 | 15.08 | 525 | 1.4424 | 12.1645 |
| WBGene00016642 | WBGene00029946 | 33.06 | 371 | 0.7766 | 5.5652 |
| WBGene00019980 | WBGene00028823 | 33.06 | 388 | 0.7766 | 5.5652 |
| WBGene00018152 | WBGene00030164 | 52.59 | 731 | 0.4076 | 15.201 |
| WBGene00016716 | WBGene00035178 | 52.59 | 718 | 0.4076 | 15.201 |
| WBGene00018738 | WBGene00034705 | 9.39 | 421 | 1.9419 | 6.8103 |
| WBGene00015388 | WBGene00035101 | 9.39 | 362 | 1.9419 | 6.8103 |
| WBGene00014220 | WBGene00031541 | 33.68 | 784 | 0.6728 | 14.1144 |
| WBGene00003504 | WBGene00031539 | 33.68 | 910 | 0.6728 | 14.1144 |
| WBGene00018755 | WBGene00037863 | 71.46 | 530 | 0.1979 | 1.7561 |
| WBGene00012914 | WBGene00037848 | 71.46 | 529 | 0.1979 | 1.7561 |
| WBGene00009514 | WBGene00025907 | 32.13 | 277 | 0.8221 | 12.9654 |
| WBGene00004135 | WBGene00042179 | 32.13 | 319 | 0.8221 | 12.9654 |
| WBGene00021296 | WBGene00038818 | 45.49 | 297 | 0.4853 | 12.3804 |
| WBGene00019022 | WBGene00025405 | 45.49 | 278 | 0.4853 | 12.3804 |
| WBGene00003368 | WBGene00025104 | 63.51 | 363 | 0.2308 | 2.733 |
| WBGene00012162 | WBGene00025104 | 63.51 | 359 | 0.2308 | 2.733 |
| WBGene00004244 | WBGene00033942 | 41.28 | 535 | 0.5924 | 14.899 |
| WBGene00004245 | WBGene00035164 | 41.28 | 703 | 0.5924 | 14.899 |
